# Key gene networks that control magnetosome biomineralization in magnetotactic bacteria

**DOI:** 10.1093/nsr/nwac238

**Published:** 2022-10-28

**Authors:** Peiyu Liu, Yue Zheng, Rongrong Zhang, Jinling Bai, Kelei Zhu, Karim Benzerara, Nicolas Menguy, Xiang Zhao, Andrew P Roberts, Yongxin Pan, Jinhua Li

**Affiliations:** Key Laboratory of Earth and Planetary Physics, Institute of Geology and Geophysics, Innovation Academy for Earth Science, Chinese Academy of Sciences, Beijing 100029, China; Laboratory for Marine Geology, Qingdao National Laboratory for Marine Science and Technology, Qingdao 266061, China; Southern Marine Science and Engineering Guangdong Laboratory, Zhuhai 519082, China; College of Earth and Planetary Sciences, University of Chinese Academy of Sciences, Beijing 100049, China; State Key Laboratory of Marine Environmental Science/College of the Environment and Ecology, Xiamen University, Xiamen 361102, China; Key Laboratory of Earth and Planetary Physics, Institute of Geology and Geophysics, Innovation Academy for Earth Science, Chinese Academy of Sciences, Beijing 100029, China; Laboratory for Marine Geology, Qingdao National Laboratory for Marine Science and Technology, Qingdao 266061, China; Southern Marine Science and Engineering Guangdong Laboratory, Zhuhai 519082, China; College of Earth and Planetary Sciences, University of Chinese Academy of Sciences, Beijing 100049, China; Key Laboratory of Earth and Planetary Physics, Institute of Geology and Geophysics, Innovation Academy for Earth Science, Chinese Academy of Sciences, Beijing 100029, China; Laboratory for Marine Geology, Qingdao National Laboratory for Marine Science and Technology, Qingdao 266061, China; Southern Marine Science and Engineering Guangdong Laboratory, Zhuhai 519082, China; College of Earth and Planetary Sciences, University of Chinese Academy of Sciences, Beijing 100049, China; Key Laboratory of Earth and Planetary Physics, Institute of Geology and Geophysics, Innovation Academy for Earth Science, Chinese Academy of Sciences, Beijing 100029, China; Laboratory for Marine Geology, Qingdao National Laboratory for Marine Science and Technology, Qingdao 266061, China; Southern Marine Science and Engineering Guangdong Laboratory, Zhuhai 519082, China; College of Earth and Planetary Sciences, University of Chinese Academy of Sciences, Beijing 100049, China; Sorbonne Université, UMR CNRS 7590, MNHN, IRD, Institut de Minéralogie, de Physique des Matériaux et de Cosmochimie, IMPMC, Paris 75005, France; Sorbonne Université, UMR CNRS 7590, MNHN, IRD, Institut de Minéralogie, de Physique des Matériaux et de Cosmochimie, IMPMC, Paris 75005, France; Research School of Earth Sciences, Australian National University, Canberra ACT 2601, Australia; Research School of Earth Sciences, Australian National University, Canberra ACT 2601, Australia; Key Laboratory of Earth and Planetary Physics, Institute of Geology and Geophysics, Innovation Academy for Earth Science, Chinese Academy of Sciences, Beijing 100029, China; College of Earth and Planetary Sciences, University of Chinese Academy of Sciences, Beijing 100049, China; Key Laboratory of Earth and Planetary Physics, Institute of Geology and Geophysics, Innovation Academy for Earth Science, Chinese Academy of Sciences, Beijing 100029, China; Laboratory for Marine Geology, Qingdao National Laboratory for Marine Science and Technology, Qingdao 266061, China; College of Earth and Planetary Sciences, University of Chinese Academy of Sciences, Beijing 100049, China

**Keywords:** magnetotactic bacteria, magnetosome biomineralization, chain assembly, gene networks, integrative genomics and phenomics

## Abstract

Magnetotactic bacteria (MTB) are a group of phylogenetically and morphologically diverse prokaryotes that have the capability of sensing Earth's magnetic field via nanocrystals of magnetic iron minerals. These crystals are enclosed within intracellular membranes or organelles known as magnetosomes and enable a sensing function known as magnetotaxis. Although MTB were discovered over half a century ago, the study of the magnetosome biogenesis and organization remains limited to a few cultured MTB strains. Here, we present an integrative genomic and phenomic analysis to investigate the genetic basis of magnetosome biomineralization in both cultured and uncultured strains from phylogenetically diverse MTB groups. The magnetosome gene contents/networks of strains are correlated with magnetic particle morphology and chain configuration. We propose a general model for gene networks that control/regulate magnetosome biogenesis and chain assembly in MTB systems.

## Teaser

A general model for gene networks that control magnetosome biogenesis and chain assembly in magnetotactic bacteria is proposed by a comparative genomic and phenomic analysis.

## INTRODUCTION

Magnetotactic bacteria (MTB) are phylogenetically and morphologically diverse prokaryotes that share an ancestral capability of producing intracellular magnetite (Fe_3_O_4_) or/and greigite (Fe_3_S_4_) nanocrystals within organelles called magnetosomes [1]. Magnetosomes are often organized into one chain or several chains [1,2]. By using these dedicated magnetic organelles, MTB can efficiently shuttle up and down in the oxic–anoxic transition zone of aquatic environments by swimming along Earth's magnetic field lines. This process was initially named magnetotaxis and was later modified to magneto-aerotaxis/chemotaxis [3–5]. Deciphering magnetosome biogenesis and assembly in MTB is critical for understanding the mechanism of biologically controlled mineralization of magnetic iron minerals and the evolution of magnetoreception in organisms [6,7]. This biomineralization has also bio-inspired magnetic nanochain synthesis for nanotechnological and biomedical applications [8,9]. Furthermore, the fossil remains of MTB (i.e. magnetofossils) preserved in sediments or sedimentary rocks are used widely for paleomagnetic and paleoenvironmental analyses [10–12].

MTB are phylogenetically affiliated with the *Alphaproteobacteria, Gammaproteobacteria* and *Candidatus* Etaproteobacteria classes in the *Pseudomonadota* (synonym *Proteobacteria*) phylum [13] and the *Desulfobacterota, Nitrospirota* (synonym *Nitrospirae*) [13] and *Candidatus Omnitrophica* phyla [2,14–16], and even possibly with other taxonomic lineages across the bacteria domain [17,18]. Both the morphology of magnetosome crystals and the content of magnetosome genes vary among taxonomic groups or even species/strains [14,19–24]. Although the functions of a few magnetosome genes have been analysed through *in vivo* and *in vitro* experiments in a few cultured strains MC-1, RS-1 and BW-1 [25–27], most progress in systematically understanding magnetosome formation relies on two genetically tractable strains: *Magnetospirillum magneticum* AMB-1 and *Magnetospirillum gryphiswaldense* MSR-1, which are affiliated with the genus *Magnetospirillum* of the *Alphaproteobacteria* class [6,28]. Both strains form cuboctahedral magnetite particles that are organized into a single chain. Considering the phylogenetic diversity of uncultured MTB and their diverse magnetic crystal morphologies and chain assemblies [2,14,19,20], a general model for gene networks that control or regulate magnetic particle biogenesis and chain assembly is still lacking; such a model cannot be determined from a limited number of cultured MTB strains alone. Therefore, a culture-independent comprehensive study of MTB from different taxonomic groups is required and is presented here to evaluate the roles of gene networks in determining their crystal morphology and chain assembly.

## RESULTS AND DISCUSSION

### Workflow for a genomic and phenomic study of uncultured MTB

**Figure 1.**
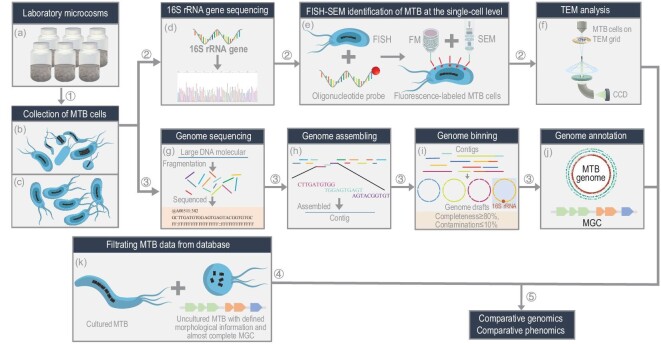
Five-step workflow for genomic and phenomic study of uncultured MTB. Each step is designed to obtain a specific piece of information. In Step 1, living MTB cells are collected from (a) laboratory microcosms, generally using homemade magnetic separation apparatus or capillary racetrack method. By magnetic separation, living MTB can be collected in sufficient amounts for further morphological and molecular biological studies. Some collections contain different MTB strain types (b), whereas other collections are dominated by one strain type (c). In Step 2, uncultured MTB are identified and characterized at the single-cell level [31], using (d) 16S rRNA gene sequencing of magnetically collected MTB cells, (e) fluorescence *in situ* hybridization (FISH) of targeted MTB cells with species-specific oligonucleotide probe and coordinated fluorescence microscopy (FM) and scanning electron microscopy (SEM) observations of probe-hybridized MTB cells. This step is generally followed by (f) transmission electron microscope (TEM) analyses, which provide morphological and chemical information on both cells and intracellular magnetic particles down to the atomic scale. Step 3 consists of genomic analyses and generally involves MTB cell (g) genome sequencing, (h) assembling, (i) binning and (j) annotation. Step 4 consists of (k) selecting MTB genome data from public databases. All cultured and uncultured MTB strains with defined morphological information and almost complete magnetosome gene clusters (MGCs) were filtered from the NCBI database. Step 5 consists of integrating genomic and phenomic analyses of cultured and uncultured MTB to understand magnetic particle biogenesis and chain organization within phylogenetically different MTB.

The workflow used here is shown in Fig. 1. Diverse living MTB were collected magnetically from laboratory microcosms of water and sediment from lakes or salt ponds using homemade magnetic separation apparatus (Fig. 1a–c and Supplementary Table S1) [29,30]. Molecular analysis of 16S rRNA gene sequences indicates that five MTB strains (tentatively named YQV-1, WYHS-4, YQC-5, YQR-1 and YQC-9) from the magnetic collections are novel species because they share low sequence identity (<97%) with known bacterial sequences (Fig. 1d and Supplementary Table S2). Ten other strains share a relatively high similarity (>98.7%) with previously reported 16S rRNA gene sequences of MTB; three (tentatively named XQGC-1, MYC-9 and MYC-10) have yet to be identified morphologically (Supplementary Table S2). Therefore, these new and morphologically unknown MTB strains were identified phylogenetically and structurally via a correlative fluorescence *in situ* hybridization (FISH) and scanning election microscopy (SEM) (FISH–SEM) approach at the single-cell level (Fig. 1e) [31] and were then characterized at the nanometre scale by transmission electron microscopy (TEM) (Fig. 1f and Supplementary Figs S1–S8).

TEM observations of magnetite-producing strains reveal that the magnetite has diverse crystal morphologies and chain configurations (Supplementary Table S3). Seven MTB strains were previously well characterized (Tables S2 and S3, and Supplementary Figs S9–S15). Here the 15 analysed MTB strains are affiliated phylogenetically with (i) the *Alphaproteobacteria* (i.e. YQV-1, WYHS-4), *Gammaproteobacteria* (SHHR-1) and *Candidatus* Etaproteobacteria (i.e. WYHC-3, MYC-9, YQC-3, YQC-5, YQC-9, DMHC-1, DMHC-6, DMHC-8, THC-1 and XQGC-1) classes of the *Pseudomonadota* phylum and (ii) the *Nitrospirota* phylum (i.e. YQR-1 and MYC-10) (Supplementary Fig. S16).

**Figure 2.**
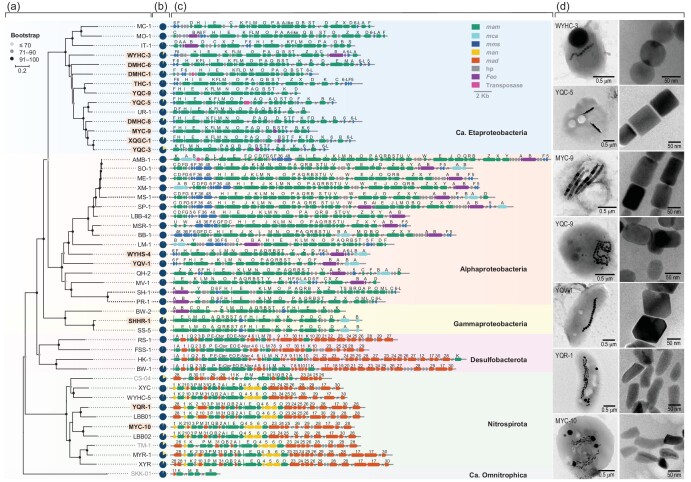
Phylogenetic, genetic and morphological information for both cultured and uncultured MTB. Comparison of the 15 studied strains (bold font with light orange background) (Supplementary Figs S1–15) with 32 data sets from previous studies (Supplementary Table S3). (a) Maximum-likelihood phylogenetic tree based on concatenated alignment of 120 ubiquitous conserved proteins. Strain names in bold and yellow background; white background and grey font represent genomes reported in this study, reported previously and containing high contamination (>10%), respectively. The genome of *Candidatus* Omnitrophus magneticus strain SKK-01 was used to root the tree. (b) Pie charts of genome completeness evaluated using checkM v1.0.12 (Supplementary Table S3). Dark blue and yellow regions represent the percentage of present and absent genes, respectively. (c) Comparison of MGCs from MTB strains with distinct lineages. (d) TEM images of representative MTB strains (left column) and their magnetic particles (right column).

The genomes of the 15 MTB strains were sequenced using Illumina MiSeq (Fig. 1g), assembled (Fig. 1h), binned (Fig. 1i) and annotated (Fig. 1j) (Supplementary Figs S1–S15). Most of the obtained genomes are high-quality drafts (with >90% completeness, <5% contamination) except for four strains (YQC-3, XQGC-1, WYHC-3 and SHHR-1), which are medium-quality drafts (with >50% completeness, <10% contamination) [32]. Their sizes range from 3.2 to 5.7 Mb and GC contents range from 42.3% to 66.2% (Supplementary Figs S1–S15 and Supplementary Table S3). Genome annotations for all 15 genomes contain large regions with most genes previously shown to be implicated in magnetosome formation [6,22–24], i.e. magnetosome gene clusters (MGCs) (Figs 1j and 2). Gene sequence comparisons using the basic local alignment search tool reveal that genes in the MGCs are homologous with the *mam, mms, mad* or *man* family genes that have been identified in different taxonomic MTB groups [17,22,33].

Overall, we obtained 15 data sets, each corresponding to an uncultured MTB strain and containing corresponding genomic and magnetosome morphological information. The 15 data sets, together with 32 others corresponding to previously reported and well-characterized cultured and uncultured MTB strains (Figs 1k and 2, and Supplementary Tables S3–S5), provide a database for combined analyses of comparative genomics and phenomics to understand magnetosome magnetic particle biogenesis and chain assembly among different taxonomic MTB groups (Figs 1 and 2).

### Variations in magnetosome gene content in MTB genomes

We first analysed the content of magnetosome genes inside and outside the MGCs for different taxonomic MTB groups (Fig. 2 and Supplementary Figs S17 and S18). Eight *mam* genes (i.e. *mamA, B, E, I, K, M, P* and *Q*) are present in the MGCs of all 47 inspected MTB strains. They are core genes that control the universal processes of magnetosome formation in MTB (i.e. biogenesis and magnetosome membrane assembly, iron uptake, magnetite nucleation and magnetosome chain assembly) [6], hereafter termed magnetosome core genes (MACGs). By contrast, *mamL* and *mamO*, which were previously thought to be core genes [22,34], are absent in the phyla *Nitrospirota* and *Desulfobacterota*, respectively. Phylogenetic analysis further indicates that MamO sequences from MTB of the phyla *Pseudomonadota* and *Nitrospirota* form a distinct, monophyletic clade; the MamE-Nter, MamEO and MamE-Cter sequences from MTB of the *Desulfobacterota* phylum and MamE sequences from MTB of the phyla *Pseudomonadota* and *Nitrospirota* form another monophyletic clade (Supplementary Fig. S19). Consistently with a previous study [35], this indicates that the *mamE-Cter, mamEO* and *mamE-Nter* genes are homologous genes of *mamE* rather than *mamO*. Similarly, the *mamR, mcaA* and *mcaB* genes are only present in the *Alphaproteobacteria* and *Gammaproteobacteria* classes; *mamY* is only present in the *Alphaproteobacteria* class and the *mamJUVW* genes are found only in the *Magnetospirillum* genus of the *Alphaproteobacteria* class.

Several genes are more or less group-specific at the phylum level: four *mam* genes (i.e. mam*H, F, S* and T) are only present in the 29 inspected MTB strains of the *Pseudomonadota* phylum (hereafter MACGPs); several, but not all *mad* genes, are only present in four MTB strains of the *Desulfobacterota* phylum; several other *mad* genes (e.g. *mad2, mad23–26, mad28* and *mad29*) and the *man1–6* genes appear to be shared by MTB of the *Nitrospirota* phylum. This is consistent with previous observations [6,22,24,28,36]. Also, some MTB strains affiliated with the *Pseudomonadota* phylum contain *mms5, mms6, mms6-like* (*mms6-L*), *mms36, mms48* and *mamCDGXZ*.

We then compared the MGC organizations of the different taxonomic MTB groups (Fig. 2 and Supplementary Figs S20–S22). The MACGs of most MTB in the *Nitrospirota* phylum contain a conserved *mamAB*-like gene cluster that consists of all *mam* and *man1–6* genes, and several *mad* genes (i.e. *mad2, mad10, mad23–26* and *mad31*). By contrast, the other *mad* genes (e.g. *mad17* and *mad28*–3*0*) are scattered outside the MGCs (Supplementary Fig. S20). Strain CS-04 appears to be an exception with a distinctive gene order compared to the other *Nitrospirota* strains [15]. MTB in the *Desulfobacterota* phylum contain a conserved *mamAB*-like gene cluster consisting of some *mam* (i.e. *mamABEILMP*) and *mad1–9* genes. The other *mad* (e.g. *mad10–11* and *mad17–30*) and *mamK* genes are scattered outside the conserved gene cluster (Supplementary Fig. S21).

The organization and order of magnetosome genes within the *Pseudomonadota* phylum are much more diverse and even species-specific, possibly because of the much larger genome data set than for the *Desulfobacterota* and *Nitrospirota* phyla. In *Pseudomonadota*, most known magnetosome genes are organized into seven conserved gene clusters (i.e. *mamAB, mamAB-2, mms6, mamGFDC, mamXYZ, mcaAB* and *feoABm*). The *mamAB* gene cluster appears to be shared by all MTB strains of the *Pseudomonadota* phylum, while the other six gene clusters are distributed randomly (Supplementary Fig. S22). Furthermore, the *mamAB-2* gene cluster appears to be a remnant of a *mamAB* operon duplication and consists of *mam* genes (e.g. *mamEJO*) [33]. Genes in the *mms6, mamCDFG, mamXYZ, mcaAB* and *FeoABm* clusters are usually inserted into the *mamAB* gene cluster or are scattered outside the MGCs (Fig. 2 and Supplementary Fig. S22). In contrast to the presence or absence of whole gene clusters, some genes in the *mamAB* gene cluster could vary among different MTB strains possibly due to genomic events such as duplication, deletion and insertion (e.g. strains IT-1, SH-1 and BW-2) [33,37].

In brief, both magnetosome gene content and organization vary significantly among phyla and are relatively conserved within the same phylum [38]. This suggests that MGC variability should account for diverse crystal morphology and chain assembly of magnetosomes in phylogenetically different MTB groups.

### Genes that control/regulate magnetosome crystal morphology in the *Pseudomonadota, Desulfobacterota* and *Nitrospirota* phyla

The functions of genes involved in magnetosome biogenesis and chain assembly in cultured MTB strains have been studied by *in vivo* genetic, *in vitro* biochemical [6,26–28,36,39] (Supplementary Table S6) and *in silico* bioinformatic analyses [22,24] (Supplementary Table S7). Relying on these findings, we first conducted bioinformatic analyses to identify potential genes that (i)

were found to be essential for magnetosome formation in the *Pseudomonadota* phylum but (ii) are absent in the *Nitrospirota* and *Desulfobacterota* phyla (Supplementary Table S8). Our results suggest that (i) the Man2 protein may play a role in magnetosome membrane formation because it shares an ∼30% sequence similarity with the MamL protein [34,40]; (ii) the Mad23 protein may also play a role in sorting mad proteins to the magnetosome membrane because it contains a HEAT repeat domain [41] (Supplementary Fig. S23); and (iii) the Mad9 protein may also play a role in the redox control of magnetosome vesicles because it contains an iron–sulphur binding domain belonging to the bacterial-type ferredoxin protein family [42] (Supplementary Fig. S24).

**Figure 3.**
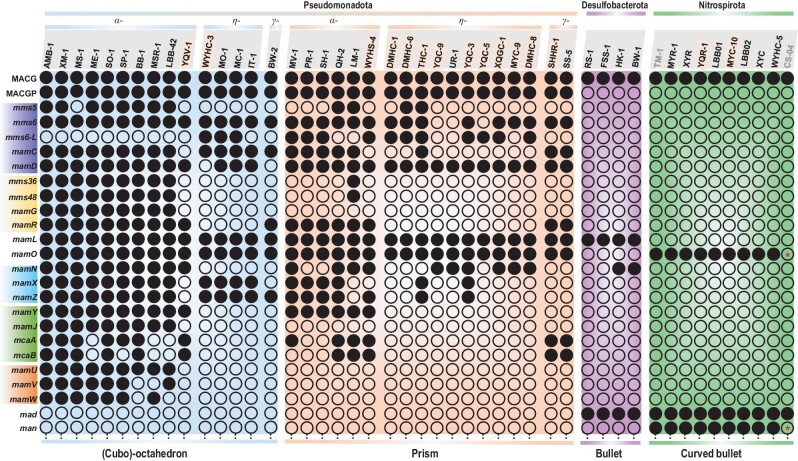
Relationship between magnetic particle morphology and magnetosome gene content. Genes with similar functions are highlighted by the same-coloured background (Supplementary Tables S6 and S7). Greek letters α, η and γ represent *Alphaproteobacteria, Candidatus* Etaproteobacteria and *Gammaproteobacteria* classes. MTB strains are shown in groups according to their phylogeny and magnetite crystal morphologies. Strain names in bold and yellow background represent genomes reported here; grey font represents high contamination (>10%). Black solid circles indicate the presence of the corresponding gene; black hollow circles correspond to its absence. Black hollow circles with a red asterisk inside indicate that the gene was not detected possibly due to incomplete genome sequencing. MACGs, magnetosome core genes in MTB system (i.e. *mamA, B, E, I, K, M, P* and Q). MACGPs, magnetosome genes only conserved in the *Pseudomonadota* phylum besides the MACGs (i.e. *mamH, F, S* and *T*).

We then focused on genes related to magnetosome magnetite crystal morphology in different taxonomic MTB groups (Fig. 3 and Supplementary Table S9). Magnetotactic *Pseudomonadota* generally form magnetite particles with octahedral morphologies ({111} faces), cuboctahedral ({111} + {100} faces) or prismatic ({111} + {110} + {100} faces) [19,20]. The Mms6 protein is thought to be essential for producing magnetite with cuboctahedral morphology by promoting growth of {110} faces that result in their disappearance in mature particles [23,43]. We confirm the absence of *mms6* in the *Desulfobacterota* and *Nitrospirota* phyla (Fig. 3) and find that some MTB in the *Pseudomonadota* phylum may contain an additional Mms6-L protein (conserve score 42.4%) with a region homologous to the AMB-1 Mms6 protein (Fig. 3 and Supplementary Fig. S25). However, both *mms6* and *mms6-L* are shared by not only all analysed MTB that form octahedral and cuboctahedral magnetite particles, but also some that form prismatic magnetite (Fig. 3). A possible explanation is that the Mms6 protein needs assistance from other unknown proteins that are absent in prismatic magnetite-forming MTB to form cuboctahedral magnetite. Alternatively, besides Mms6, other Mam (e.g. MamGFDC) or Mms (e.g. MmsF, -5, -6, -36 and -48) proteins could play a role in regulating the crystal morphology and grain size of magnetite in magnetotactic *Pseudomonadota* [23,44].

Absence of the abovementioned proteins in the *Desulfobacterota* and *Nitrospirota* phyla (Fig. 3) suggests that some Mad and Man proteins might perform similar functions in controlling magnetite crystal morphology in these groups. Bioinformatic analysis indicates that beside Mad10 and Mad11 proteins [26], Mad3–5, Mad8, Mad19, Man1, Man3 and Man4 also contain a hydrophilic terminal domain, rich in carboxyl and hydroxyl amino acid groups with a strong affinity for metal ions (Supplementary Figs S26 and S27). In the *Desulfobacterota* phylum, the Mad1 and Mad2 proteins have been suggested to be essential for crystallizing stable, bullet-shaped magnetite [27]. It remains unclear whether the *man-1, -3* and *-4* genes are related to curved bullet-shaped magnetosome magnetite. However, these three genes are only conserved in all MTB of the *Nitrospirota* phylum (Supplementary Fig. S18). This suggests that they might play roles in controlling/regulating crystal morphology [23,24,27] or chain assembly [45] of magnetite (Supplementary Table S9).

We also analysed the effects of MGC organization and gene order in MTB from the *Pseudomonadota* phylum (Supplementary Fig. S28). Except for the *mamAB*(-like) gene cluster, which is conserved in all MTB strains, other gene clusters are distributed randomly in either prismatic or cuboctahedral magnetite-forming MTB strains. This indicates that the organization and order of these gene clusters have little or no effect on crystal morphology.

### Genes that control/regulate magnetosome chain assembly in the *Pseudomonadota, Desulfobacterota* and *Nitrospirota* phyla

**Figure 4.**
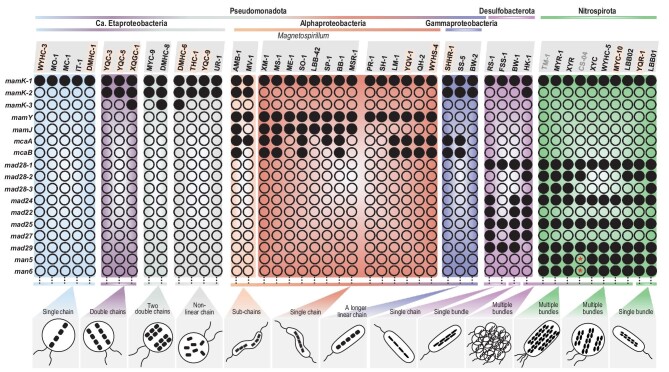
Relationship between magnetosome chain assembly and magnetosome gene content. MTB strains are shown in groups according to phylogeny and magnetosome chain configuration. Strain names in bold and yellow background represent genomes reported here; grey font represents high contamination (>10%). Black solid circles indicate the presence of the corresponding gene; black hollow circles indicate its absence. Black hollow circles with a red asterisk inside indicate that the gene was not detected possibly due to incomplete genome sequencing.

Self-assembly into chain-like structures is a hallmark that distinguishes magnetosome magnetite from other types of magnetite [12]. Our results reveal that the *mamK* gene is present in all analysed MTB strains, while *mcaA* and *mcaB* appear to be shared by some *Alphaproteobacteria* and *Gammaproteobacteria* MTB strains, *mad28* is conserved in MTB strains in the *Desulfobacterota* and *Nitrospirota* phyla, *mamY* is conserved in magnetotactic *Alphaproteobacteria* only and *mamJ* is present in the *Magnetospirillum* genus only (Fig. 4). This confirms the key role of *mamK* in magnetosome chain assembly [46,47] and suggests that some group-specific magnetosome genes (e.g. *mamJ, mamY, mcaA/B* and *mad28*) are responsible for diverse chain configurations in taxonomically different MTB groups. We explore below four further issues based on comparative genomic and phenomic analyses that need future exploration (Supplementary Table S10).

First, the copy number of *mamK* and the similarity of multicopy *mamK* genes appear to be related to magnetosome chain configuration in the *Pseudomonadota* phylum. For instance, all inspected MTB of the *Pseudomonadota* phylum with one *mamK* gene in their genomes appear to form a single magnetosome chain (Fig. 4). By contrast, MTB strains in *Candidatus* Etaproteobacteria that contain multicopy *mamK* genes with relatively high similarity (e.g. more than ∼67% of the protein sequence) produce double chains (e.g. YQC-5, YQC-3 and XQGC-1) or two double chains (e.g. DMHC-8) and those with relatively low similarity (e.g. less than ∼67% of the protein sequence) appear to assemble twisted or partial chains (e.g. YQC-9 and DMHC-6) or dispersed aggregates (e.g. THC-1 and UR-1) (Supplementary Fig. S29). It is unclear why magnetotactic *Gammaproteobacteria* with two *mamK* copies form only one linear chain along their cell long axis. A possible explanation is that the two *mamK* genes are located adjacently and therefore share one operon in the MGCs (Fig. 2 and Supplementary Fig. S22), which results in a longer MamK filament and a longer chain, as indicated by TEM observations [21,31,48]. In addition, strains AMB-1 and MV-1 have several magnetite sub-chains that are assembled linearly along the cell long axes [49,50]. However, in cryo-TEM observations, gaps are observed between sub-chains that are filled with empty magnetosome vesicles [39,51]. Recently, Wan and co-workers demonstrated that McaA and McaB create space for new magnetosome additions between pre-existing magnetosomes [51]. Despite having the *mcaA* and *mcaB* genes, some MTB from the *Alphaproteobacteria* and *Gammaproteobacteria* classes (e.g. strains YQV-1 and SHHR-1) do not form magnetosome sub-chains. A possible explanation is that their *mcaAB* genes are located outside the MGC and therefore are not involved in filling gaps between magnetosome sub-chains (Fig. 2 and Supplementary Fig. S17).

Second, bioinformatic analyses indicate that the *mad28* gene is homologous to the *mamK* gene. This suggests that Mad28 might be another actin-like protein that cooperates with MamK to regulate chain assembly in the *Desulfobacterota* and *Nitrospirota* phyla [22].

Third, both the Mad24 and Man5 proteins contain a protein domain homologous with a structural maintenance of chromosomes (SMC) domain (Supplementary Fig. S30). SMC domains are essential for chromosome transmission during genome replication and segregation in all organisms [52]. The Man5 protein has been proposed to play a role in multiple magnetosome chain arrangement and segregation during cell division [24]. We further suggest that the Mad24 and Man5 proteins might anchor magnetite particles into a chain bundle based on the following observations: (i) in single chain-forming *Desulfobacterota* MTB strains, the *mad24* gene is lost (e.g. strain FSS-1) or the SMC domain of the Mad24 protein is replaced by an ATPase domain (e.g. strain RS-1); (ii) both the C- and N-terminal domains of proteins Mad24 and Man5 contain acidic regions, which may be involved in binding magnetite (Supplementary Fig. S30). It is worth testing the absence of the *man5* gene in single chain-forming *Nitrospirota* MTB strains (e.g. strain HSMV-1) [53]. The Mad22, Mad25, Mad27, Mad29 and Man6 proteins all contain ATPase domains (Supplementary Figs S31 and S32), and their coding genes are located near the *mad24* or *man5* genes in MGCs. This suggests that they may work as different subunits of ATPase to provide energy for magnetosome chain bundle assembly (Fig. 2, and Supplementary Figs S20 and S21).

Fourth, we analysed the effects of MGC gene organization and order on magnetite chain configuration among MTB. Except for the *mamAB*(-like) gene cluster, which is conserved in all MTB strains, other gene clusters are distributed randomly in MTB strains from the *Pseudomonadota* (Supplementary Fig. S33). This indicates that, except for *mamK* and *mcaAB* genes that are closely related to magnetosome chain assembly, the organization and order of other genes in the MGC may have little or no effect on chain assembly.

### Gene networks for magnetosome biomineralization within the MTB system

**Figure 5.**
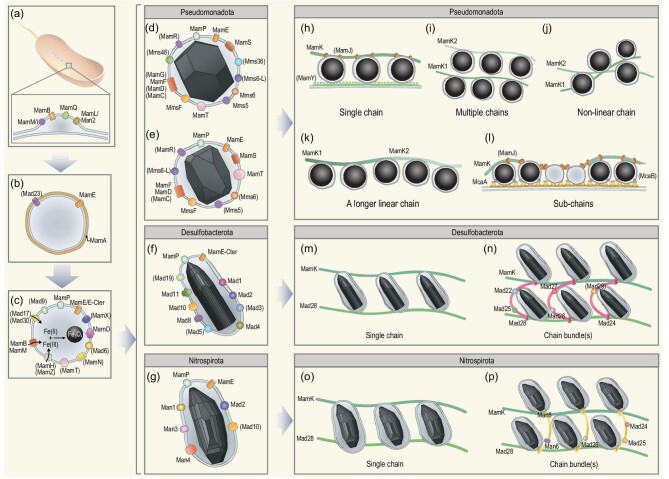
Conceptual model for the gene network responsible for magnetosome biomineralization. Proteins in brackets are not present in all analysed MTB strains. Magnetosome biomineralization can be divided into five steps that each involve a series of genes/proteins. (a) Magnetosome membrane formation. (b) Protein recruitment onto the magnetosome membrane. (c) Iron transportation and magnetite nucleation. Black arrows represent iron transport into the magnetosome vesicle. (d–g) Growth of (d) octahedral/cuboctahedral, (e) prismatic, (f) straight bullet-shaped and (g) curved bullet-shaped magnetite. (h–l) Chain assembly of magnetite in MTB belonging to the *Pseudomonadota* phylum with (h) single chain, (i) multiple chains, (j) non-linear chain, (k) a longer linear chain and (l) sub-chains; (m and n) chain assembly of magnetite in MTB from the *Desulfobacterota* phylum with (m) single chain and (n) chain bundle(s). (o and p) Chain assembly of magnetite in MTB from the *Nitrospirota* phylum with (o) single chain and (p) chain bundle(s). Black spheres enveloped by magnetosome membranes in (h–l) represent magnetite crystals with prismatic, octahedral or cuboctahedral morphology.

Stepwise magnetosome formation and chain assembly, and the responsible genes and proteins, are well documented in *Magnetospirillum* strains AMB-1 and MSR-1 [6,28]. Based on these foundational results, we tentatively propose a general model for the gene networks that control/regulate magnetosome biomineralization (Fig. 5):

- ‘Magnetosome membrane formation’. This is the first step in producing a structured protein–lipid complex that maintains a compatible chemico-physical environment required for magnetite biomineralization. It involves the MACGs *mamBIQL*/*man2* (*mamL* in the *Pseudomonadota* and *Desulfobacterota* phyla and *man2* in the *Nitrospirota* phylum) (Fig. 5a): the MamB protein induces membrane curvature [6]; MamM/I (MamM in MSR-1 and MamI in AMB-1), MamQ and MamL/Man2 proteins assist membrane formation [34,43].
- ‘Protein sorting’. Recruitment of specific proteins onto the magnetosome membrane needs several *man*/*mad* genes (Fig. 5b). MamA and MamE participate in sorting magnetosome-associated proteins (e.g. iron transport proteins, iron nucleation, pH and redox control proteins) to the membrane [39,54]. The Mad23 protein may also contribute to this process in the *Desulfobacterota* and *Nitrospirota* phyla.
- ‘Iron transportation and magnetite nucleation’. Once the magnetosome vesicle and protein sorting to the membrane are achieved, iron transport in and out of the vesicle is required for precipitation of the correct mineral. Besides the MamB and MamM proteins, the MamH and MamZ proteins are involved in iron uptake in the *Pseudomonadota* phylum [55], while the proteins Mad17 and Mad30 may play the same role in the *Desulfobacterota* and *Nitrospirota* phyla. The MamO protein promotes magnetite crystal nucleation [35,56] with the possible help of MamN in pH control [57] and several Mam (MamE/MamE-Cter, MamP, MamT, MamX and MamZ) and Mad (Mad6 and Mad9) proteins controlling the redox environment.
- ‘Crystal mineralization’. When optimal conditions are reached, a magnetite crystal starts to nucleate and grow within the magnetosome vesicle and finally achieves its species-specific morphology. The MamE protease (MamE-Cter in *Desulfobacterota*) also regulates magnetosome membrane and magnetite crystal growth [26,39]. The MamC, MamD, MamF, MamG, MamP, MamR, MamS, MamT, MmsF, Mms5, Mms6, Mms6-L, Mms36 and Mms48 proteins play a role in regulating magnetite grain size and morphology in the *Pseudomonadota* phylum [23,58] (Fig. 5d and e). Specifically, the Mms6 protein appears to be related to octahedral and cuboctahedral morphologies (Fig. 5d). Size regulation of bullet-shaped magnetite may involve MamP, Mad3–5, Mad8, Mad10, Mad11, Mad19, Man1, Man3 and Man4 (Fig. 5f and g). The Mad1 and Mad2 proteins are essential for morphological control of bullet-shaped magnetite in the *Desulfobacterota* phylum (Fig. 5f) [26], while Mad2, Man1, Man3 and Man4 may play a role in controlling the morphology of curved bullet-shaped magnetite in the *Nitrospirota* phylum (Fig. 5g).
- ‘Chain assembly’. MTB have diverse magnetosome chain assemblies such as single chain, multiple chains, chain bundles and even particle clusters or aggregates [14,46,47,59]. From our results, the copy number and content of the *mamK* gene may be responsible for magnetosome chain assembly in the *Pseudomonadota* phylum (Fig. 5h–l). The MTB strains with one *mamK* gene appear to form a single intact and linear magnetosome chain (e.g. strains WYHS-4 and DMHC-1) (Fig. 5h), while MTB strains in *Candidatus* Etaproteobacteria with multiple *mamK* copies tend to form multiple chains (e.g. strains YQC-5 and DMHC-8) (Fig. 5i) or a non-linear chain (e.g. strains DMHC-6 and THC-1) (Fig. 5j), which is likely related to the sequence similarity of MamK proteins. However, some MTB strains with multiple *mamK* copies also produce a longer linear chain possibly due to adjacent organization of multiple *mamK* gene copies in the MGC (e.g. strain SHHR-1) (Fig. 5k) or sub-chains form linearly along the cell long axis possibly because the McaA protein can create space for new magnetosome insertions between pre-existing ones and anchor magnetosomes onto the cytomembrane along the curvature line of spiral or vibrioid cells (e.g. strains AMB-1 and MV-1) (Fig. 5l) [49–51]. In the *Desulfobacterota* and *Nitrospirota* phyla, the actin-like MamK and Mad28 proteins assemble magnetosomes either as a single chain (Fig. 5m and o) or chain bundle with the help of the SMC family protein Mad24 or Man5 (Fig. 5n and p). The ATPase proteins Mad22, Mad25–27 and Man6 may provide energy for this process.

Magnetosome biomineralization and associated gene networks are diverse phylogenetically and far from being understood from genomic data alone. Identification and functional characterization of the genes in further cultured and uncultured MTB strains are crucial and accurate characterization is needed to understand the molecular mechanisms of magnetosome biomineralization in MTB.

## CONCLUSIONS AND IMPLICATIONS

By comparative genomic and phenomic analyses of both cultured and uncultured MTB strains, we demonstrate the presence of core magnetosome genes (i.e. MACGs) and phylum-specific magnetosome genes (e.g. MACGPs, *mad* and *man*) in the MTB system. This confirms that the magnetosome biomineralization capability might have had a common ancient origin in the bacteria domain that underwent subsequent lineage-specific evolution [38]. Moreover, it provides genetic evidence for the phylum-specific morphology of magnetosome magnetite [19,20]. Magnetofossil crystal morphology from the ancient geological record can, therefore, be a reliable proxy for the taxonomic lineage of ancient MTB and their paleoecology [10,19–21,60,61]. We also present a workflow for comparative genomic and phenomic analysis of cultured and uncultured MTB that enables us to propose a tentative general model for the gene networks that control/regulate magnetosome biogenesis and chain assembly in MTB. Although it remains incomplete, this conceptual model provides new insights into magnetosome gene function and chain assembly particularly for MTB other than magnetotactic *Magnetospirillum*. With this gene network, *in vivo* site-directed mutagenesis of cultured strains [34,39] or heterologous magnetosome gene expression [62] could be used in future to better understand molecular mechanisms of biogenesis and chain assembly of prismatic and bullet-shaped magnetite. Also, several proteins (e.g. Mad1 and Mad2) may provide pertinent targets for biomimetic synthesis of highly elongated magnetite nanoparticles (Fig. 5). Due to their significant shape anisotropy, such nanoparticles have higher magnetic coercivity than spherical or cuboctahedral ones [29,63], which could make them suitable for applications in nanomedicine and nanotechnology.

## DATA AVAILABILITY

The genome sequences obtained here have been deposited in the NCBI BioProject under accession number PRJNA657227 with BioSample numbers SAMN15825208 and SAMN15825210-SAMN15825223. The 16S rRNA gene sequences obtained here have been deposited in GenBank. MTB strains YQC-9, XQGC-1, MYC-9, YQC-5, WYHS-4, YQV-1, YQR-1 and MYC-10 are under accession numbers ON340520, ON340524, ON340531, ON340535-ON340538 and ON342894, respectively.

## Supplementary Material

nwac238_Supplemental_File
