# Supplementary material for: Key gene networks that control magnetosome biomineralization in magnetotactic bacteria
Source: Natl Sci Rev. 2022 Oct 28;10(1):nwac238. doi: 10.1093/nsr/nwac238 (PMC9840458; doi:10.1093/nsr/nwac238)
Supplement: nwac238_Supplemental_File [file nwac238_supplemental_file.pdf]

National Science Review

Supplementary Data for

Key gene networks that control magnetosome biomineralization in magnetotactic bacteria

Peiyu Liu#, Yue Zheng#, Karim Benzerara, Nicolas Menguy, Xiang Zhao, Andrew P. Roberts, Rongrong Zhang, Jinling Bai, Kelei Zhu, Yongxin Pan, Jinhua Li*

*Corresponding author: Jinhua Li (lijinhua@mail.iggcas.ac.cn)

#Peiyu Liu and Yue Zheng contributed equally to this work

This file includes:
Methods and materials
Figures S1-S33
Tables S1-S12
Supplementary references related to Supplementary Data.

Methods and materials

Sediment sampling, MTB collection, and sample preparation

Sediment sampling in the field and sample transfer and microcosm set-up in the laboratory followed a process described previously [1] (Table S1). MTB in microcosms were checked routinely with the hanging-drop technique [2] using an Olympus BX51 microscope equipped with phase-contrast, fluorescence, and a DP70 digital camera system (Olympus Corp., Tokyo, Japan). Living MTB cells were extracted magnetically from sediments in the microcosms with a homemade magnetic separation apparatus (Table S2) [3], washed three times with Milli-Q water, and were divided into three subsamples for TEM, molecular (16S rRNA and metagenomic sequencing), and FISH-SEM experiments, respectively, following the protocol of Li et al. [4].

16S rRNA gene sequencing and correlative FISH-SEM analysis

The procedure used for PCR amplification, sequencing of 16S rRNA genes (~1,450 bp), and subsequent phylogenetic analysis are as described by [4]. 16S rRNA gene sequences that share identities > 98.7% (species criterion level) are regarded as from the same strain [5]. Bacterial identification was performed by a correlated FISH-SEM approach at the single-cell level [4]. Eight species-specific oligonucleotide probes were used for FISH experiments (Table S11). Their probe specificity was evaluated with the online probe evaluation tool ProbeMatch [6] (Table S12). Fluorescence microscopy experiments were carried out using an Olympus BX51 microscope. After fluorescence microscope observations, the same sample was carbon-coated using a Leica ACE200 Low Vacuum Sputter Coater (Leica Microsystems, Wetzlar, Germany), and was observed using a Zeiss Ultra-55 field-emission gun SEM (Carl Zeiss, Germany) operating at 5 kV.

Metagenome sequencing, scaffold assembly, and genome binning

To acquire sufficient DNA samples for whole-genome sequencing, the genome DNA of living MTB cells was amplified with a REPLI-g Single Cell Kit (Qiagen, Germany) following manufacturer instructions. Amplification products were then purified and small fragments (e.g., shorter than 4000 bp) were discarded with a QIAEX II Gel Extraction Kit (Qiagen, Germany). Purified genome DNA was sequenced using an Illumina HiSeq 6000 with the pair-end strategy of 150-bp reads with an average 270-bp insert size (Annoroad, Beijing, China). Raw Illumina reads were trimmed to remove adapter sequences and low-quality bases using the Trimmomatic software [7]. Then clean reads were assembled into scaffolds using the IDBA software [8] with kmer = 20, 40, 60, 80, 100, and 120, respectively, and optimal scaffolds assembled with different kmer values were filtered. Then optimal scaffolds were binned and reassembled with the MetaWRAP software [9]; scaffolds shorter than 1500 bp were abandoned. Completeness and contamination values of each genome were obtained using lineage-

specific marker genes and default parameters in CheckM v.1.0.12 [10]; high quality genomes were retained (completeness ≥ 80%, contamination ≤ 5%). Via BLAST search, nine 16S rRNA gene fragments were identified from genome drafts and were linked to decided MTB strains by the correlative FISH-SEM approach (Table S4). The other six genomes also correspond to MTB strains because they were retrieved from phylogenetically and morphologically identified MTB. The average nucleotide identity (ANI) was calculated using the Jspecies software (version 1.2.1) [11].

Gene annotation and phylogenetic analysis

Genomes were annotated with the online database GeneMarkS (http://topaz.gatech.edu/GeneMark/genemarks.cgi) [12]. Homologous magnetosome protein sequences Mam-A, -B, E, -I, -K, -M, -P, and -Q were identified within the refseq_protein database using the offline BLAST software with each magnetosome protein from *Magnetospirillum gryphiswaldense* MSR-1, *Magnetococcus marinus* MC-1, *Desulfovibrio magneticus* RS-1, and *Ca.* Magnetobacterium casensis MYR-1 as query sequence. Proteins within the threshold value (i.e., identification ≥ 50%, coverage ≥ 90%) are regard as homologous proteins. Other magnetosome proteins and proteins in MGCs were checked manually and verified using the NCBI BLAST webserver [13]. The whole-genome phylogenetic tree was constructed using the genome data acquired here and from the NCBI database (www.ncbi.nlm.nih.gov). The GTDB-Tk v.0.1.3 'classify_wf' command was used to find 120 single-copy bacterial marker protein sequences from these genomes to construct their multiple alignments and taxonomic assignments using the GTDB r86 database [14]. A maximum-likelihood tree was calculated with IQ-TREE [15] under evolutionary models selected by ModelFinder [16] with 1000 ultrafast bootstraps. Finally, the whole-genome tree was visualized using FigTree v1.4.2 (http://tree.bio.ed.ac.uk/software/figtree/). Both the 16S rRNA gene sequences of MTB strains and the coding protein sequences of magnetosome genes containing HtrA protease-like domain (i.e., *mamO*, *mamE*, *mamE-Cter*, *mamEO*, and *mamE-Nter*) were aligned using the Muscle algorithm (version 3.8.31) [17]. Phylogenetic trees based on 16S rRNA gene sequences and coding protein sequences were both constructed using the same method as stated above.

Conserved protein domain prediction, and 3-D protein structure modeling

Protein transmembrane domains are predicted using the TMHMM 2.0 online server (https://services.healthtech.dtu.dk/service.php?TMHMM-2.0). Functional protein domains were analyzed using the CDD database [18]. The 3-D protein structure models were constructed using the DeepMind AlphaFold2 [19] and RoseTTAFold [20] software. Predicted local-distance difference test (pLDDT) scores were used to assess confidence measures. After comparing predicted protein structures from the two software packages, protein structure models with lower confidence (pLDDT) were

discarded. The 3-D protein structure comparison was performed using the PyMOL software (version 2.4). The root mean squared deviation (RMSD) was used to evaluate the structural similarities of proteins.

TEM Analysis

TEM experiments were performed with a JEM2100 instrument (JEOL Ltd., Tokyo, Japan) operating at 200 kV at the Institute of Geology and Geophysics, Chinese Academy of Sciences, Beijing, China. Cell diameter, particle number, and crystal length (along the long axis) and width (perpendicular to the long axis) of magnetite particles were measured from TEM images of individual MTB cells. The shape factor of particles is the width/length ratio. For each MTB strain, at least 30 individual cells were selected randomly for statistical analysis of cell diameter and particle number, with at least 300 individual particles selected randomly for statistical analysis of crystal length and width.

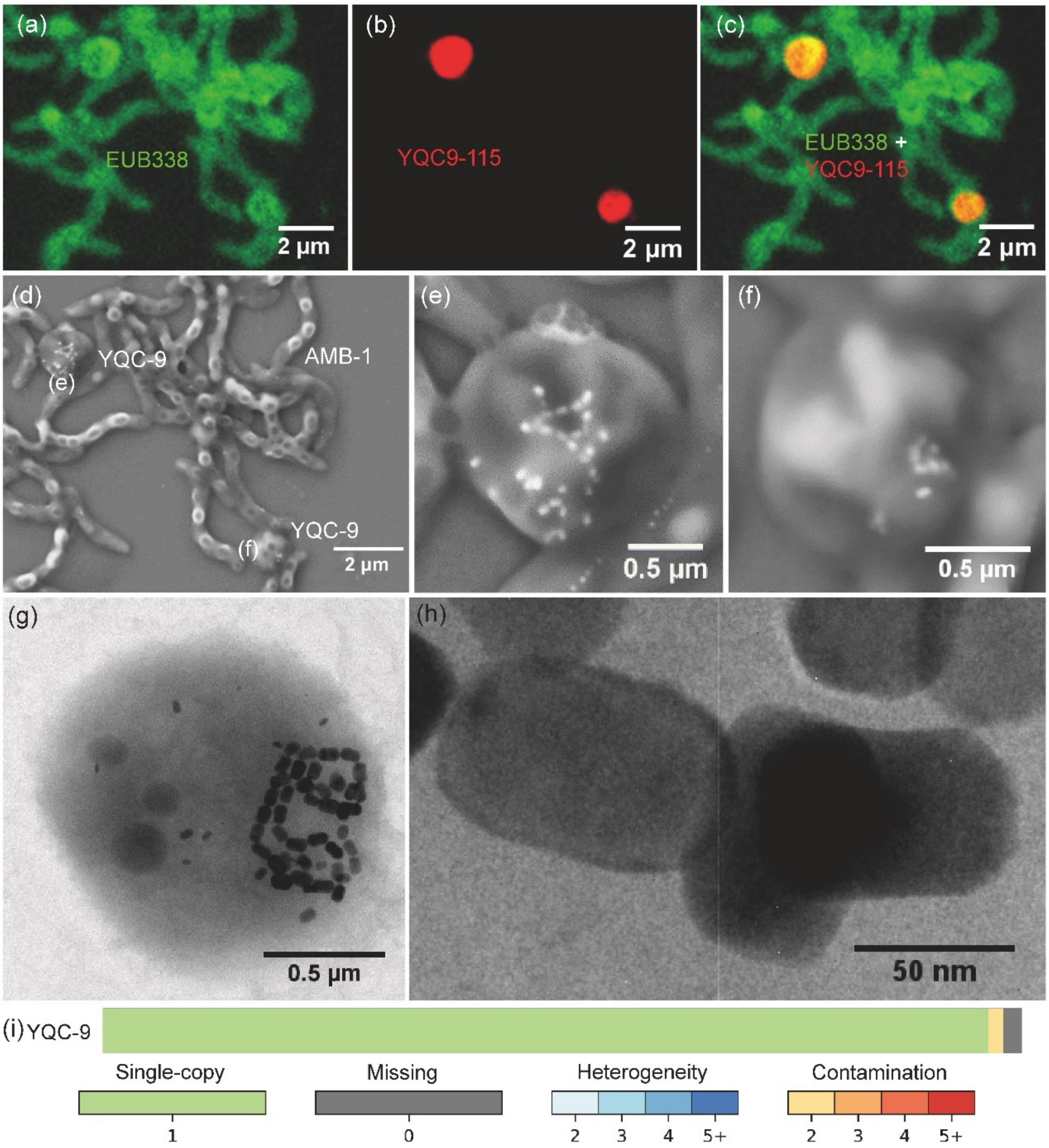

Figure S1. Bacterial identification, morphological, and genomic features of strain YQC-9. (a) Fluorescence microscopy image of YQC-9 cells *in situ* hybridized with the 5′-FAM-labeled universal bacterial probe EUB338. (b) Fluorescence microscopy image of YQC-9 cells hybridized *in situ* with the 5′-Cy3-labeled YQC-9-specific probe YQC9-115. (c) Overlapping fluorescence microscopy image of (a) and (b). (d) Low-magnification SEM image of the same field of view as in (c). (e) and (f) SEM images of two YQC-9 cells indicated by corresponding lowercase letters in (d). These cells contain non-linear magnetosome chains. (g) TEM image of YQC-9. (h) High-magnification TEM image of magnetic particles in a YQC-9 cell. (i) The bar chart represents the completeness, contamination, and strain heterogeneity of the YQC-9 genome.

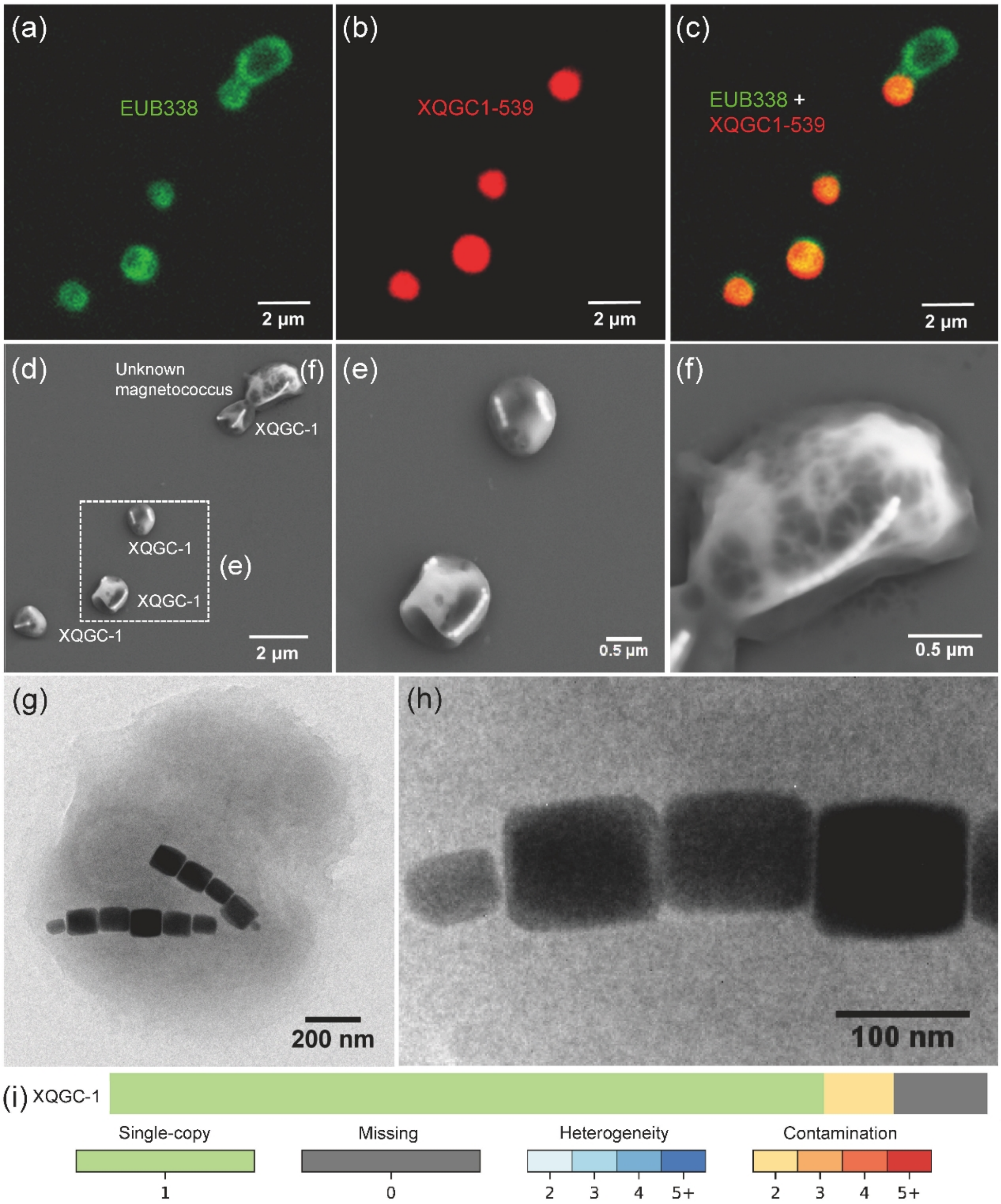

Figure S2. Bacterial identification, morphological, and genomic features of strain XQGC-1. (a) Fluorescence microscopy image of XQGC-1 cells *in situ* hybridized with the 5′-FAM-labeled universal bacterial probe EUB338. (b) Fluorescence microscopy image of XQGC-1 cells hybridized *in situ* with the 5′-Cy3-labeled XQGC-1-specific probe XQGC1-539. (c) Overlapping fluorescence microscopy image of (a) and (b). (d) SEM image of the same field of view as in (c). (e) High-magnification SEM image for a region with two XQGC-1 cells indicated by the white dashed square in (d). Both cells of strain XQGC-1 contain two magnetosome chains. (f) High-magnification SEM image of an unknown MTB cell indicated by the corresponding lowercase letter in (d). This MTB cell contains one magnetosome chain. (g) TEM image of a XQGC-1 cell. (h) High-magnification TEM image of magnetic particles in XQGC-1. (i) The bar chart represents the completeness, contamination, and strain heterogeneity of the XQGC-1 genome.

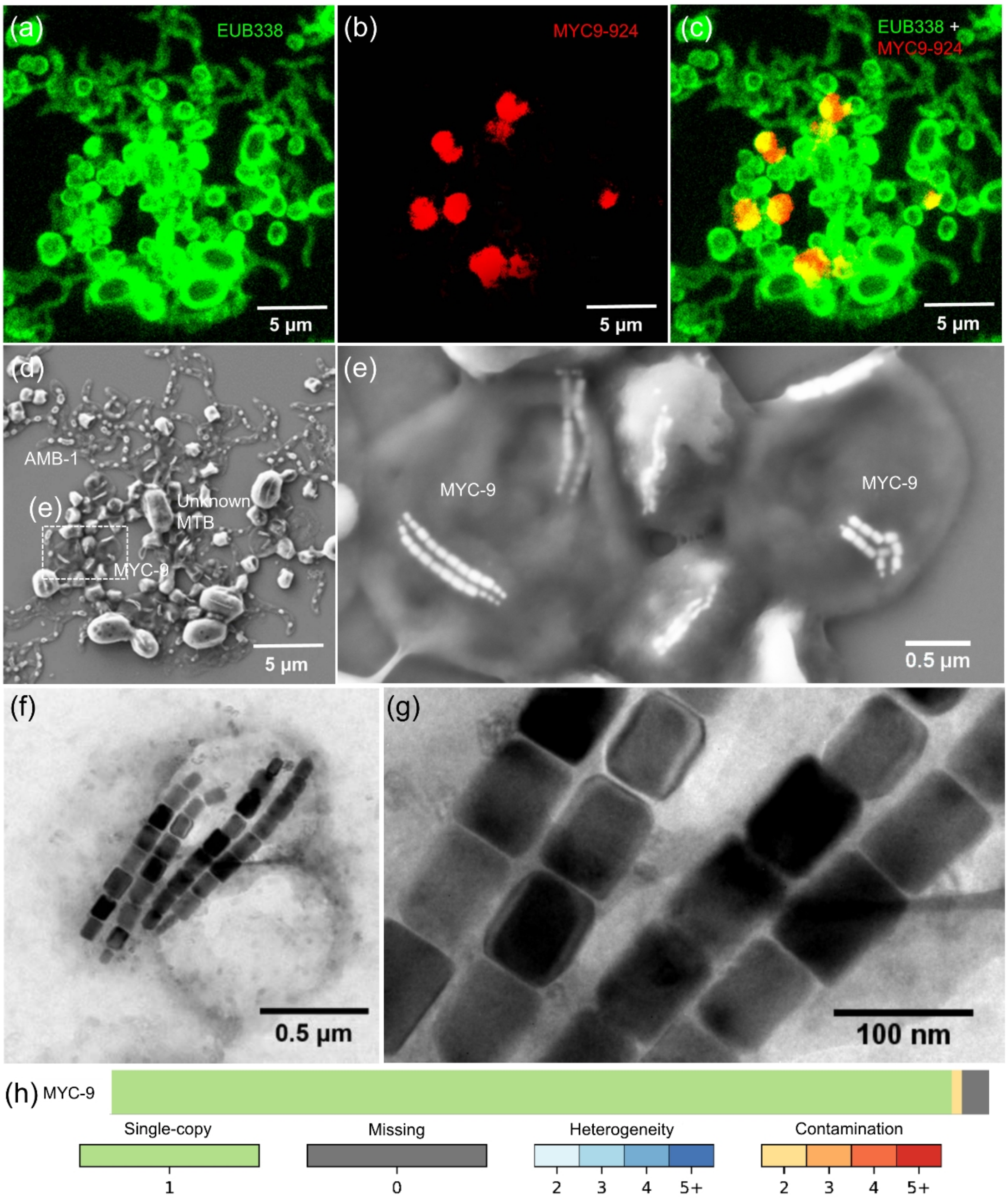

Figure S3. Bacterial identification, morphological, and genomic features of strain MYC-9. (a) Fluorescence microscopy image of MYC-9 cells *in situ* hybridized with the 5′-FAM-labeled universal bacterial probe EUB338. (b) Fluorescence microscopy image of MYC-9 cells hybridized *in situ* with the 5′-Cy3-labeled MYC-9-specific probe MYC9-924. (c) Overlapping fluorescence microscopy image of (a) and (b). (d) SEM image of the same field of view as in (c). (e) High-magnification SEM image for a region with two MYC-9 cells outlined by the white dashed box in (d). Both cells of strain MYC-9 contain two double magnetosome chains. (f) TEM image of a MYC-9 cell. (g) High-magnification TEM image of magnetic particles in MYC-9. (h) The bar chart represents the completeness, contamination, and strain heterogeneity of the MYC-9 genome.

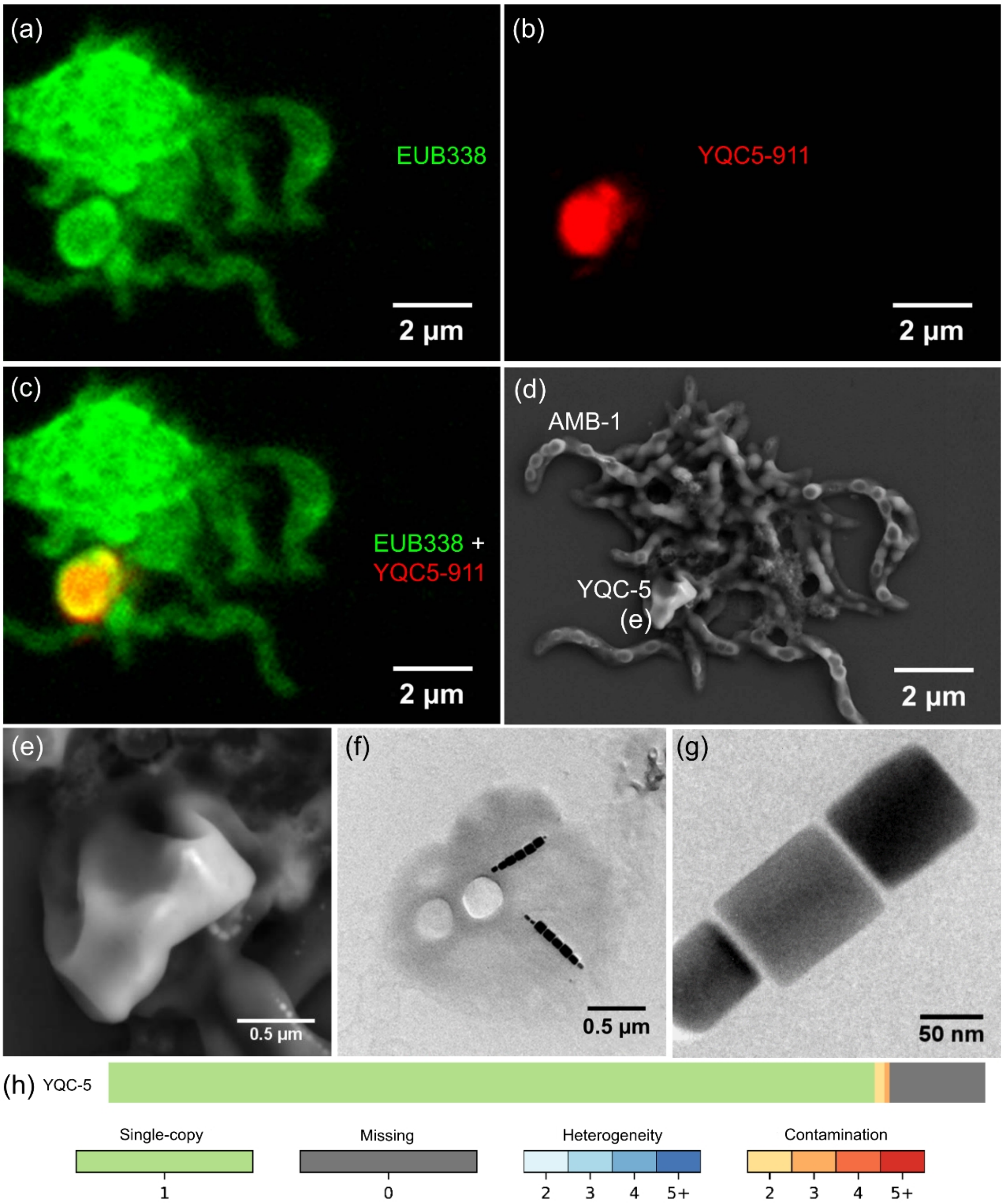

Figure S4. Bacterial identification, morphological, and genomic features of strain YQC-5. (a) Fluorescence microscopy image of a YQC-5 cell *in situ* hybridized with the 5′-FAM-labeled universal bacterial probe EUB338. (b) Fluorescence microscopy image of a YQC-5 cell hybridized *in situ* with the 5′-Cy3-labeled YQC-5-specific probe YQC5-911. (c) Overlapping fluorescence microscopy image of (a) and (b). (d) SEM image of the same field of view as in (c). (e) SEM image of a YQC-5 cell indicated by corresponding lowercase letters in (d). This cell contains double magnetosome chains. (f) TEM image of a YQC-5 cell. (g) High-magnification TEM image of magnetic particles in YQC-5. (h) The bar chart represents the completeness, contamination, and strain heterogeneity of the YQC-5 genome.

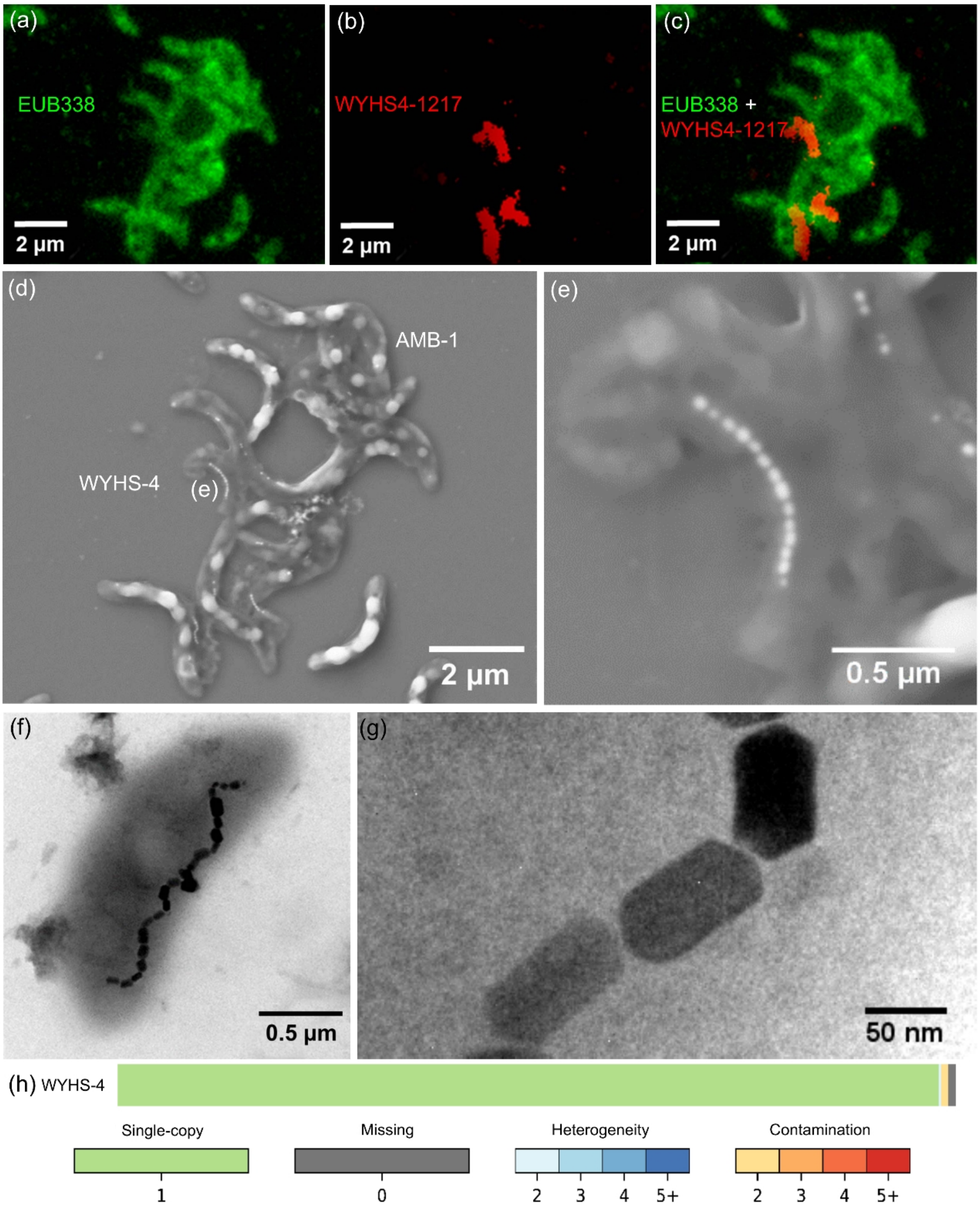

Figure S5. Bacterial identification, morphological, and genomic features of strain WYHS-4. (a) Fluorescence microscopy image of WYHS-4 cells *in situ* hybridized with the 5′-FAM-labeled universal bacterial probe EUB338. (b) Fluorescence microscopy image of WYHS-4 cells hybridized *in situ* with the 5′-Cy3-labeled WYHS-4-specific probe WYHS4-1217. (c) Overlapping fluorescence microscopy image of (a) and (b). (d) Low-magnification SEM image of the same field of view as in (c). (e) SEM image of a WYHS-4 cell indicated by the corresponding lowercase letter in (d). The WYHS-4 cell contains a single magnetosome chain. (f) TEM image of a WYHS-4 cell. (g) High-magnification TEM image of magnetic particles in a WYHS-4 cell. (h) The bar chart represents the completeness, contamination, and strain heterogeneity of the WYHS-4 genome.

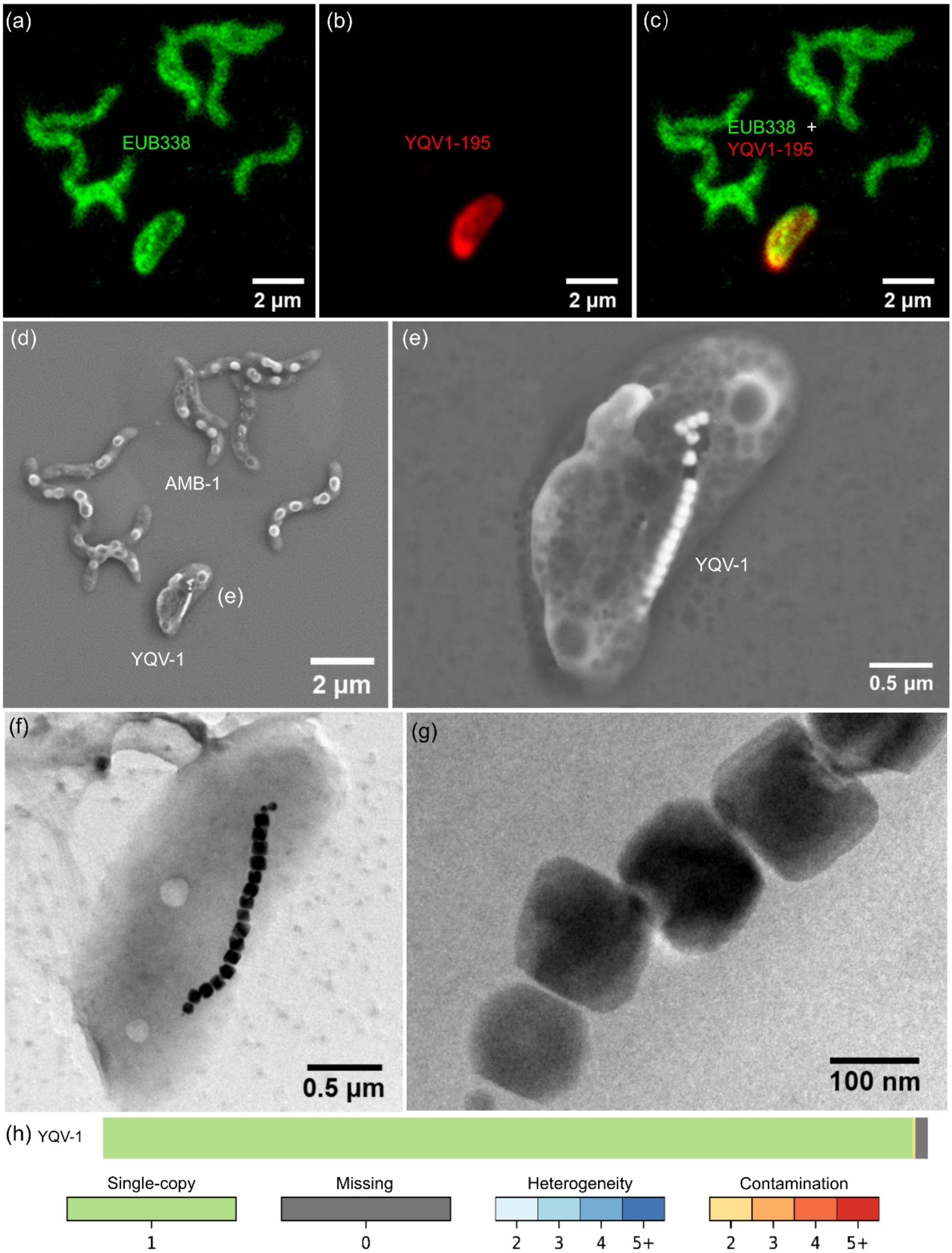

Figure S6. Bacterial identification, morphological, and genomic features of strain YQV-1. (a) Fluorescence microscopy image of a YQV-1 cell *in situ* hybridized with the 5′-FAM-labeled universal bacterial probe EUB338. (b) Fluorescence microscopy image of a YQV-1 cell hybridized *in situ* with the 5′-Cy3-labeled YQV-1-specific probe YQV1-195. (c) Overlapping fluorescence microscopy image of (a) and (b). (d) Low-magnification SEM image of the same field of view as in the boxed area in (c). (e) SEM image of a YQV-1 cell indicated by the corresponding lowercase letter in (d). The YQV-1 cell contains a single magnetosome chain. (f) TEM image of a YQV-1 cell. (g) High-magnification TEM image of magnetic particles in YQV-1. (h) The bar chart represents the completeness, contamination, and strain heterogeneity of the YQV-1 genome.

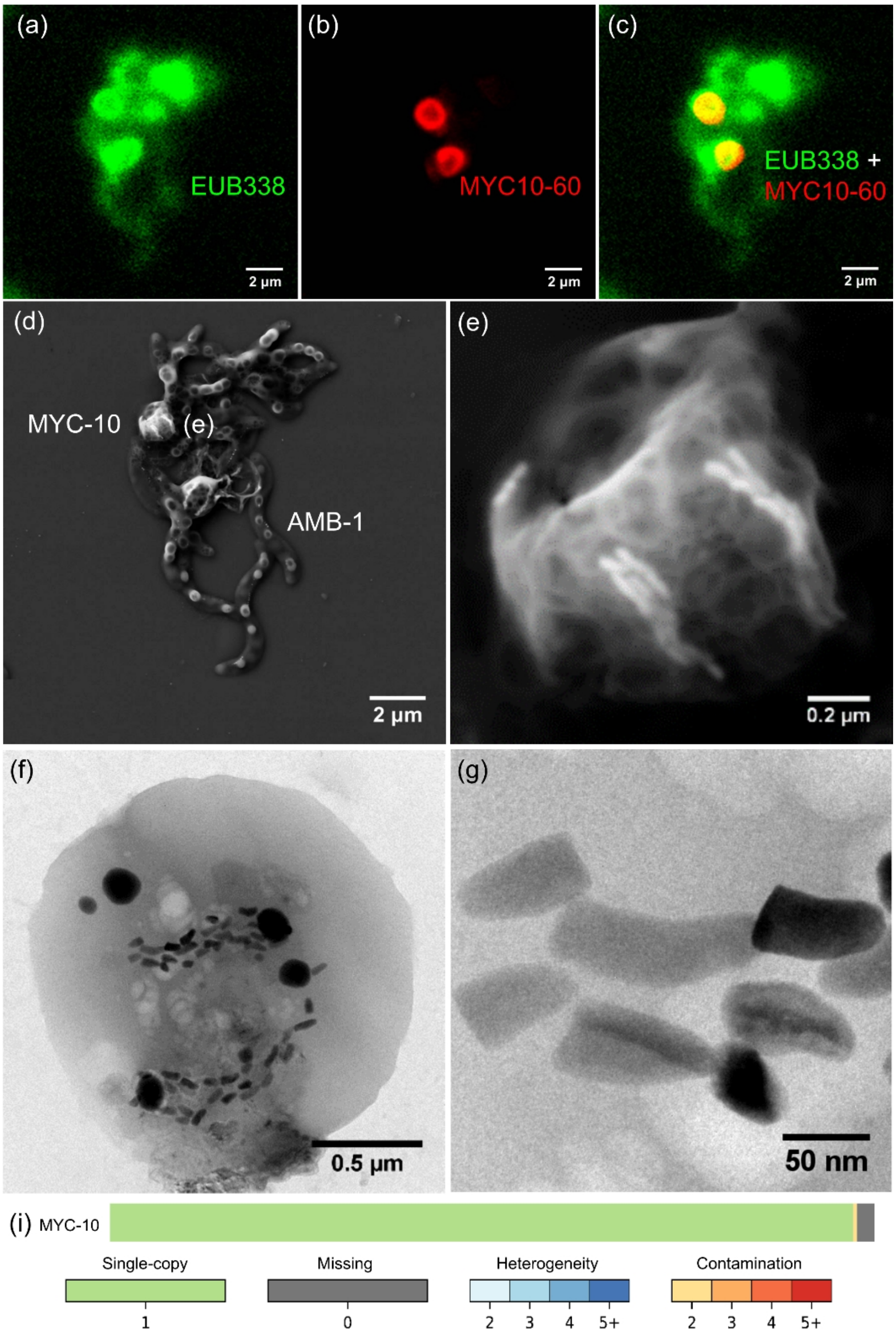

Figure S7. Bacterial identification, morphological, and genomic features of strain MYC-10. (a) Fluorescence microscopy image of MYC-10 cells *in situ* hybridized with the 5′-FAM-labeled universal bacterial probe EUB338. (b) Fluorescence microscopy image of MYC-10 cells hybridized *in situ* with the 5′-Cy3-labeled MYC-10-specific probe MYC10-60. (c) Overlapping fluorescence microscopy image of (a) and (b). (d) SEM image of the same field of view as in (c). (e) SEM image of a MYC-10 cell indicated by the corresponding lowercase letter in (d). The MYC-10 cell contains multiple magnetosome chain bundles. (f) TEM image of a MYC-10 cell. (g) High-magnification TEM image of magnetic particles in MYC-10. (h) The bar chart represents the completeness, contamination, and strain heterogeneity of the MYC-10 genome.

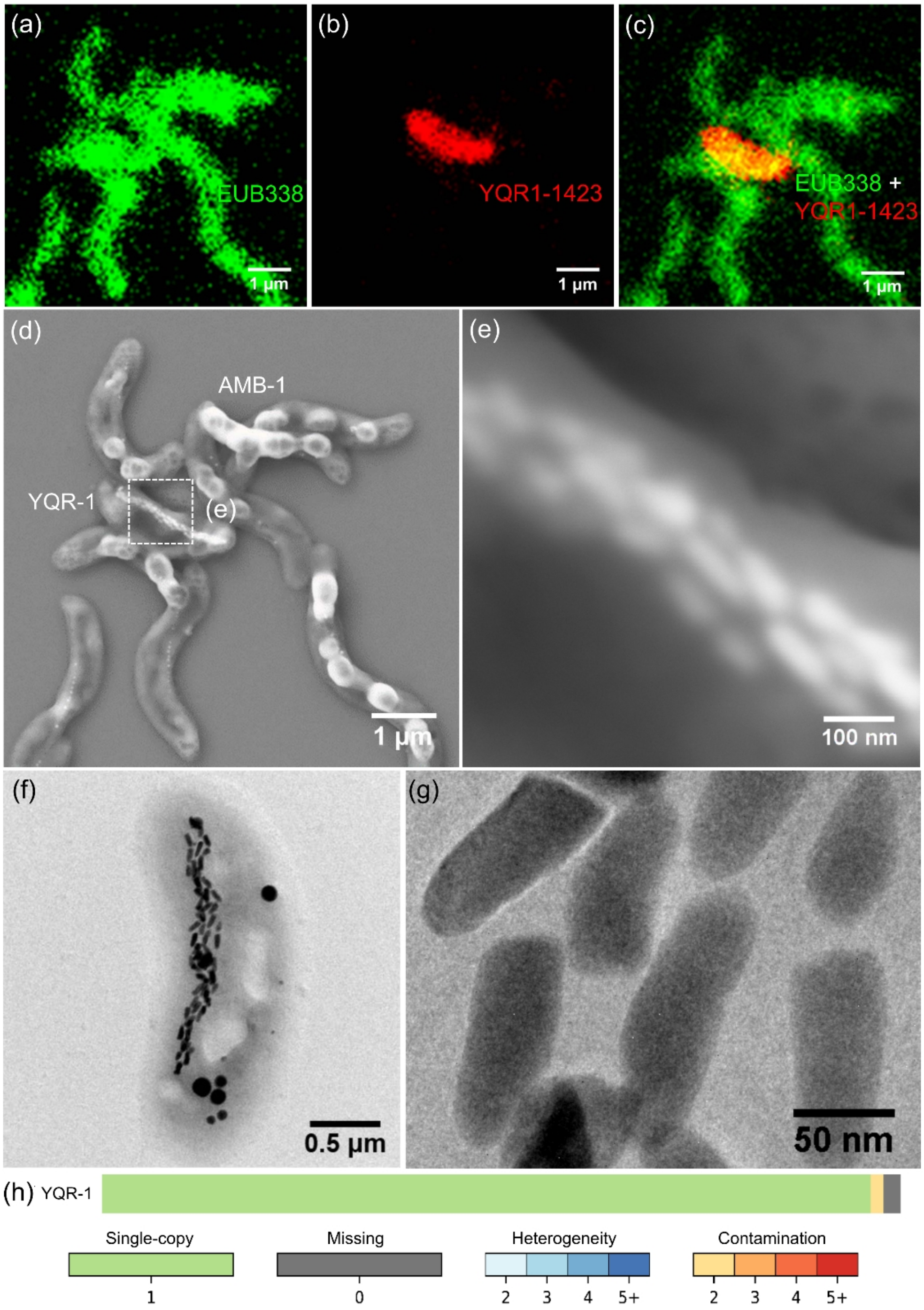

Figure S8. Bacterial identification, morphological, and genomic features of strain YQR-1. (a) Fluorescence microscopy image of a YQR-1 cell *in situ* hybridized with the 5′-FAM-labeled universal bacterial probe EUB338. (b) Fluorescence microscopy image of a YQR-1 cell hybridized *in situ* with the 5′-Cy3-labeled YQR-1-specific probe YQR1-1423. (c) Overlapping fluorescence microscopy image of (a) and (b). (d) SEM image of the same field of view as in (c). (e) High-magnification SEM image of YQR-1 magnetic particles outlined by the white dashed box in (d). (f) TEM image of a YQR-1 cell. (g) High-magnification TEM image of magnetic particles in YQR-1. (h) The bar chart represents the completeness, contamination, and strain heterogeneity of the YQR-1 genome.

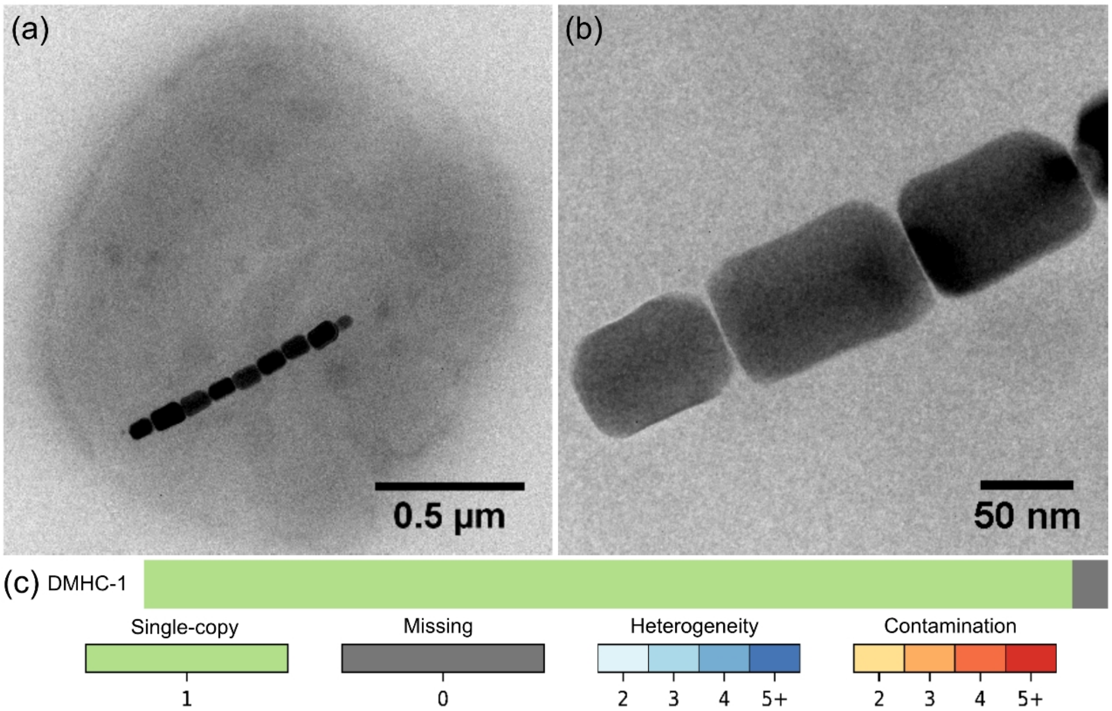

Figure S9. Morphological and genomic features of strain DMHC-1. (a) TEM image of a DMHC-1 cell. (b) High-magnification TEM image of magnetic particles in DMHC-1. (c) The bar chart represents the completeness, contamination, and strain heterogeneity of the DMHC-1 genome.

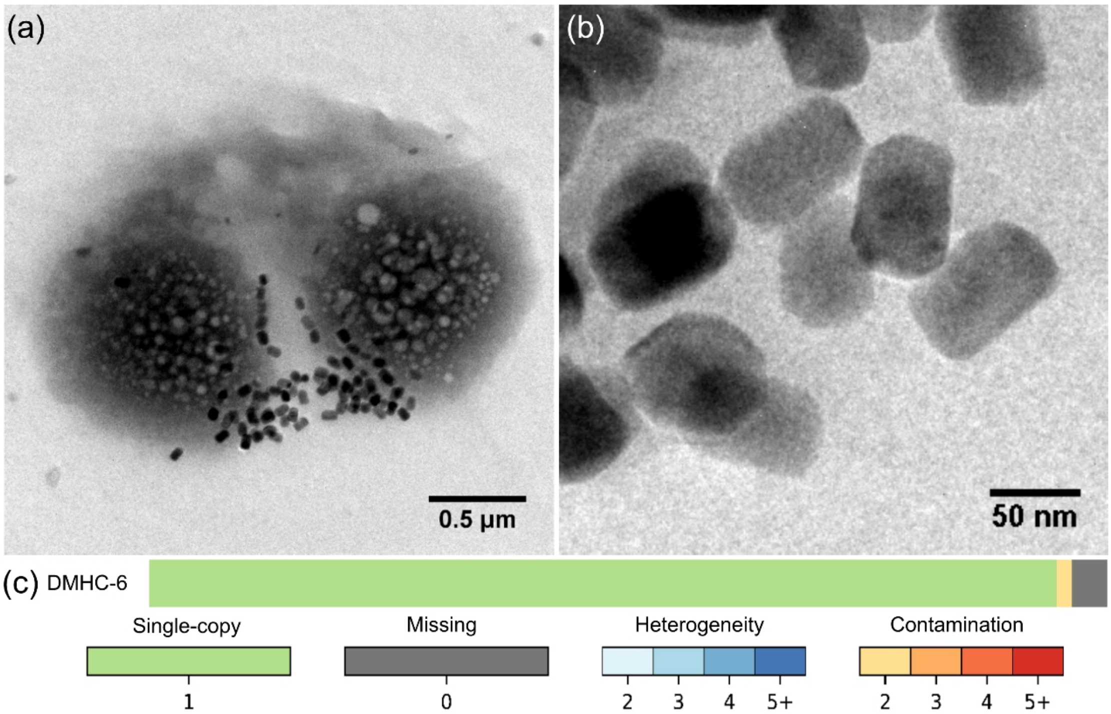

Figure S10. Morphological and genomic features of strain DMHC-6. (a) TEM image of a DMHC-6 cell. (b) High-magnification TEM image of magnetosome assembly configurations of DMHC-6. (c) Bar chart represents the completeness, contamination, and strain heterogeneity of the DMHC-6 genome.

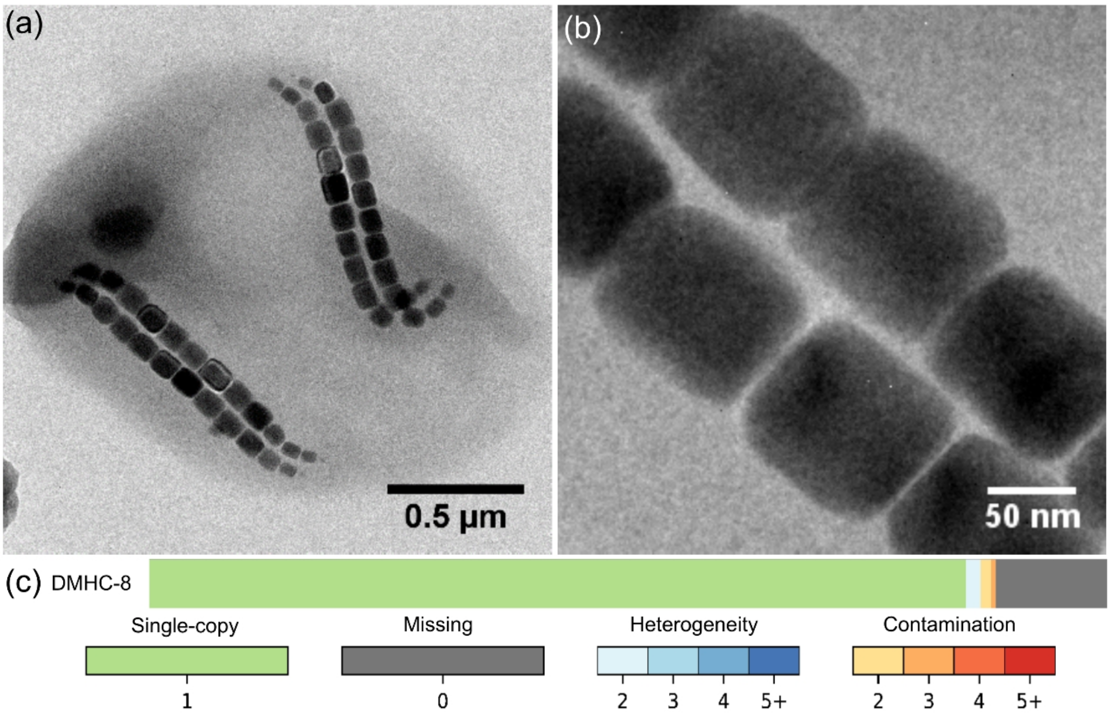

Figure S11. Morphological and genomic features of strain DMHC-8. (a) TEM image of a DMHC-8 cell. (b) High-magnification TEM image of magnetic particles in DMHC-8. (c) The bar chart represents the completeness, contamination, and strain heterogeneity of the DMHC-8 genome.

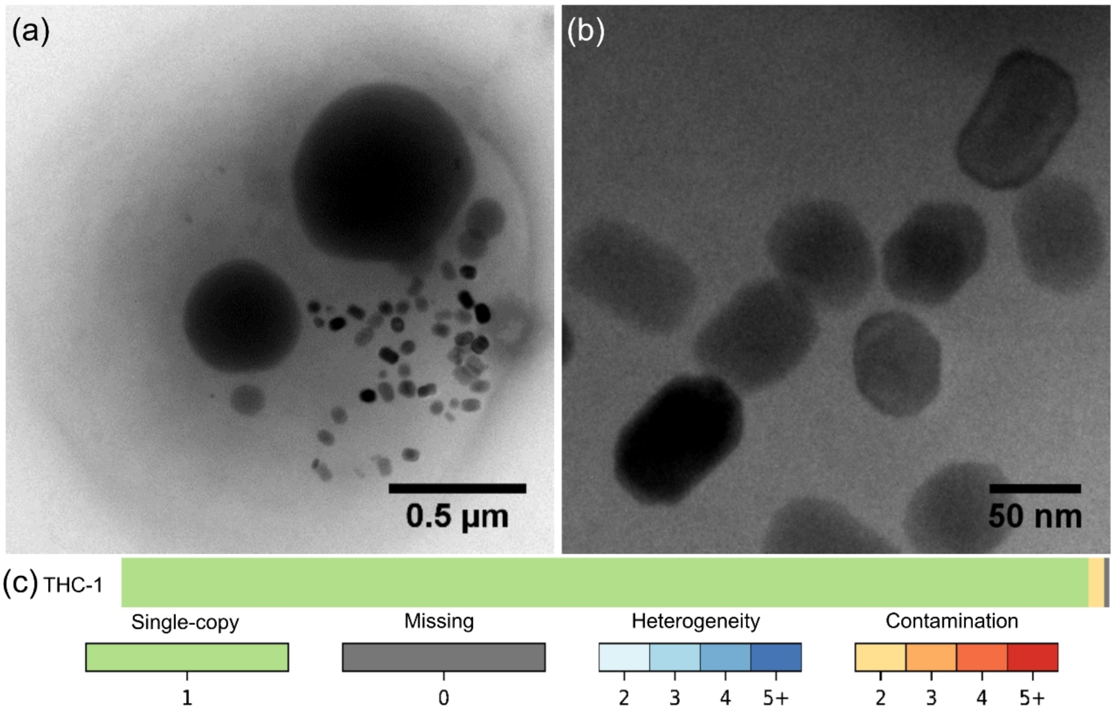

Figure S12. Morphological and genomic features of strain THC-1. (a) TEM image of a THC-1 cell. (b) High-magnification TEM image of magnetic particles in THC-1. (c) The bar chart represents the completeness, contamination, and strain heterogeneity of the THC-1 genome.

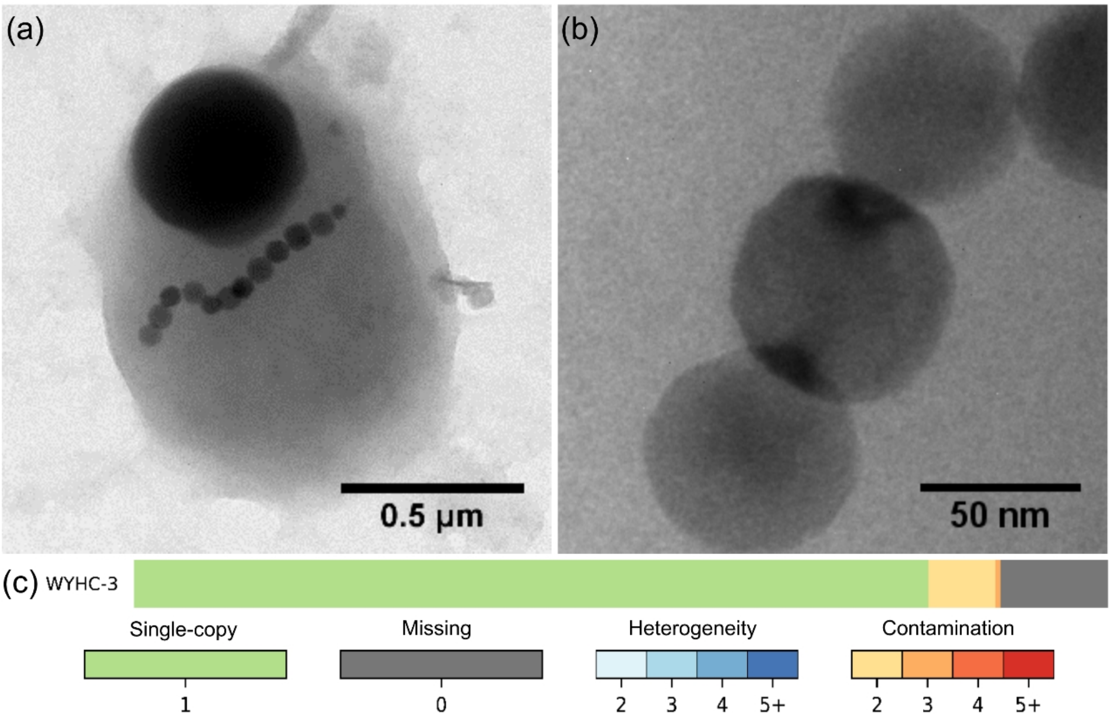

Figure S13. Morphological and genomic features of strain WYHC-3. (a) TEM image of a WYHC-3 cell. (b) High-magnification TEM image of magnetic particles in WYHC-3. (c) The bar chart represents the completeness, contamination, and strain heterogeneity of the WYHC-3 genome.

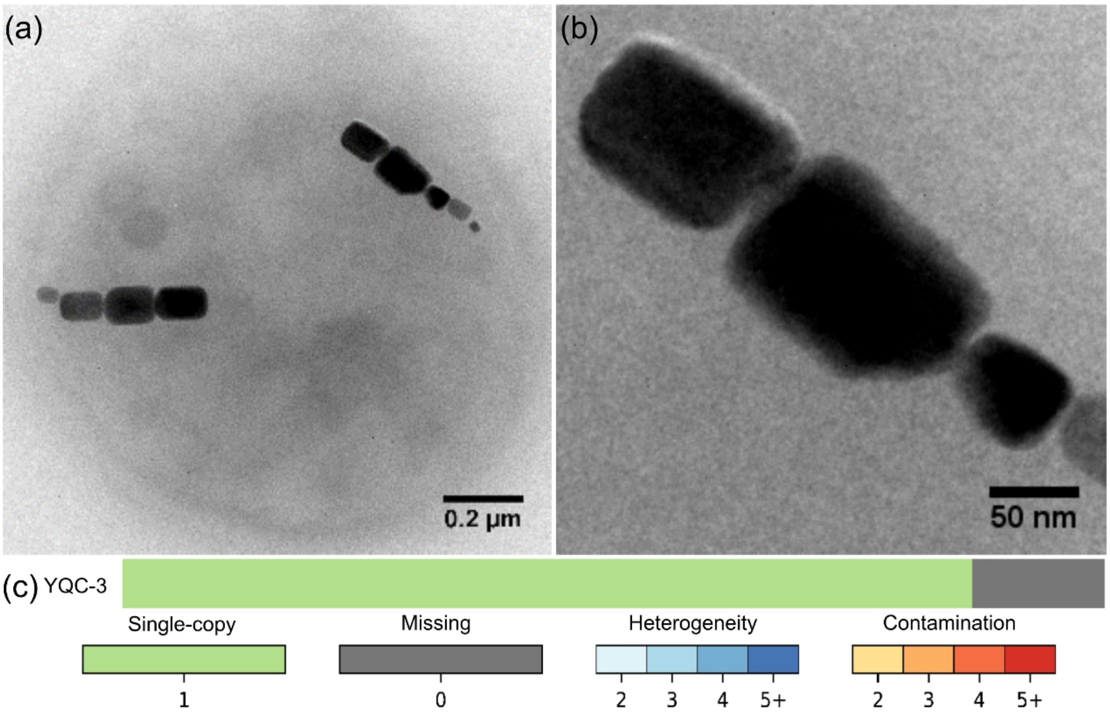

Figure S14. Morphological and genomic features of strain YQC-3. (a) TEM image of a YQC-3 cell. (b) High-magnification TEM image of magnetic particles in strain YQC-3. (c) The bar chart represents the completeness, contamination, and strain heterogeneity of the YQC-3 genome.

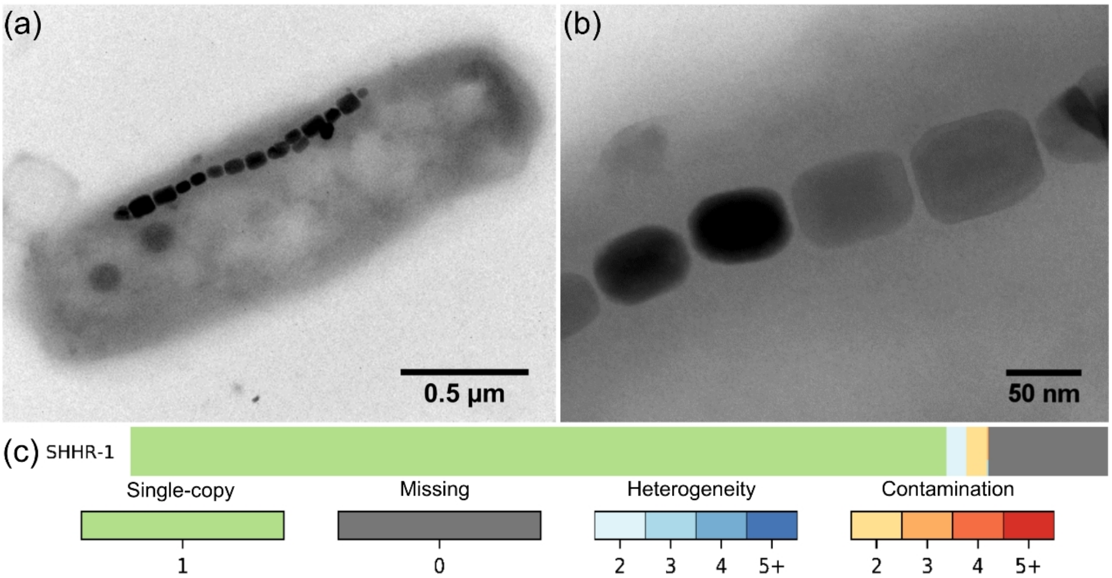

Figure S15. Morphological and genomic features of strain SHHR-1. (a) TEM image of the SHHR-1 cell. (b) High-magnification TEM image of magnetic particles in strain SHHR-1. (c) The bar chart represents the completeness, contamination, and strain heterogeneity of the SHHR-1 genome.

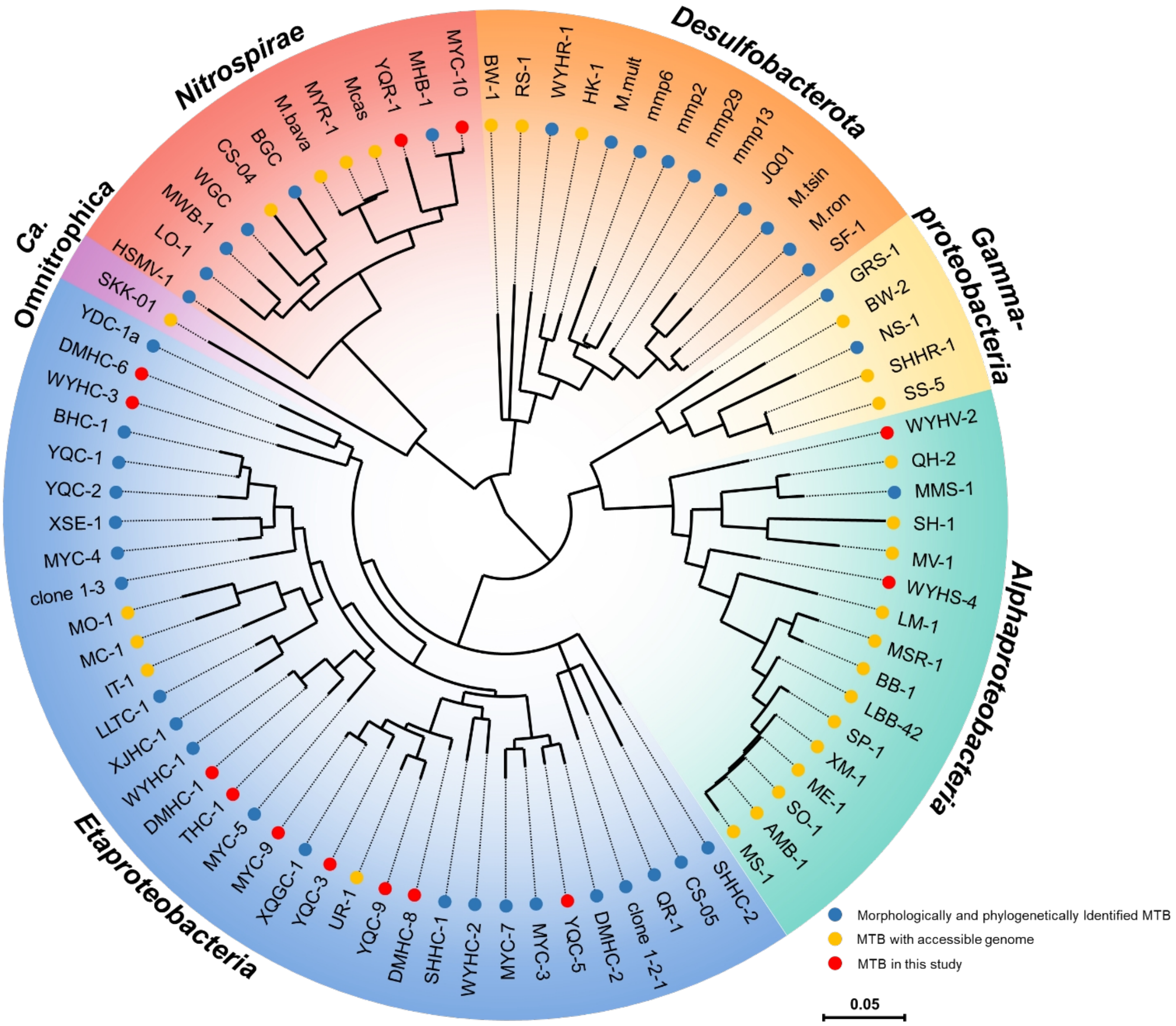

Figure S16. Phylogenetic distribution of phylogenetically and morphologically identified MTB (blue solid circles), MTB with accessible genome (yellow solid circles), and MTB in this study (red solid circles). The phylogenetic tree was constructed based on 16S rRNA gene sequences. Bootstrap values at nodes are percentages of 1,000 replicates.

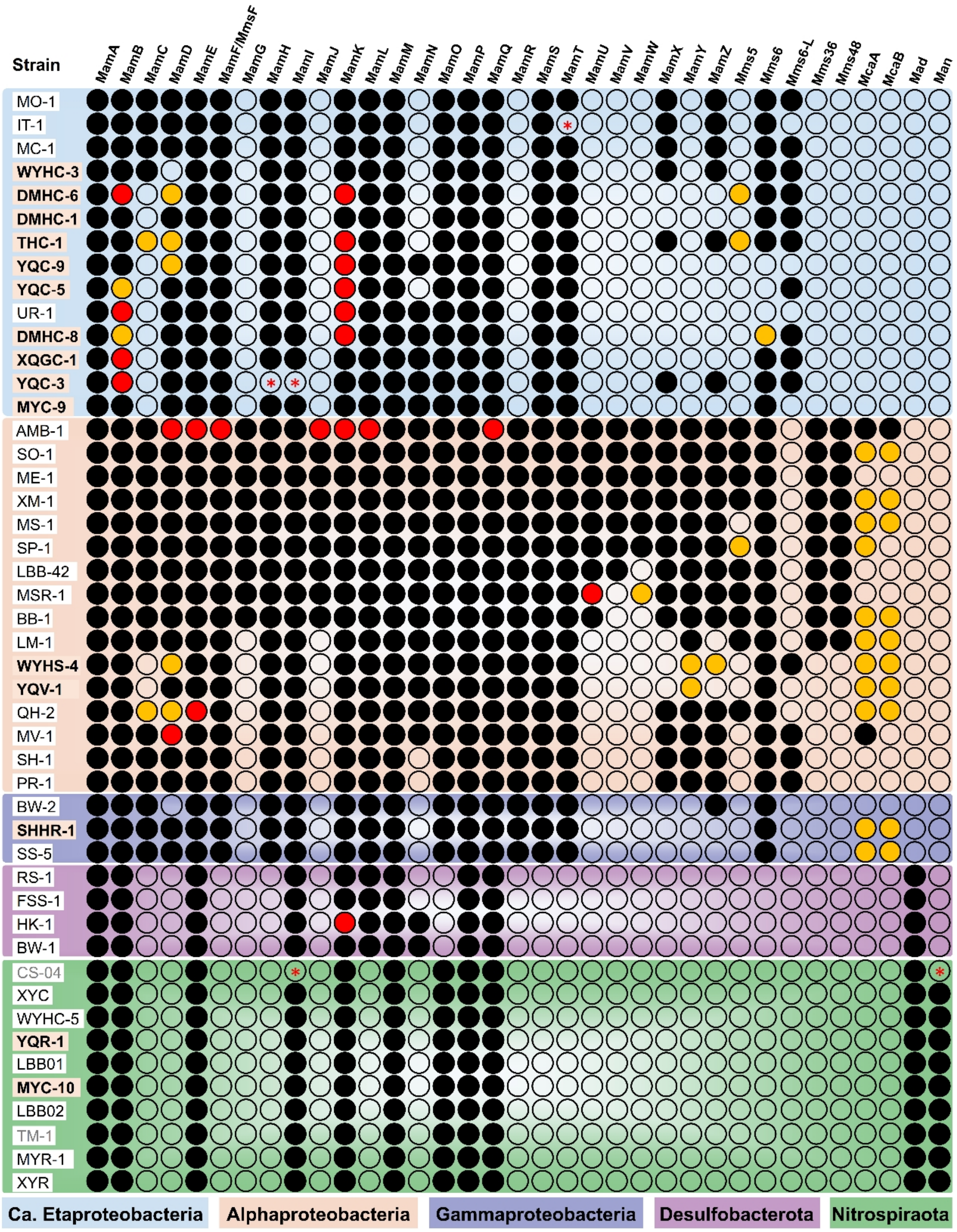

Figure S17. Magnetosome gene content in 47 tested MTB strains. MTB strains are shown in groups according to their phylogeny. Strain names in bold and yellow background, white background, and gray font represent genomes reported in this study, reported previously, and containing high contamination (> 10%), respectively. Black solid circles indicate that the gene was identified in MGCs. Yellow solid circles indicate that the gene was identified outside MGCs. Red solid circles indicate that the gene was identified both within and outside MGCs. Black hollow circles indicate that the gene is absent in genomes. Black hollow circles with a red asterisk inside indicate that the gene was not detected possibly due to incomplete genome sequencing. For instance, the absence of *mamI* in YQC-3 is possibly due to incomplete gene sequencing (~80.2% completeness).

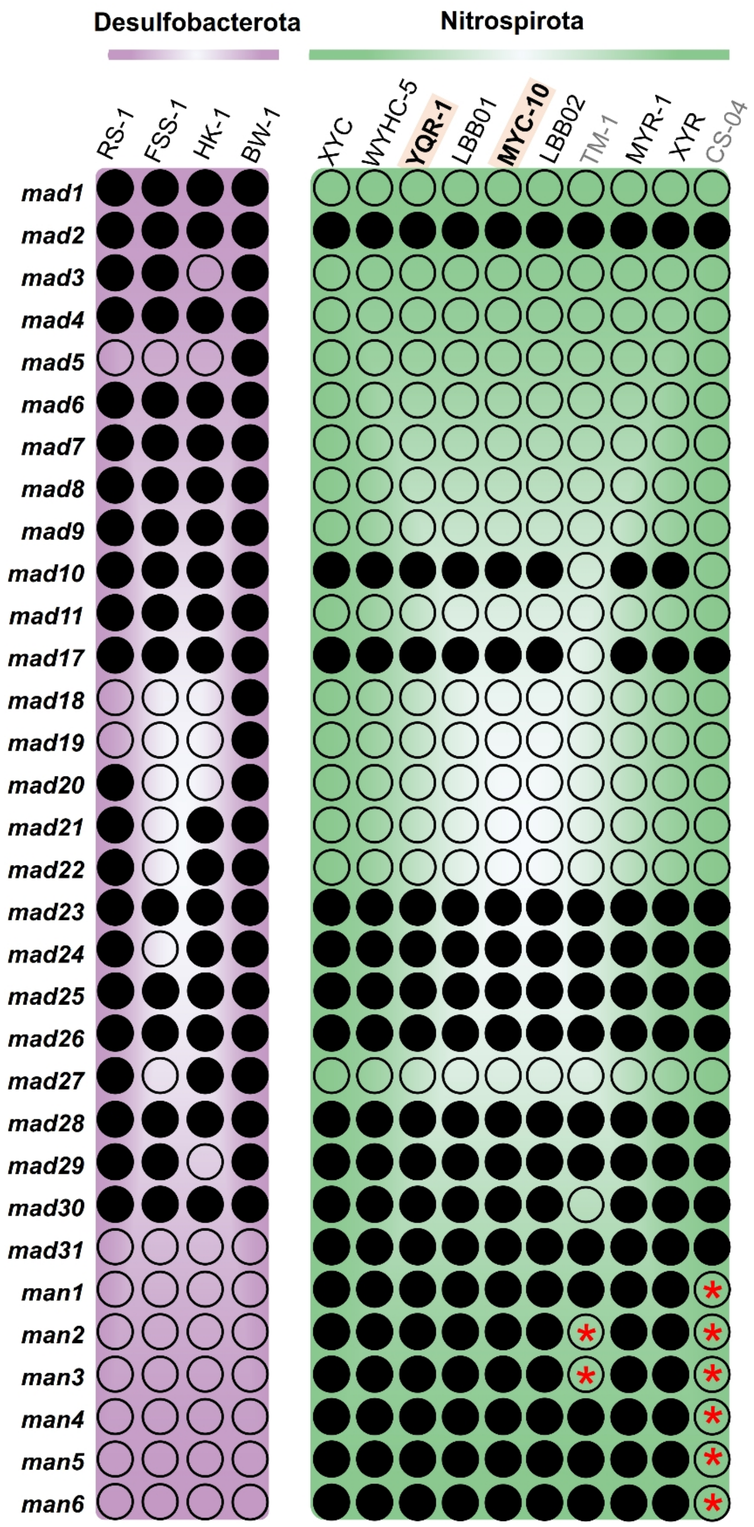

Figure S18. Content of *mad* and *man* genes in MTB strains affiliated with the Desulfobacterota and Nitrospirota phyla. MTB strains are shown in groups according to their phylogeny. Strain names in bold and yellow background represent genomes from this study; those in gray font have high contamination (> 10%). Black solid circles indicate that the gene was discovered in genomes. Black hollow circles indicate that the gene was absent in genomes. Black hollow circles with a red asterisk inside indicate that the gene was not detected possibly due to incomplete genome sequencing. The absence of some *man* genes in the genome of *Candidatus* Magnetobacterium bavaricum (~87.1% completeness) and strain CS-04 (~81.6% completeness) are possibly due to incomplete gene sequencing.

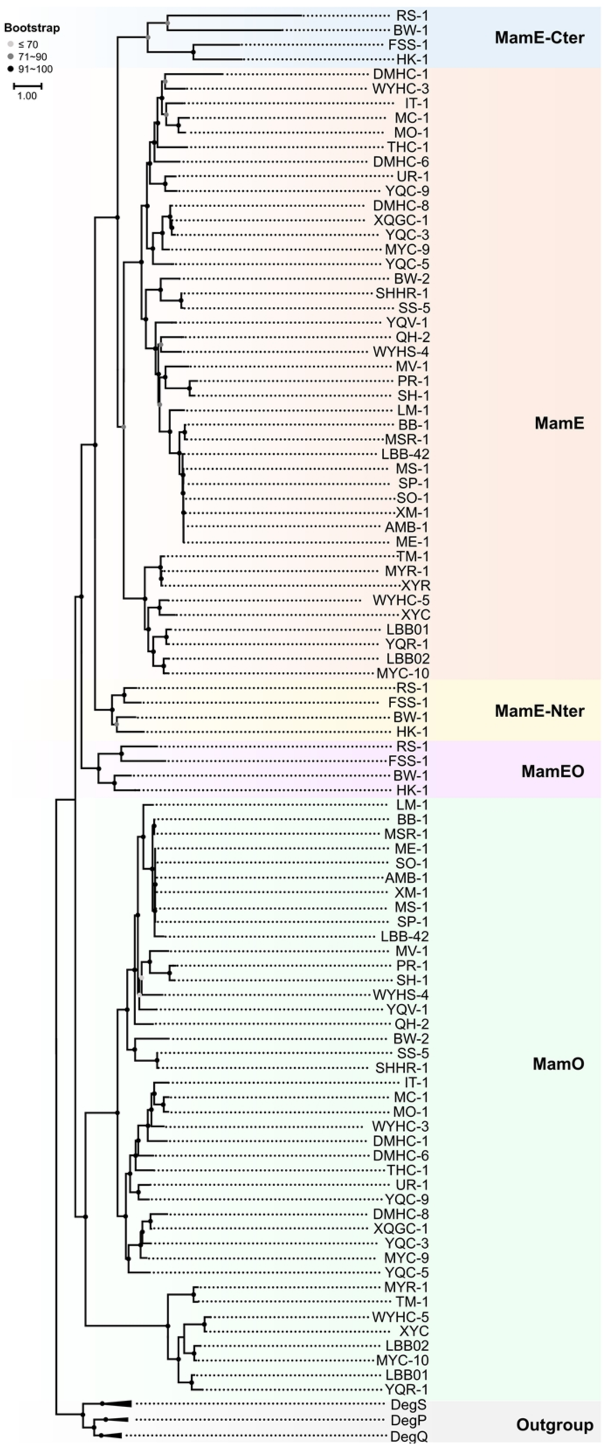

Figure S19. Phylogenetic distribution of magnetosome genes containing HtrA protease-like domain (i.e., *mamO*, *mamE*, *mamE-Cter*, *mamEO*, and *mamE-Nter*). The phylogenetic tree is based on the coding protein sequences of the magnetosome genes. The HtrA protease family proteins DegS, DegP, and DegQ from strains in the Pseudomonadota phylum were used to root the tree. Bootstrap values at nodes are percentages of 1,000 replicates.

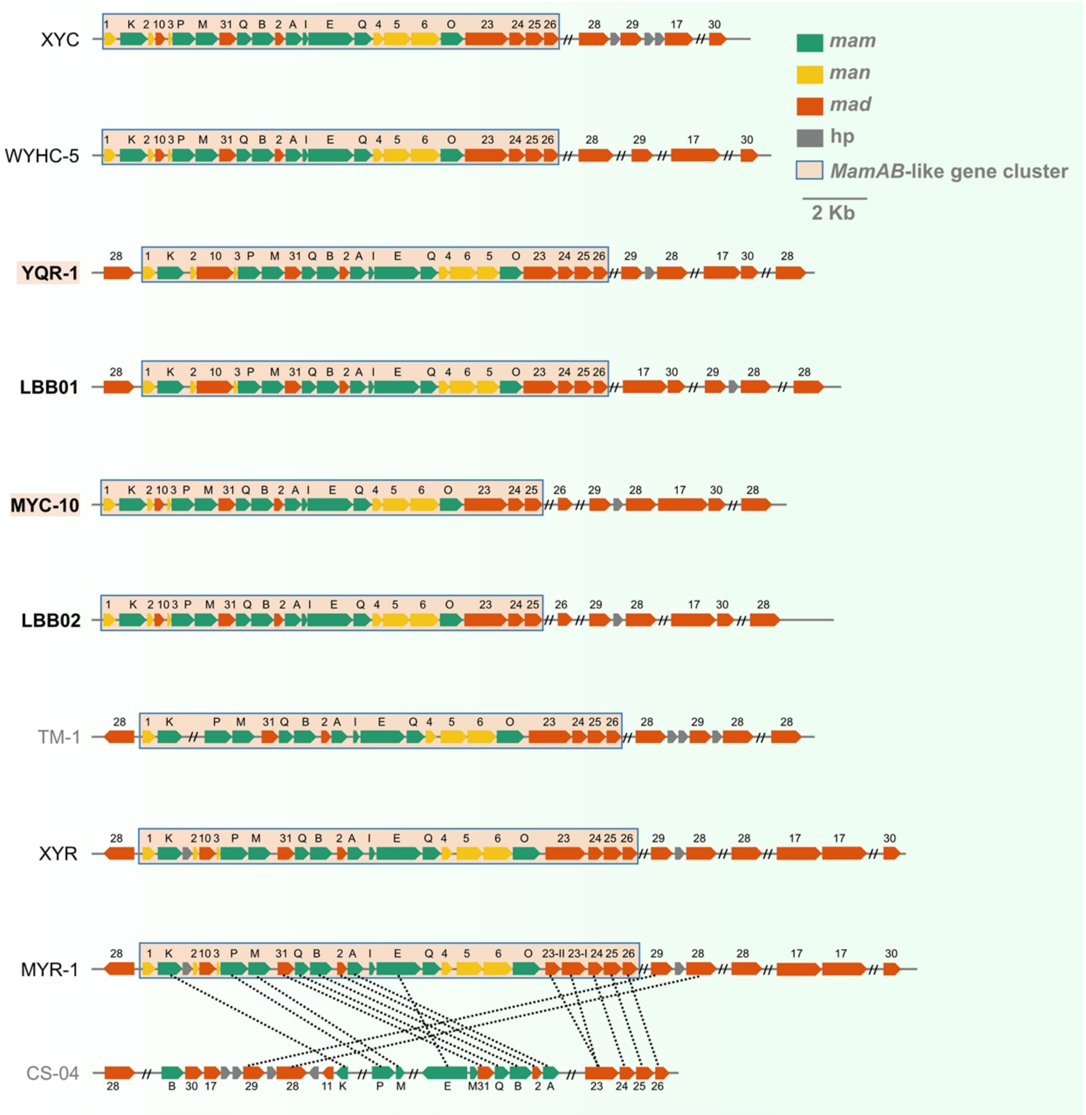

Figure S20. Gene organization and order of MGCs in MTB strains affiliated with the Nitrospirota phylum. Strain names in bold and yellow background represent genomes reported in this study; those in gray font have high contamination (> 10%). Dotted lines between MGCs indicate homologous genes.

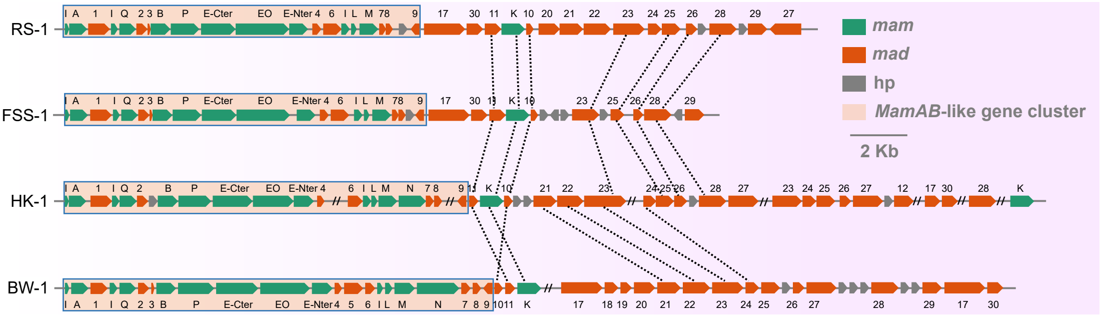

Figure S21. Gene organization and order of MGCs in MTB strains affiliated with the Desulfobacterota phylum. Dotted lines between MGCs indicate homologous genes.

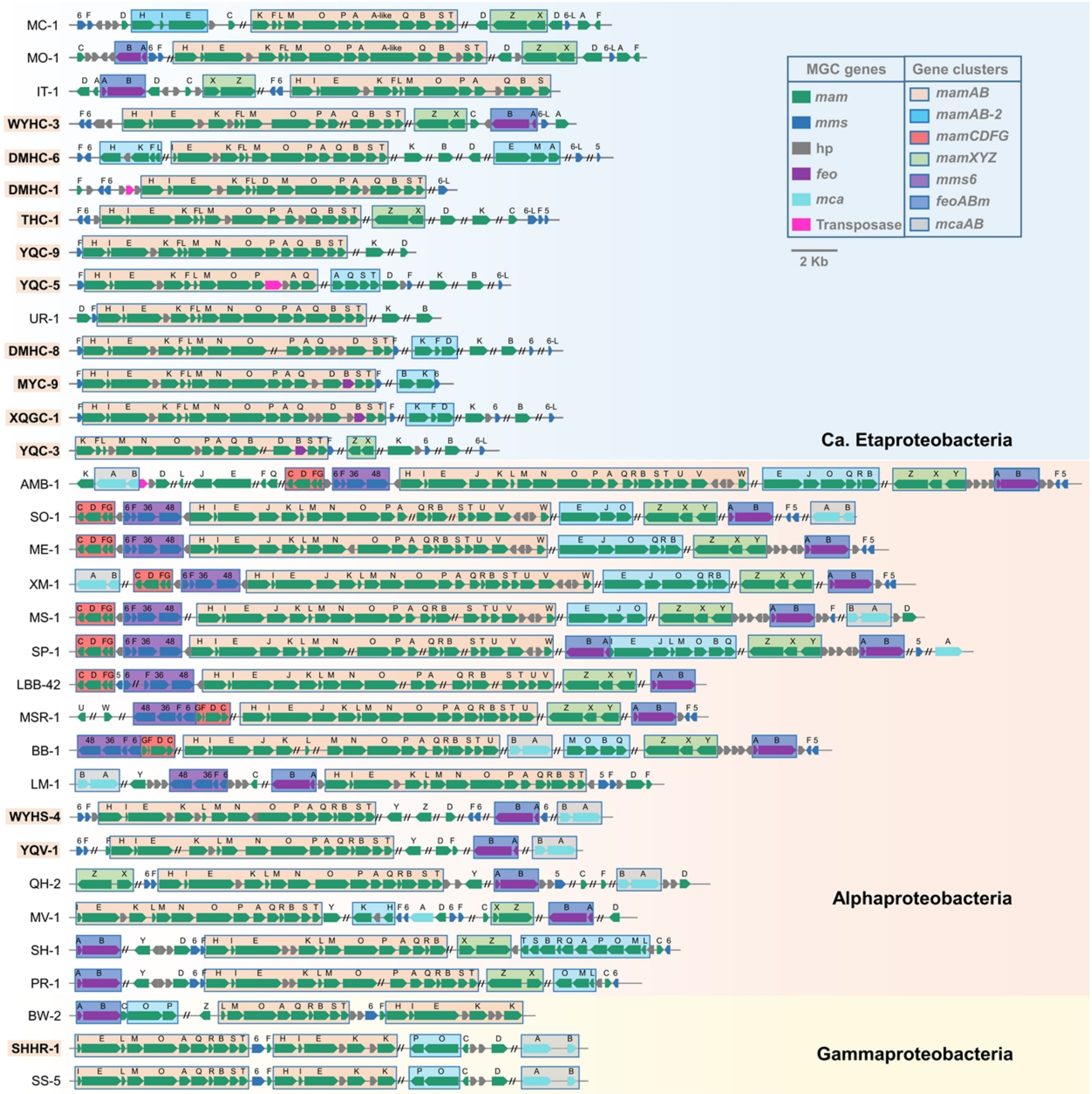

Figure S22. Gene organization and order of MGCs in MTB strains affiliated with the Pseudomonadota phylum. Strain names in bold and yellow background represent genomes reported in this study.

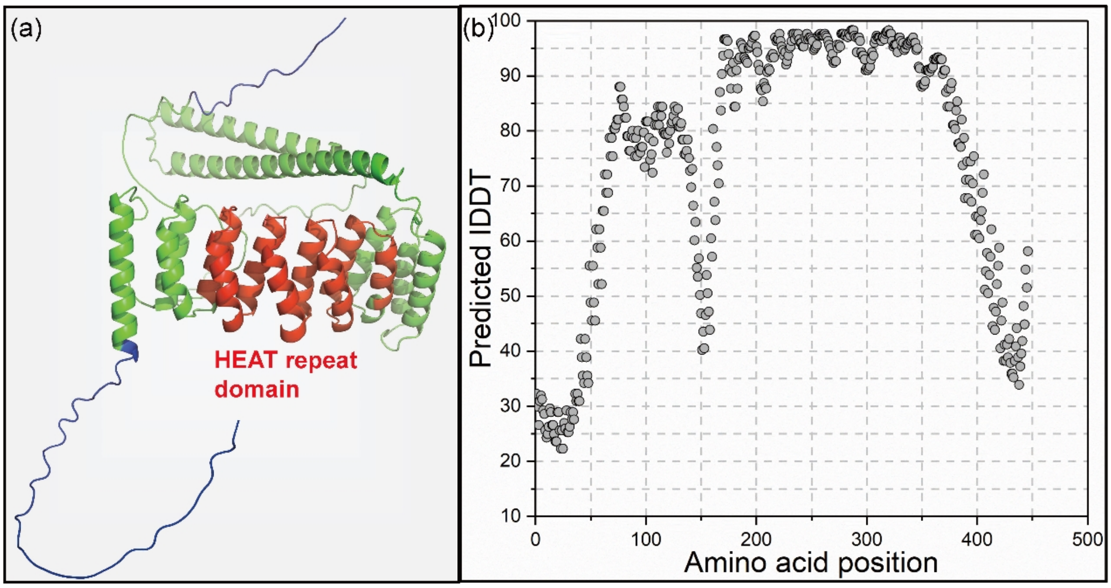

Figure S23. (a) Modeling of the 3-D structure of the Mad23 protein from strain YQR-1 with an average pLDDT 75.3. The HEAT repeat domain is marked in red. (b) Scatter plot of the confidence of amino acid in (a). The x-axis represents the amino acid positions. The y-axis represents the amino acid pLDDT value.

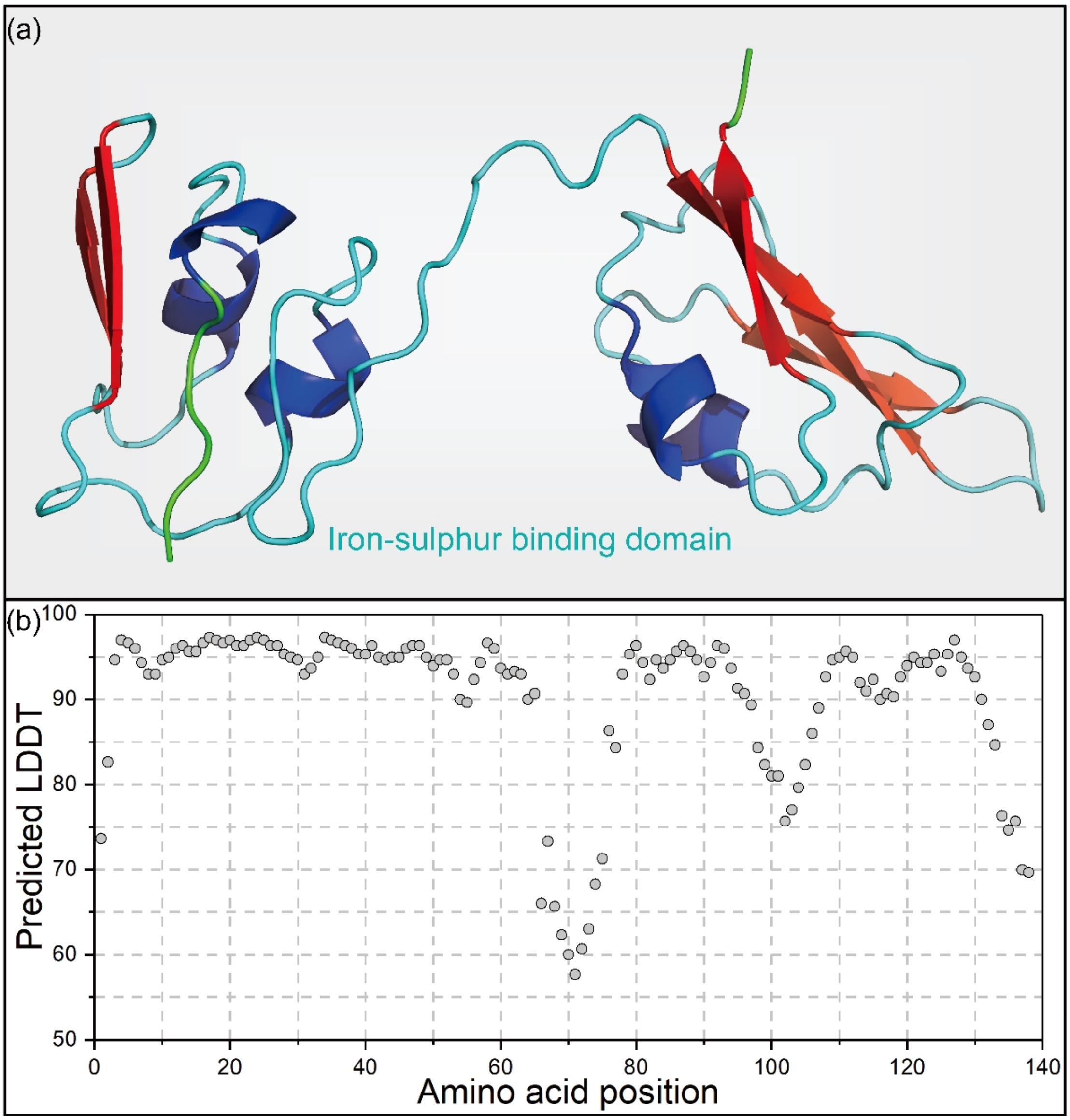

Figure S24. (a) Modeling of the 3-D structure of the Mad9 protein from strain BW-1 with an average pLDDT 91.6. The iron-sulphur binding domain is marked in cyan; the helixes and pleated sheets in the iron-sulphur binding domain are marked in green and red, respectively. (b) Scatter plot of the confidence of amino acid in (a). The x-axis represents the amino acid position. The y-axis represents the amino acid pLDDT value.

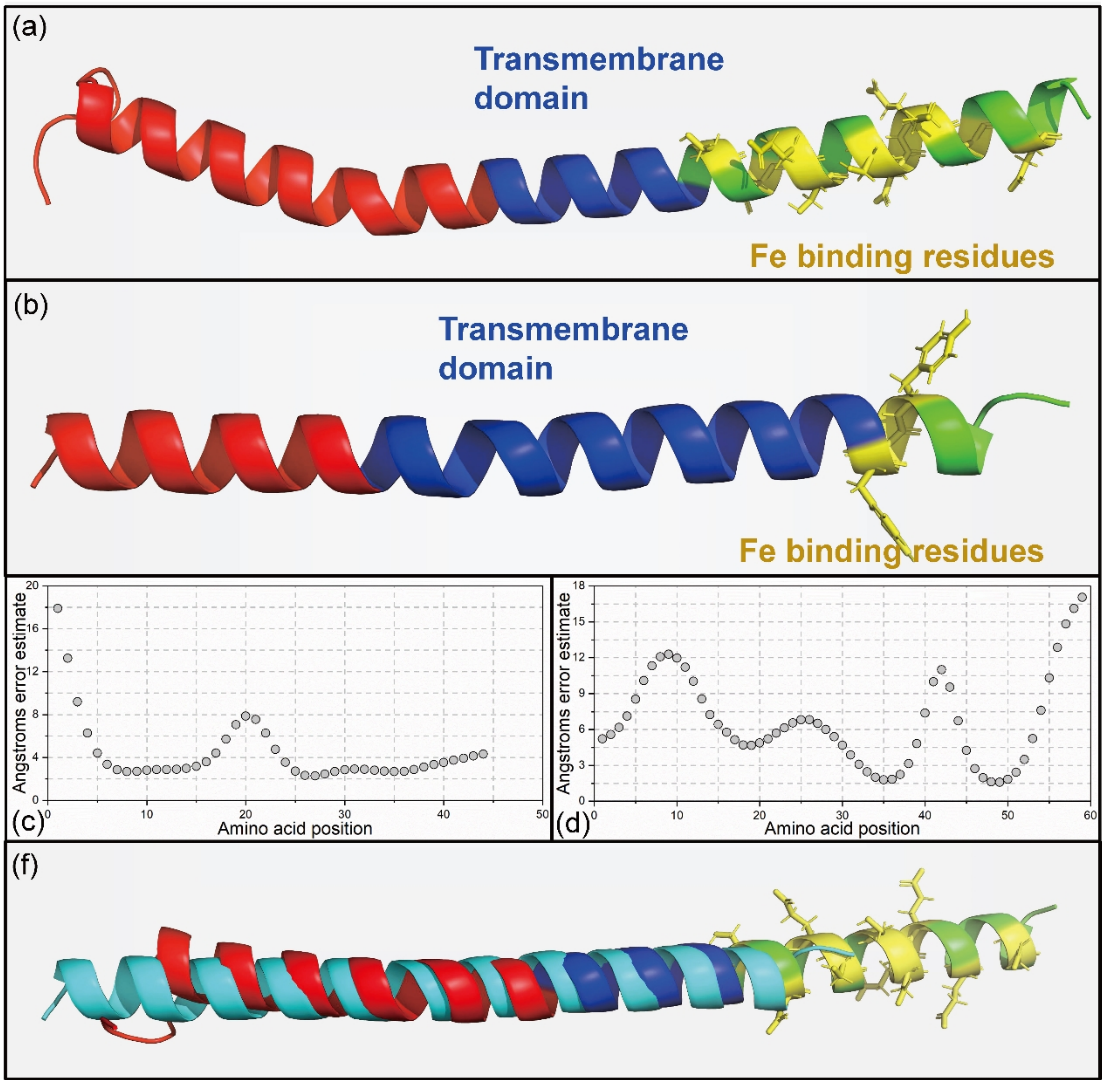

Figure S25. Modeling of the 3-D structures of the proteins Mms6 and Mms6-L. The transmembrane domains, regions outside the magnetosome membrane, and regions inside the magnetosome membrane are marked in blue, red, and green, respectively. Fe binding residues are highlighted in yellow. (a) and (b) Predicted 3-D structure of the Mms6 protein from strain AMB-1 and Mms6-L protein from strain THC-1 with the pLDDT 83 and 91, respectively. (c) and (d) Scatter plots of the confidence of amino acid in (a) and (b), respectively. The x-axis represents the amino acid position. The y-axis represents the error estimate (Å) of each amino acid. (f) 3-D structure comparison between (a) and (b) (in cyan) with the RMSD 3.7 Å.

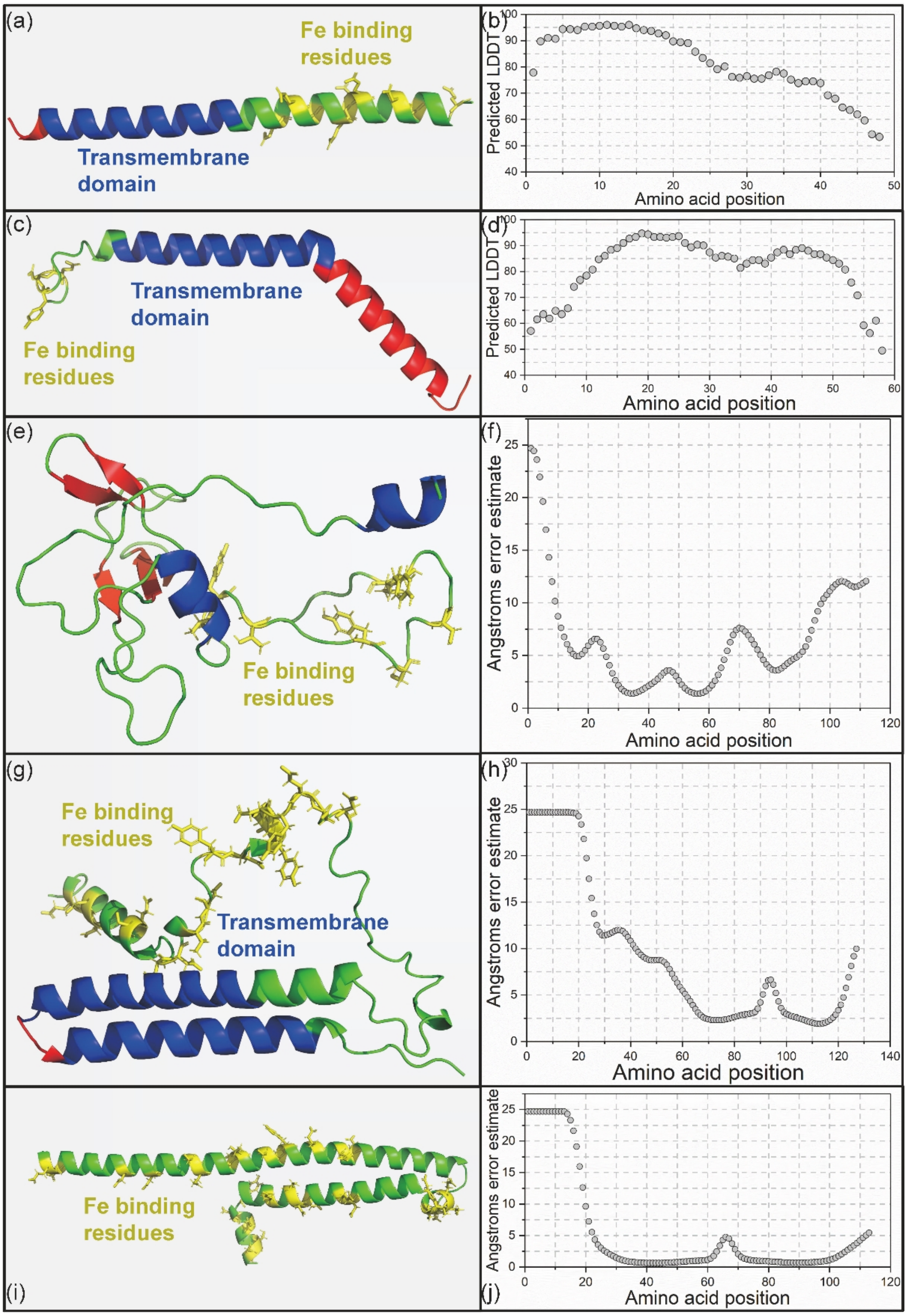

Figure S26. Modeling of the 3-D structures of the potential Fe binding Mad proteins. The transmembrane domains, regions outside the magnetosome membrane, and regions inside the magnetosome membrane are marked in blue, red, and green, respectively. Fe binding residues are highlighted in yellow. (a), (c), (e), (g), and (i) Predicted 3-D structures of the proteins Mad3-5, Mad8, and Mad19 from strain BW-1 with the pLDDT 53-83. (b), (d), (f), (h), and (j) Scatter plots of the confidence of amino acid in (a) (c), (e), (g), and (i), respectively. The x-axis represents the amino acid position. The y-axis represents the error estimate (Å) or amino acid pLDDT value.

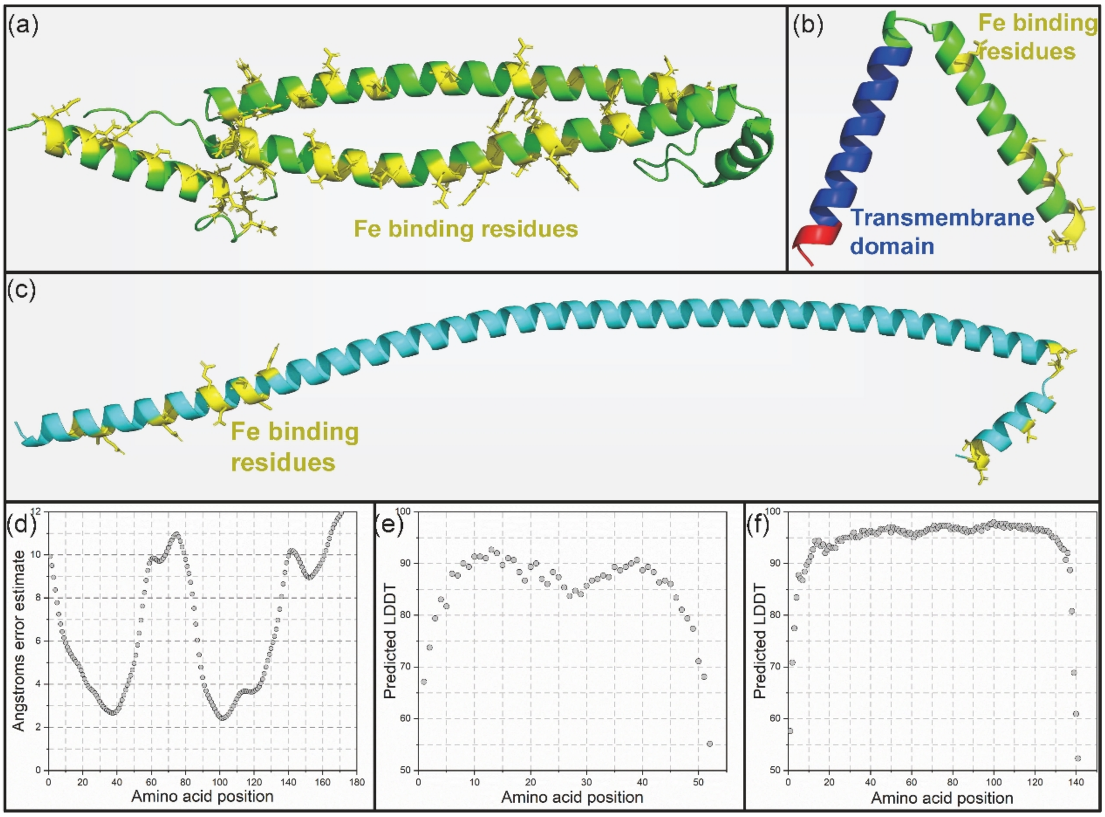

Figure S27. Modeling of the 3-D structures of the potential Fe binding Man proteins. The transmembrane domains, regions outside the magnetosome membrane, and regions inside the magnetosome membrane are marked in blue, red, and green, respectively. Fe binding residues are highlighted in yellow. (a) - (c) Predicted 3-D structure of the proteins Man1, Man3, and Man4 from strain YQR-1 with the average pLDDT 59-94.4. (d) - (f) Scatter plots of the confidence of amino acid in (a), (b), and (c), respectively. The x-axis represents the amino acid position. The y-axis represents the error estimate (Å) or amino acid pLDDT value.

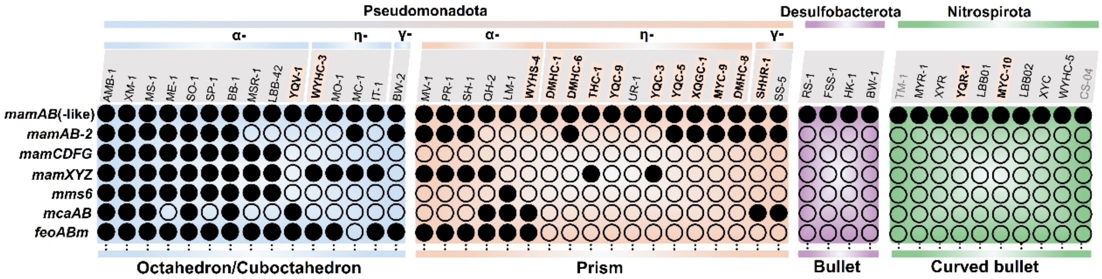

Figure S28. Relationship between magnetosome magnetite morphology and magnetosome gene cluster content. MTB strains are shown in groups according to their phylogeny and magnetosome crystal morphologies. The Greek letters α, η, and γ represent Alphaproteobacteria, Candidatus Etaproteobacteria, and Gammaproteobacteria classes. Strain names in bold and yellow background represent genomes reported in this study; gray font indicates high contamination (> 10%). Black solid circles indicate the presence of the corresponding magnetosome gene cluster, while black hollow circles correspond to its absence.

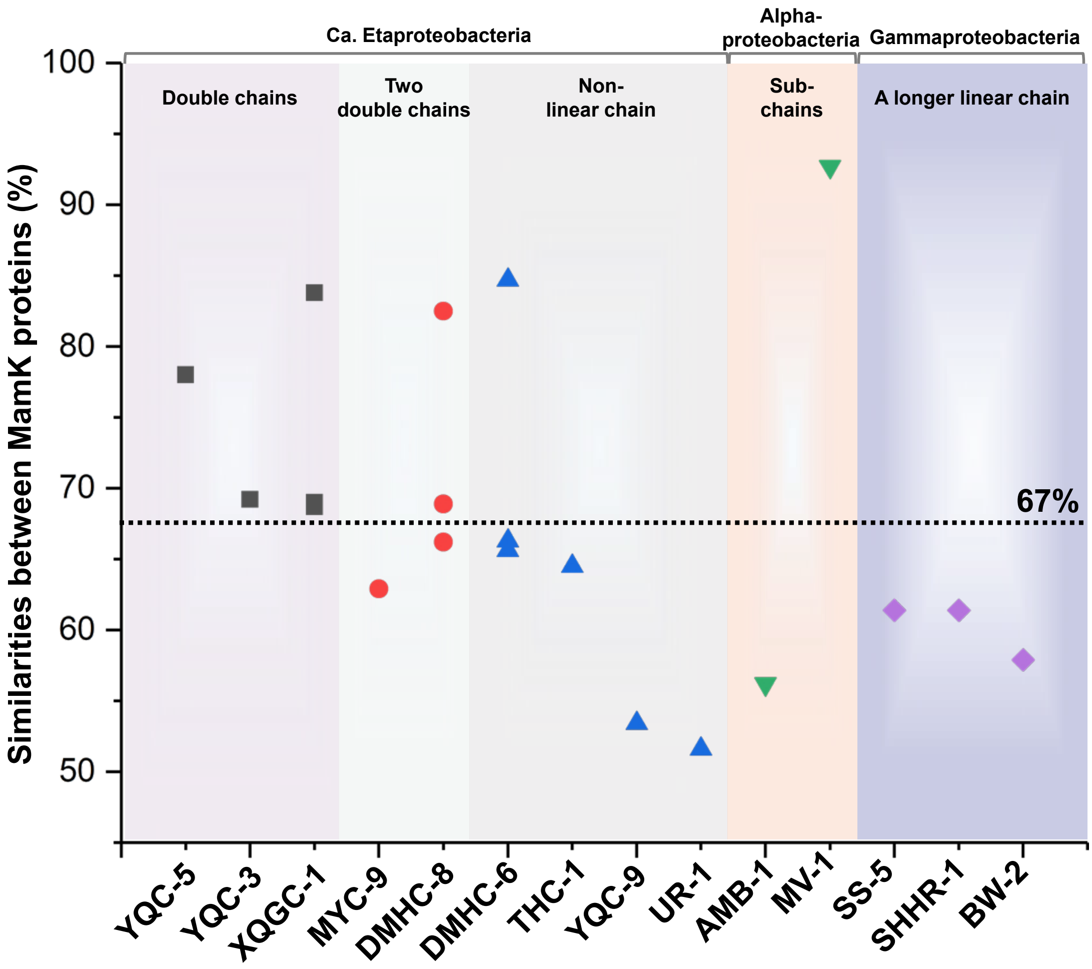

Figure S29. Relationship between similarities of MamK proteins and chain configuration of Proteobacteira strains. MTB strains are plotted on the horizontal axis and similarities between MamK proteins in each strain are represented on the vertical axis. Strain MYC-9 is an exception and contains two copies of the *mamK* gene and their coding protein sharing < ~67% similarity. A possible explanation is that this strain also contains another copy of the *mamK* gene, which is absent due to incomplete gene sequencing (~96.4% completeness).

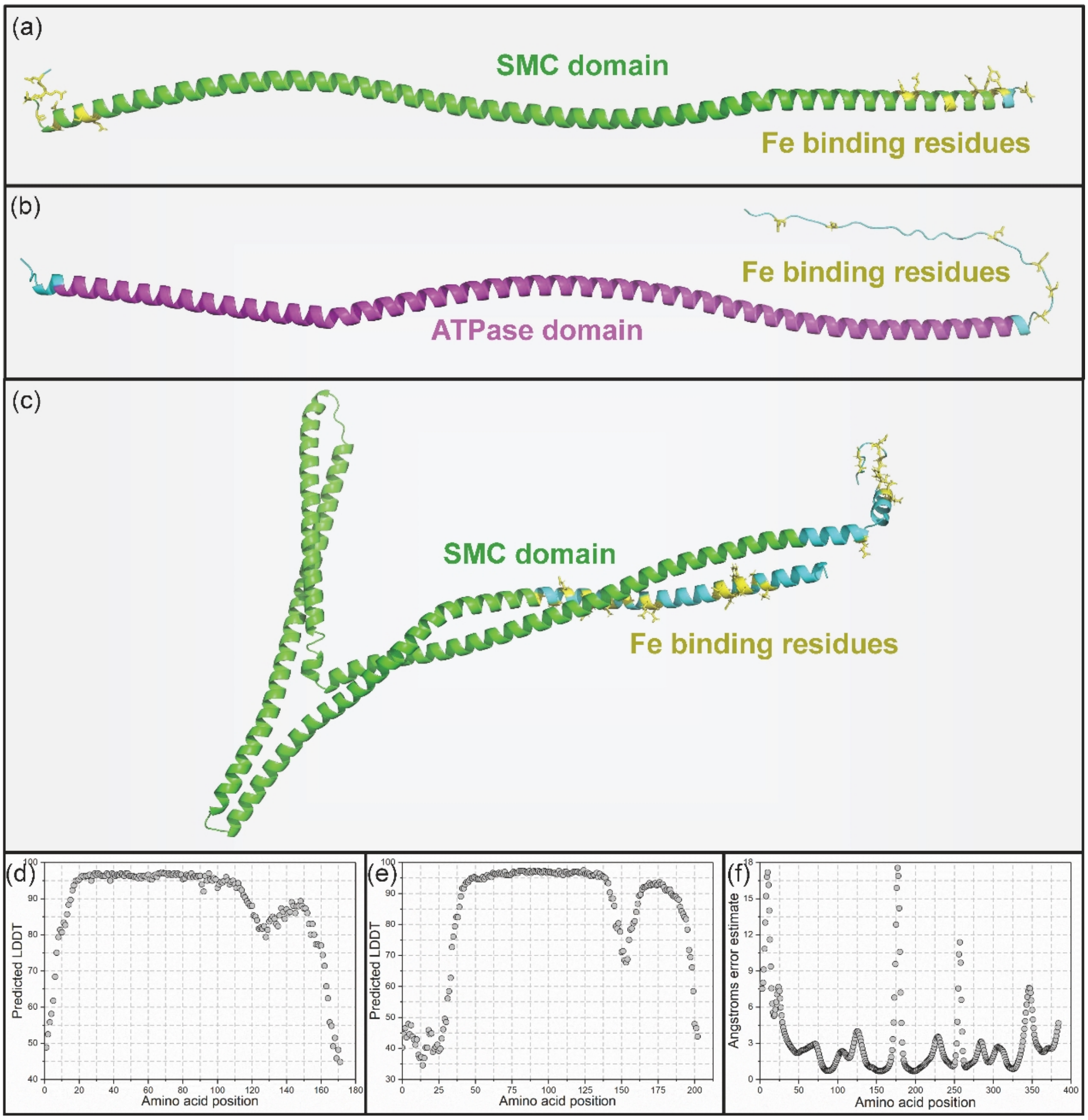

Figure S30. Modeling of 3-D structures of the SMC family proteins Mad24 and Man5. SMC domains and ATPase domain are marked in green and magenta, respectively. The potential Fe binding residues are highlighted in yellow. (a) - (c) Predicted 3-D structure of the Mad24 protein from strains BW-1 and RS-1, and Man5 protein from strain YQR-1 with the pLDDT 73-89.9. (d) - (f) Scatter plots of the confidence of amino acid in (a), (b), and (c), respectively. The x-axis represents the amino acid position. The y-axis represents the error estimate (Å) or amino acid pLDDT value.

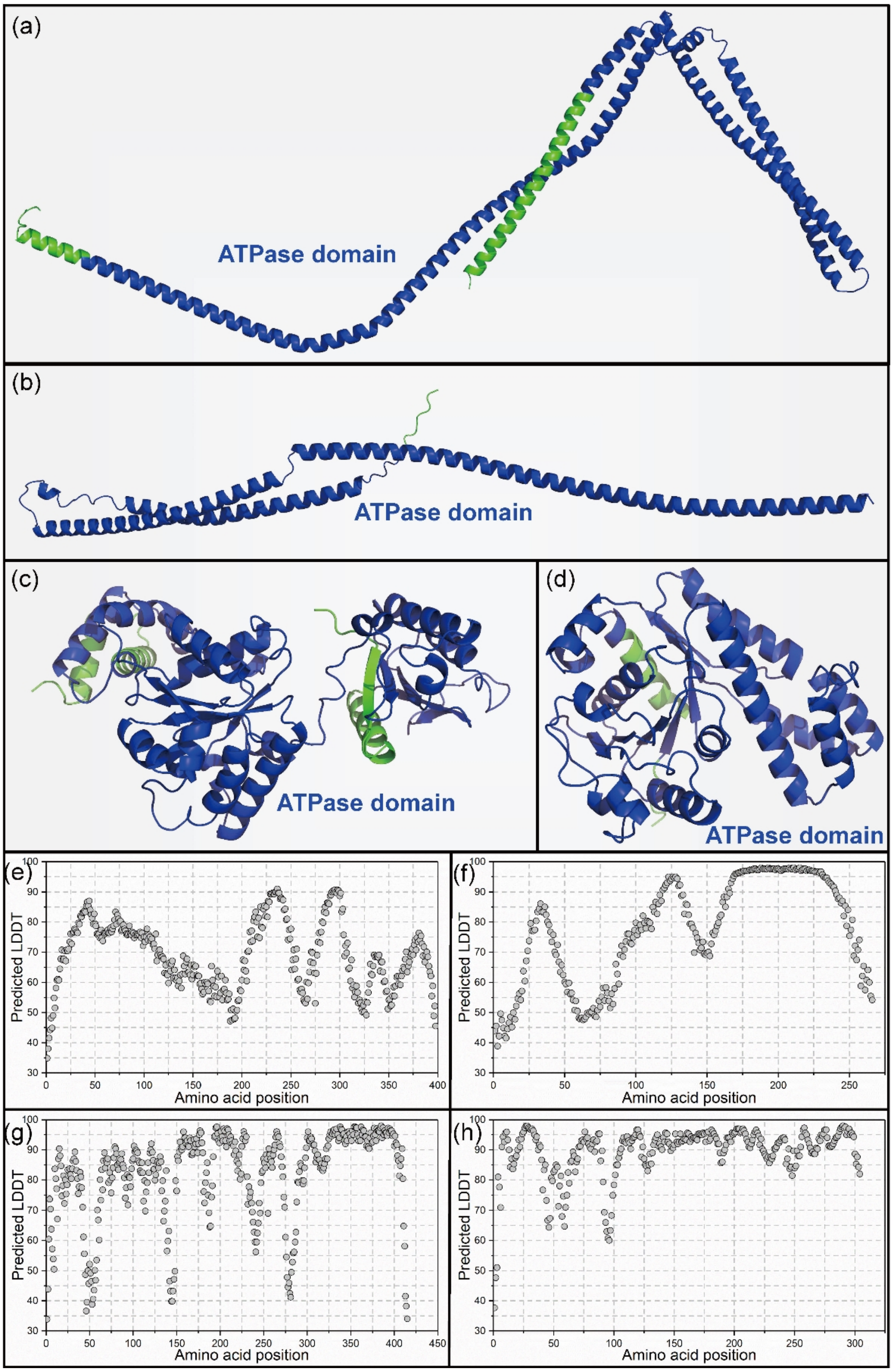

Figure S31. Modeling of the 3-D structures of the ATPase proteins in the Desulfobacterota phylum. ATPase domains are marked in blue. (a) – (d) Predicted 3-D structure of the proteins Mad22, Mad25, Mad27, and Mad29 from strain BW-1 with the pLDDT 72.3-90.4. (e) – (h) Scatter plots of the confidence of amino acid in (a), (b), (c), and (d), respectively. The x-axis represents the amino acid position. The y-axis represents the amino acid pLDDT value.

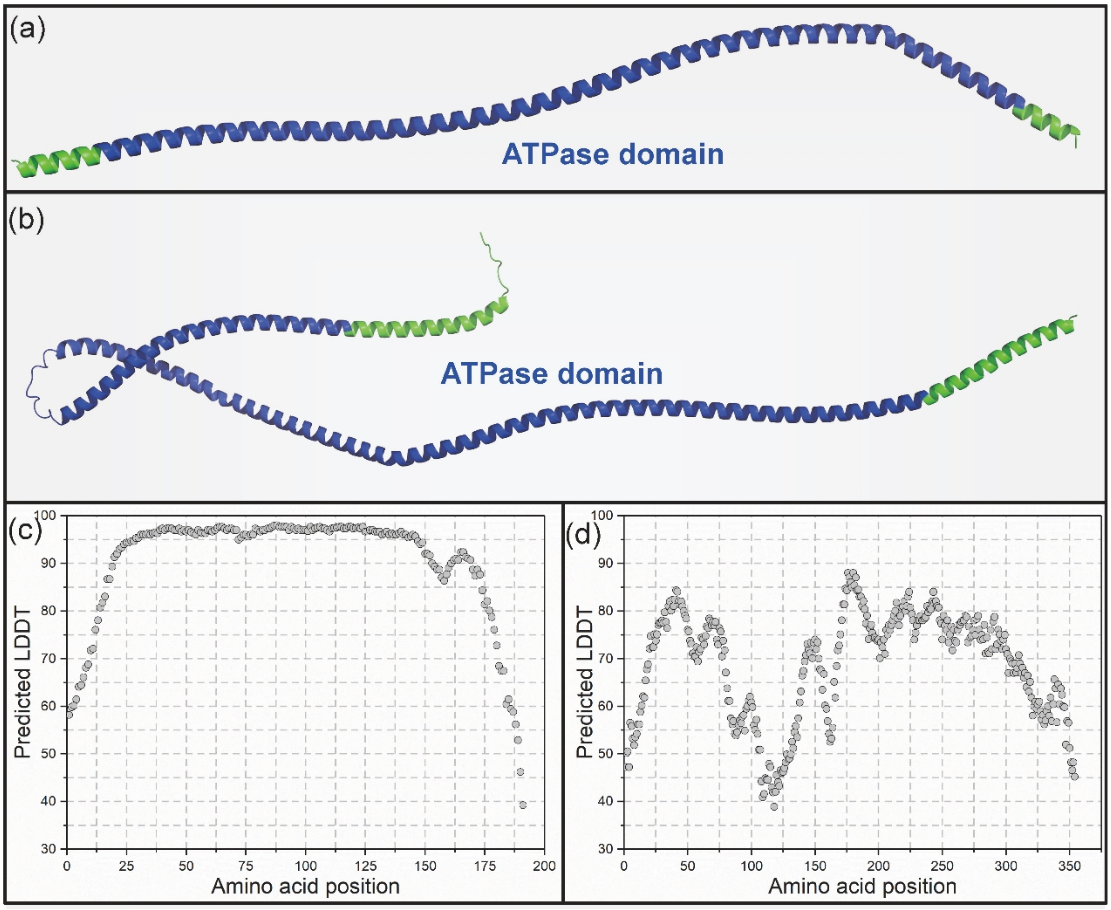

Figure S32. Modeling of the 3-D structures of the ATPase proteins in the Nitrospirota phylum. (a) and (b) Predicted 3-D structure of the proteins Mad26 and Man6 from strain YQR-1 with the pLDDT 90.7 and 68.9, respectively. (c) and (d) Scatter plots of the confidence of every amino acid in (a), and (b), respectively. The x-axis represents the amino acid position. The y-axis represents the amino acid pLDDT value.

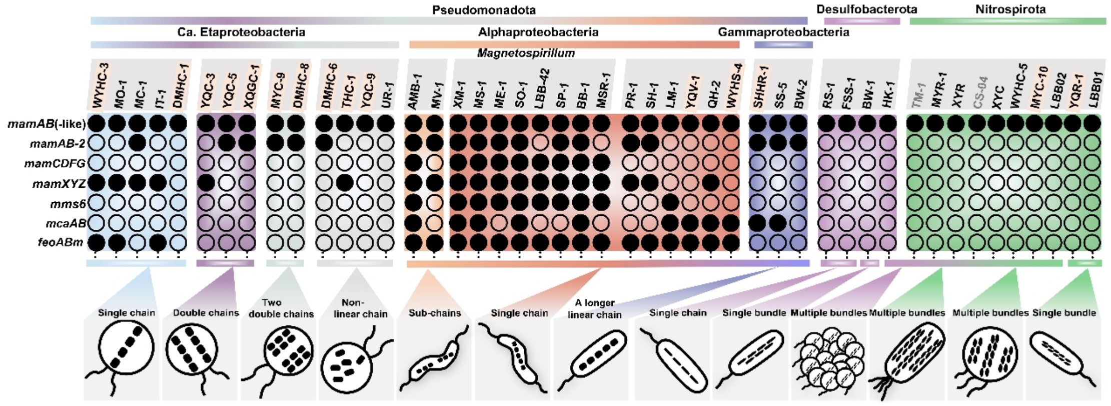

Figure S33. Relationship between magnetosome chain assemblies and magnetosome gene cluster contents. MTB strains are shown in groups according to their phylogeny and magnetosome chain assemblies. Strain names in bold and yellow background represent genomes reported in this study, and in gray font represent containing high contamination (> 10%). Black solid circles indicate the presence of the corresponding magnetosome gene cluster, while the black hollow circle indicates its absence.

Supplementary Tables

Table S1. Sampling locations and environmental factors at the time of sampling.

Sample site	Location	Sampling time	Water Temp. (°C)	pH	Salinity (‰)	Latitude (N)	Longitude (E)	Environment
Miyun Lake	Beijing city	June, 2016	19	7.52	0.19	40°31′11.7″	116°50′7.0″	Freshwater
Yuqiao Lake	Tianjing city	July, 2017	21	7.57	0.24	40°2′28″	117°27′21″	
Weiyanghu Lake	Xi'an city	October, 2018	21	8.38	0.58	34°24′12.1″	108°59′9.4″	
Daminghu Lake	Jinan city	January, 2017	3	7.87	0.38	36°40′19″	117°1′18.53″	
Xingqinggong Lake	Xi'an city	July, 2019	22	7.93	0.55	34°15′49.8″	108°58′9.3″	
Fuzhou Saltern	Dalian city	August, 2019	26	8.01	38.4	39°40′31.11″	121°59′33.98″	Salt pond

Notes: Miyun (MY) and Yuqiao (YQ) lakes are located in Beijing and Tianjin cities, respectively. Both lakes are artificial reservoirs that serve as the main drinking water source for city residents. Weiyanghu (WYH) and Xingqinggong (XQG) lakes are located in Xi'an city, Shaanxi province. They are artificial lakes in the Weiyang Lake Amusement Park and Xingqing Palace Park, respectively. Daminghu (DMH) lake is located in Daming Lake Park in the center of Jinan city, Shandong province. Fuzhou saltern (FZS) is located in Dalian city, Liaoning province, and contains many salt evaporation ponds in Bohai Bay.

Table S2. 16S rRNA gene sequences retrieved from laboratory microcosms.

Microcosms	Strain	No. of clones	Percentage of clones (%)	Most similar strain	Accession	Identity (%)	Reference
Microcosm-1	WYHC-3[&]	17	85	-	MN396581	100	[1]
	OTU1	3	15	*Azospirillum* sp. B510	AP010949	98.2	[21]
Microcosm-2	YQV-1[*]	16	80	XQGS-1	MZ268120	95.98	[22]
	OTU2	4	20	clone TIIF1	DQ297956	99.22	Unpublished
Microcosm-3	WYHS-4[*]	12	41.4	LBB-42	MH571849	91.35	[23]
	THC-1[&]	17	58.6	THC-1	MN396570	100	[1]
Microcosm-4	XQGC-1[#]	20	100	OTU51	GQ468517	99.73	[24]
Microcosm-5	MYC-9[#]	11	36.7	Clone 17	EU780677	99.79	[25]
	MYC-10[#]	13	43.3	MY3-11A	HM454282	99.93	[26]
	OTU3	6	20	clone SK14	KF182247	99.8	Unpublished
Microcosm-6	YQC-3	6	24	-	MN396541	100	[1]
	YQC-5[*]	5	20	CS308	X61607	99.25	[27]
	YQR-1[*]	14	56	MY3-5B	HM454281	91.89	[26]
Microcosm-7	YQC-9[*]	17	89.5	UR-1	MK813936	98.08	[28]
	THC-1	2	10.5	THC-1	MN396570	99.7	[1]
Microcosm-8	DMHC-1[&]	19	100	-	MN396579	100	[1]
Microcosm-9	DMHC-6[&]	18	90	-	MN396584	100	[1]
	OTU4	2	10	Clone KNA6S-1	LC122007	97.2	Unpublished
Microcosm-10	DMHC-8[&]	19	100	-	MN396585	100	[1]
Microcosm-11	SHHR-1[&]	9	34.6	-	KX344069	99.6	[4]
	FZSR-1	2	7.7	-	MW466803	100	[29]
	OTU5	4	15.4	*Bacillus* sp. M90	GQ340519	98.7%	[30]
	OTU6	11	42.3	*Sulfurimonas* sp. M-100	AB697382	97.6%	Unpublished

Notes: Sediments from Microcosms-1 and -3 were collected from Weiyanghu lake, Microcosm-4 is from Xingqinggong lake, Microcosm-5 is from Miyun lake, Microcosms-2, -6, and -7 are from Yuqiao lake, Microcosms-8 to -10 are from Daminghu lake, and Microcosm-11 is from a salt evaporation pond in Fuzhou saltern. A total of 15 uncultured MTB strains were studied here. Among them, five strains indicated by * are novel MTB species, and three strains indicated by # have been reported previously only based on 16S rRNA gene sequencing and have not yet been identified morphologically. These eight strains were, therefore, identified phylogenetically and morphologically at the single cell level via the coupled FISH-SEM method here. Seven other strains indicated by **&** were identified in our previous studies [1, 4]. MTB populations sampled from the same location may change over time due to subtle microcosm differences. MTB cells were separated magnetically from microcosms for subsequent molecular (i.e., 16S rRNA gene amplifying and sequencing) and microscopic (i.e., coupled FISH-SEM and TEM observations) analyses. In each case, 20 to 30 clones were used for 16S rRNA gene sequencing. Beside MTB sequences, some sequences (i.e., OTU1 to OTU6) retrieved from different magnetic collections may belong to non-MTB strains because they share a high similarity (identity > 97%) of 16S rRNA gene sequences with reported bacteria, such as *Azospirillum* sp. B510, *Azospirillum* sp. B510, and *Bacillus* sp. M90.

Table S3. Morphology and genome data for morphologically and phylogenetically identified MTB

Taxonomy	MTB strain	Accession: 16S rDNA	Accession: Genome	Shape	Cell: Diameter (μm)	Cell: Length (μm)	Chain configuration	Crystal model	Magnetosome: Number	Magnetosome: Length (nm)	Magnetosome: Width (nm)	Magnetosome: Axial Ratio	Genome: Size (Mb)	Genome: Scaffolds (no.)	Genome: GC content (%)	Genome: Completeness (%)	Genome: Contamination (%)	Reference
Ca. Eta-proteobacteria	*Magnetofaba australis* IT-1	JX534168	NZ_LVJN00000000	Cocci	1.4±0.3	--	Single chain	Octahedral	~10	83±26	74±23	0.89	4.99	21	61.3	98.7	0.84	[31]
	Magnetococcus marinus MC-1	NR_074371	NC_008576		1~2	--		Cubo-octahedral	14±3	83±14	72±11	0.93	4.72	1	54.17	100	0	[32, 33]
	Ca. Magnetococcus massalia MO-1	EF643520	LO017727		1.6±0.2	--			17±5	64±20	57±17	0.89	5.04	1	55.18	100	0	[34, 35]
	WYHC-3#	MN396581	JAMOBC000000000		1.4±0.2	--			12±3	60.0±13.4	56.9±13.4	0.95	4.1	354	59.92	87.1	7.84	[1]
	DMHC-1#	MN396579	JAMOAV000000000		1.3±0.2	--		Prismatic	10±2	96.4±26	74.5±22.5	0.77	4.68	186	54.53	95.6	0	[1]
	DMHC-6#	MN396584	JAMOAW000000000		2.1±0.2	--	Non-linear chain		70±13	69.3±8.7	45.7±5.6	0.66	3.7	118	45.99	95.3	2.10	[1]
	THC-1#	MN396570	JAMOBB000000000		2.5±0.2	--			35±15	70.6±8.6	42.3±5.2	0.6	4.4	87	52.19	99.2	1.68	[1]
	**YQC-9***	**ON340520**	**JAMOBI000000000**		**1.2±0.2**	--			**28±7**	**77.5±10.4**	**43.5±6.5**	**0.56**	**3.9**	**212**	**58.27**	**97.1**	**2.1**	**This study**
	Ca. Magnetaquicoccus inordinatus UR-1	MK813936	NZ_RXIU00000000		NA	--			NA	77.4±11.8	46.2±7.9	0.64	4.14	546	52.51	96.6	4.62	[28]
	YQC-3#	MN396541	JAMOBG000000000		1.3±0.1	--	Double chains		10±2	86.6±26.1	60.6±18.9	0.70	3.5	520	53.52	80.2	0	[1]
	**YQC-5***	**ON340535**	**JAMOBH000000000**		**1.2±0.2**	--			**12±3**	**81.2±14.9**	**61.7±12.6**	**0.75**	**5.7**	**553**	**50.73**	**91.6**	**3.62**	**This study**
	**XQGC-1***	**ON340524**	**JAMOBF000000000**		**1.1±0.1**	--			**11±3**	**77.9±15.1**	**62.4±12.7**	**0.80**	**4.8**	**722**	**56.48**	**89.4**	**8.17**	**This study**
	**MYC-9***	**ON340531**	**JAMOAY000000000**		**1.4±0.3**	--	Two double chains		**27±8**	**85.7±15.1**	**61.6±10**	**0.76**	**3.5**	**50**	**60.68**	**96.4**	**1.68**	**This study**
	DMHC-8#	MN396585	JAMOAX000000000		1.8±0.2	--			42±8	89.1±22.6	71.5±20.6	0.80	3.2	305	61.03	90	4.67	[1]
Alpha-proteobacteria	*Magnetospirillum magneticum* AMB-1	AP007255	NC_007626	Spirillum	0.4~0.6	3	Sub-chains	Cubo-octahedral	>15	~50	~45	0.85	4.97	1	65.09	100	0	[36, 36]
	Magnetospirillum sp. XM-1	KP966105	LN997848		NA	1~5	Single chain		~10	43.7	NA	0.85	4.83	1	65.64	100	0	[38, 39]
	Magnetospirillum magnetotacticum MS-1	M58171	NZ_JXSL00000000		0.2~0.4	4~6			NA	40~50	40~50	0.9	4.52	36	63.56	98.3	0	[40, 41]
	Magnetospirillum sp. ME-1	NA	NZ_CP015848		NA	NA			17±4	32.5±4.5	28.9±4.5	0.89	4.55	1	65.63	100	0	[42, 43]
	Magnetospirillum caucaseum SO-1	NR_149241	NZ_AONQ00000000		0.3	1.3~3.0			NA	NA	NA	NA	4.87	236	65.98	98.3	0	[44]
	Magnetospirillum kuznetsovii LBB-42	MH571849	NZ_PGTO01000000		0.5±0.1	2.7±0.9			15±6	38±7	NA	NA	4.40	69	63.44	99.16	0	[23]
	Magnetospirillum gryphiswaldense MSR-1	NR_121771	NC_023065		0.7~1.0	3~20			0~40	~42	~42	NA	4.37	1	63.2	100	0	[45, 46]
	Magnetospirillum marisnigri SP-1	NR_149242	NZ_LWQT00000000		0.3~0.4	2.5~0.4			NA	NA	NA	NA	4.62	131	64.73	99.9	1.26	[44]
	Magnetospirillum moscoviense BB-1	NR_149243	NZ_LWQU00000000		0.3	2.0~4.0			NA	NA	NA	NA	4.16	207	65.18	99.9	0	[44]
	**WYHS-4***	**ON340536**	**JAMOBD000000000**		**0.6±0.1**	**2.2±0.3**		Prismatic	**21±4**	**75.5±10**	**43±8**	**0.57**	**3.7**	**172**	**65.96**	**98.8**	**1.74**	**This study**
	Magnetovibrio blakemorei MV-1	NR_118660	NZ_MCGG00000000	Vibrio	0.2~0.4	1~3	Sub-chains		10±4	53	35	0.63	3.64	91	54.29	98.32	0	[47, 48]
	Ca. Terasakiella magnetica PR-1	NA	FLYE01000000	Spirillum	0.5±0.2	1.6±0.3	Single chain		14±4	44±13	34±11	0.77	4.41	48	45.97	99.9	1.26	[49]
	Terasakiella sp. SH-1	NA	NZ_CP038255		NA	NA			NA	48.3±8.9	35.7±5.2	0.74	3.83	1	47.53	100	0	[50]
	Rhodospirillaceae sp. LM-1	JF490044	CACUVI000000000		NA	NA			NA	NA	NA	NA	3.57	30	59.52			[51]
	Magnetospira sp. QH-2	EU675666	NZ_FO538765	Vibrio	0.8±0.2	2.0±0.4			16±5	81±23	58±20	0.71	4.05	1	59.47	100	0	[52]
	**YQV-1***	**ON340537**	**JAMOBE000000000**		**0.8±0.1**	**2.5±0.2**		**Cubo-octahedral**	**19±2**	**88.6±13**	**82±11**	**0.93**	**4.00**	**228**	**66.22**	**97.5**	**0.50**	**This study**
Gamma-proteobacteria	BW-2	HQ595728	CP032507	Rod	2.2±0.2	4.4±0.6	A longer linear chain	Cubo-octahedral	30±9	67±16	63±15	0.97	4.07	1	52.6	100	0	[53, 54]
	SHHR-1#	KX344069	JAMOBA000000000		0.9±0.1	2.5±0.5		Prismatic	~15	72.9±15.7	52.6±11	0.73	3.06	535	62.67	89.1	5.46	[4]
	SS-5	HQ595729	CP032508		1.2±0.1	2.5±0.5			20±7	86±27	63±19	0.74	3.73	1	61.65	100	0	[53, 55]
Desulfo-bacterota	*Desulfovibrio magneticus* RS-1	NR_074958	NC_012796	Vibrio	0.9~1.5	3~5	Single chain	Bullet	10~15	~60	~40	0.5	5.32	1	62.77	100	0	[56]
	Desulfovibrio sp. FSS-1	LC311577	NZ_BLTE00000000		0.8 ± 0.1	2.9 ± 0.9			9±6	53.9±11	25.5±3.3	NA	4.46	58	67.54	99.4	0.60	[57]
	Ca. Magnetomorum sp. HK-1	EU717681	JPDT01000000	MMP	5.7±1.1	--	Multiple bundles		314-1108	80.1±16.1	33.6±3.5	0.48	14.29	3036	34.61	96.2	5.88	[58, 59]
	Desulfamplus magnetovallimortis BW-1	JN252194	NZ_FWEV00000000	Rod	NA	NA	Single bundle		NA	NA	NA	NA	6.68	346	40.72	92.8	0.84	[60]
Nitrospirota	*Ca.* Magnetobacterium bavaricum TM-1	X71838	LACI00000000	Rod	NA	NA	Multiple bundles	Curved-bullet	~1000	NA	NA	NA	6.31	2751	47.31	87.1	42.8	[61]
	Ca. Magnetobacterium casensis MYR-1	MT703955	NZ_JMFO00000000		1-3	6-8			~1000	10-180	35-40	NA	3.42	70	48.87	80.5	6.14	[62, 63]
	Ca. Magnetobacterium cryptolimnobacter strain XYR	NA	JAGYWH000000000		1-2	5-7			150	30-130	NA	NA	4.23	195	48.61	96.97	2.73	[64]
	**YQR-1***	ON340538	JAMOBJ000000000		**0.8±0.1**	**2.7±0.2**	Single bundle		**51±11**	**88±22.8**	**38±3.8**	**0.47**	**3.5**	**110**	**42.33**	**97.7**	**0.91**	**This study**
	Ca. Magnetomonas plexicatena LBB01	MK632185	CP049016	Spirillum	0.5±0.1	2.0±0.4			33±9	108±21.1	45 ± 8.1	0.45	3.27	1	41.96	100	0	**[65]**
	**MYC-10***	ON342894	JAMOAZ000000000	Cocci	**1.5±0.2**	--	Multiple bundles		**43±7**	**89.2±14.8**	**29.3±3.3**	**0.47**	**3.5**	**98**	**45.01**	**96.4**	**0.91**	**This study**
	Ca. Magnetominusculus linsii LBB02	MK632186	JAKOEO000000000		1.2-1.5	--			NA	NA	NA	NA	3.47	142	47.48	91.52	0	**[65]**
	Ca. Magnetoovum chiemensis CS-04	JX402654	JZJI00000000		2.5	--			NA	NA	NA	NA	3.82	1019	40.42	81.6	24.1	[61]
	Ca. Magnetoovum sp. WYHC-5	OL423397	JAKKUN000000000		3.58±0.4	--			NA	70.4±27	27.5±6.3	39.1	3.18	307	37.99	95.3	0.91	[66]
	Ca. Magnetomicrobium Cryptolimnococcus XYC	NA	JAGYWI000000000		2-4	--			50-145	45-135	NA	NA	3.59	91	37.75	99.94	1.82	[64]
Ca. Omnitrophus	*Ca.* Omnitrophus magneticus SKK-01	JN412733	JYNY00000000	Cocci	2.5±0.1	--	Multiple bundles	Bullet	175±15	110±23	37±5	0.34	3.15	656	35.75	70.3	NA	[61, 67]

Note: Eight names in bold with * are novel strains identified morphologically here. Seven strains marked by # are novel genomes acquired here. The sizes of fifteen genomes range from 3.2 Mb to 5.7 Mb, and GC contents range from 42.33% to 66.22% (Figs. S1-S15). Nine of the genomes were linked with the above MTB according to the matched 16S rDNA sequence (WYHC-3, SHHR-1, THC-1, DMHC-8, MYC-10, YQC-3, YQR-1, YQC-5, and XQGC-1) (Table S4). The other six genomes also correspond to MTB strains because they were retrieved from phylogenetically and morphologically identified MTB. To compare genomic level similarity, average nucleotide identity (ANI) analysis was conducted for newly identified MTB and published MTB. Except for strain SHHR-1, which has high similarity (99.04%) with strain SS-5, the remaining MTB studied here have low similarity (59.03%-88.2%) with published MTB, which suggests that they represent novel phylogenetic branches according to the species criterion level of 95% [68] (Table S5). Phylogenetic analysis of these novel MTB strains based on whole genomes (Fig. 2a) indicate that the fifteen strains are affiliated with the Alphaproteobacteria (strains WYHS-4 and YQV-1), Gammaproteobacteria (strain SHHR-1), and 'Candidatus Etaproteobacteria' (strains WYHC-3, MYC-9, YQC-3, YQC-5, YQC-9, DMHC-1, DMHC-6, DMHC-8, THC-1 and XQGC-1) classes of the Proteobacteria phylum and the Nitrospirae phylum (strains MYC-10 and YQR-1).

Table S4. 16S rDNA sequences in MTB genomes.

Strain	Accession		16S rDNA sequence length in genome (bp)	Identities between genome and 16S rRNA gene (%)
	Genome	16S rRNA		
WYHC-3	JAMOBC000000000	MN396581	1455	100
THC-1	JAMOBB000000000	MN396570	357	100
YQC-3	JAMOBG000000000	MN396541	1462	99.9
YQC-5	JAMOBH000000000	ON340535	346	100
XQGC-1	JAMOBF000000000	ON340524	662	99.8
SHHR-1	JAMOBA000000000	KX344069	304	99.6
MYC-10	JAMOAZ000000000	ON342894	1488	99.9
YQR-1	JAMOBJ000000000	ON340538	1044	100

Table S5. ANI values between MTB genomes.

ANI (%)	DMHC-1	DMHC-6	DMHC-8	MYC-9	THC-1	WYHC-3	XQGC-1	YQC-3	YQC-5	YQC-9	WYHS-4	YQV-1	SHHR-1	MYC-10	YQR-1
MC-1	65.45	64.46	65.45	66.04	64.97	66.31	65.71	65.95	64.49	65.45	62.71	62.74	63.46	63.36	60.33
MO-1	65.91	64.15	65.82	65.98	64.72	66.15	65.99	66.15	64.29	65.82	63.60	63.59	64.46	63.36	59.86
IT-1	66.58	63.57	66.55	66.19	64.51	67.71	66.33	66.46	64.99	66.79	65.22	65.20	66.17	63.61	60.54
UR-1	66.41	65.73	70.74	70.51	65.73	65.32	70.91	70.28	67.33	66.93	62.85	62.60	63.36	62.86	60.39
DMHC-1	--	65.07	67.47	67.24	66.01	66.90	67.36	66.78	66.51	66.44	64.32	64.27	63.48	62.78	60.16
DMHC-6	65.33	--	65.66	69.92	65.26	64.26	66.03	65.94	66.15	65.02	61.76	62.18	61.71	62.28	59.73
DMHC-8	67.70	65.8	---	77.01	66.24	66.97	76.83	74.56	68.11	68.23	64.34	63.80	64.23	62.90	59.72
MYC-9	66.63	65.83	76.66	---	65.78	66.04	88.20	74.24	68.82	67.36	63.49	63.17	63.87	62.63	59.92
THC-1	66.20	65.19	66.15	66.62	---	65.56	66.35	66.27	65.75	65.30	62.66	62.89	62.55	62.37	59.89
WYHC-3	67.24	62.24	67.31	66.64	65.65	---	66.61	66.66	65.8	66.71	64.94	65.01	65.23	62.97	60.36
XQGC-1	67.46	66.10	75.95	85.41	66.57	66.64	---	74.23	69.78	67.54	63.44	63.83	64.04	62.82	59.42
YQC-3	67.09	66.36	74.96	74.32	66.30	66.47	75.12	---	68.09	67.61	63.62	63.58	64.05	63.82	63.51
YQC-5	66.73	66.13	68.03	70.86	66.03	65.81	69.79	68.08	---	69.40	63.20	62.79	63.02	62.70	60.20
YQC-9	66.49	64.76	68.14	67.82	65.45	66.36	67.69	67.24	69.45	---	64.57	64.87	64.43	62.91	59.42
AMB-1	64.47	61.71	64.05	64.89	62.86	65.35	63.62	63.38	62.9	64.89	70.04	70.25	65.55	63.15	60.45
MSR-1	64.19	61.82	63.48	64.78	62.74	65.07	63.39	63.43	62.71	64.12	68.98	69.30	65.01	63.07	60.64
SP-1	64.05	61.09	63.63	63.55	62.76	65.38	63.44	63.69	62.84	64.18	69.70	69.70	65.49	61.05	60.19
PR-1	64.03	61.53	63.79	63.82	62.53	65.22	64.27	63.17	63.16	64.36	68.84	69.37	64.97	60.77	60.06
MV-1	63.81	62.02	62.86	64.2	63.07	64.42	63.00	63.27	62.63	63.34	66.77	65.74	64.55	62.37	59.74
QH-2	64.6	61.95	64.01	64.67	63.43	65.75	63.59	63.92	63.04	64.20	68.41	67.55	65.61	63.05	60.52
SH-1	62.72	61.68	61.75	64.23	61.69	63.21	62.09	63.16	61.77	62.26	63.92	63.32	62.18	63.74	59.97
WYHS-4	64.28	61.68	64.62	64.8	62.82	64.77	64.23	63.69	63.21	65.12	---	69.96	65.94	62.45	59.73
YQV-1	64.81	62.14	63.66	64.42	62.80	65.33	63.90	63.52	63.02	65.22	69.93	---	65.66	62.73	59.50
SS-5	64.35	61.89	64.46	65.25	62.90	65.41	64.26	64.41	63.36	64.66	65.73	65.94	99.04	63.73	60.72
BW-2	62.55	62.06	62.29	64.38	62.37	63.71	62.78	63.41	61.41	62.65	62.60	62.60	65.59	63.47	60.04
SHHR-1	64.14	61.72	64.81	65.14	62.82	65.13	64.59	64.35	63.18	64.76	65.53	65.78	---	63.50	59.86
BW-1	61.4	60.83	59.89	63.59	60.27	61.74	61.12	62.04	61.37	60.14	59.83	59.94	60.28	62.89	60.70
HK-1	60.64	61.24	59.54	62.99	60.98	59.80	60.42	61.32	61.44	59.18	59.03	59.09	59.15	62.40	60.40
RS-1	63.26	60.59	62.33	64.44	61.67	64.24	62.48	63.32	62.02	62.99	63.61	63.80	63.78	63.80	60.25
MYR-1	61.53	59.76	61.08	63.41	60.38	62.18	61.08	75.19	60.79	61.04	61.49	61.63	61.06	66.78	65.50
CS-04	60.96	59.73	60.31	63.17	59.94	61.03	60.63	62.02	60.41	60.41	60.77	61.14	62.26	65.01	64.30
YQR-1	59.85	59.69	59.59	62.69	59.85	60.36	59.62	63.58	59.96	59.58	59.59	59.67	59.49	66.91	---
XYC	59.96	59.87	59.43	59.37	60.02	61.14	59.72	61.05	59.55	58.89	59.76	59.68	59.90	64.34	64.11
WYHC-5	60.49	60.14	59.98	59.96	60.08	60.67	59.54	61.71	59.45	59.10	59.00	59.41	60.02	64.32	64.28
MYC-10	59.89	59.68	59.27	63.03	59.34	60.74	59.82	61.90	60.06	60.07	60.21	60.41	60.05	---	66.38

Table S6. Function and conservation score of magnetosome genes.

Gene	Function	Conservation score (%)			Reference
		Pseudomonadota	Desulfobacterota	Nitrospirota	
mamA/mms24	Sort proteins and activate magnetosome vesicles	100	100	100	[69]
mamB	Magnetosome vesicle formation and ferrous iron transport	100	100	100	[70, 71]
mamC/mms13	Regulate crystal size	66.7	0	0	[72, 73]
mamD/mms7	Control crystal size, morphology, and magnetosome vesicle growth	93.9	0	0	[73, 74]
mamE	Serine protease, magnetosome protein localization, redox control, and protein location.	100	100	100	[74, 75]
mamF/mmsF	Control crystal size and magnetite homogeneity	100	0	0	[76]
mamG	Control crystal size	27.3	0	0	[72]
mamH	Iron transport	100	0	0	[77, 78]
mamI	Magnetosome vesicle formation and magnetite nucleation	100	100	100	[71, 79]
mamJ	Interaction with *mamK* and magnetosome chain assembly	27.3	0	0	[80]
mamK	Organize magnetosome into chain	100	100	100	[81]
mamL	Magnetosome membrane formation	100	100	0	[71, 79]
mamM	Ferrous iron transport, crystallization initiation and protein localization	100	100	100	[70]
mamN	Transport H^+ and maintain vesicle pH	60.6	50%	0	[79, 82]
mamO	Precipitation of iron oxide particles	100	0	100	[75, 83]
mamP	Control redox, crystal size, and crystal number	100	100	100	[84]
mamQ	Magnetosome membrane formation	100	100	100	[71, 79]
mamR	Control crystal size and number	57.6	0	0	[71, 79]
mamS	Control crystal size	100	0	0	[71]
mamT	Control crystal growth and redox	100	0	0	[71]
mamU	No role in magnetosome biomineralization	27.3	0	0	[71]
mamV	No role in magnetosome biomineralization	21.2	0	0	[71]
mamW	No role in magnetosome biomineralization	21.2	0	0	[71]
mamX	Balance the redox state of iron	57.6	0	0	[78, 85]
mamY	Anchor magnetosomes alone the positive curvature line	48.5	0	0	[86]
mamZ	Iron transport and redox control	63.6	0	0	[77]
mms5	Regulate magnetite biomineralization process	33.3	0	0	[87]
mms6	Regulate crystal size and morphology	90.9	0	0	[73, 87]
mms6-L	Homologous gene of mms6 gene; may regulate crystal morphology	42.4	0	0	This study
mms36	Regulate crystal size	30.3	0	0	[79]
mms48	Regulate crystal size	30.3	0	0	[79]
mcaA	Recognizes the positive curvature of the inner cell membrane and create space for addition of new magnetosomes between pre-existing magnetosomes	39.4	0	0	[88]
mcaB	Localise to magnetosomes	33.3	0	0	[88]
mad1-11	May play roles in magnetite biomineralization	0	100	100	[89]
mad17-30	Mostly unknown; mad17 and mad30 may play a role in iron transport; mad28 may be involved in the positioning and segregation of magnetosome chain(s)	0	100	100	[89]
man1-6	May be involved in magnetosome synthesis or chain arrangement and segregation processes	0	0	100	[63]

Note: Conservation scores in this study represent the relative measure of evolutionary conservation of MGC genes in MTB groups, calculated by: (number of strains containing a MGC gene)/(number of analyzed MTB strains).

Table S7. Potential functions of several *mad* and *man* genes.

Gene	Potential functions	Methods	References
mad1	Contains three CXXCH heme-binding motifs. Related to magnetic response of MTB cell and magnetosome morphology control.	Gene mutation	[90]
mad2	Related to magnetic response of MTB cell and magnetosome morphology control.	Gene mutation	[90]
mad3	Contains a hydrophobic N-terminal with a transmembrane domain and a hydrophilic C-terminal. May be a magnetite-binding protein.	Bioinformatics analysis	This study
mad4	May be a magnetite-binding protein.	Bioinformatics analysis	This study
mad5	May be a magnetite-binding protein.	Bioinformatics analysis	This study
mad6	Contains a NapH nitrate reductase domain. Related to magnetic response of MTB cell.	Gene mutation	[90]
mad8	Contains a hydrophilic N-terminal and two transmembrane domains. May be a magnetite-binding protein.	Bioinformatics analysis	This study
mad9	Ferredoxin-like protein. May regulate redox in magnetosome vesicles.	Bioinformatics analysis	This study
mad10	Magnetite-binding protein may be involved in magnetosome formation.	*In vitro* experiment	[91]
mad11	Magnetite-binding protein may be involved in magnetosome formation.	*In vitro* experiment	[91]
mad17	Homologous gene of *feoB* may play a role in ferrous ion transport.	Bioinformatics analysis	[89]
mad19	May be a magnetite-binding protein.	Bioinformatics analysis	This study
mad22	Contains an ATPase domain affiliated with the SMC super family. May be a subunit of the ATPase which provides energy for assembling magnetosomes into chain structures.	Bioinformatics analysis	This study
mad23	Contains a HEAT repeat domain. May be involved in protein recruitment.	Bioinformatics analysis	This study
mad24	Contains two potential magnetite-binding regions in N-, and C-terminals and a SMC domain. May play roles in the assembly and arrangement of magnetosome bundle structures.	Bioinformatics analysis	This study
mad25	Contains an ATPase domain affiliated with the SMC super family. May be a subunit of the ATPase which provides energy for assembling magnetosomes into chain structures.	Bioinformatics analysis	This study
mad26	Contains an ATPase domain affiliated with the SMC super family. May be a subunit of the ATPase which provides energy for assembling magnetosomes into chain structures.	Bioinformatics analysis	This study
mad27	Contains an AAA ATPase (ATPases associated with diverse cellular activity) domain. May provide energy for assembling magnetosomes into chain structures.	Bioinformatics analysis	This study
mad28	Homologous gene of *mamK*. May be involved in organizing magnetosomes into chain or bundle structures.	Bioinformatics analysis	[89]
mad29	Contains an AAA ATPase (ATPases associated with diverse cellular activity) domain. May provide energy for assembling magnetosomes into chain structures.	Bioinformatics analysis	This study
mad30	Homologous gene of *feoB* may play a role in ferrous ion transport.	Bioinformatics analysis	[89]
man1	Magnetite-binding protein may be involved in the morphological control of curved bullet-shaped magnetic particles.	Bioinformatics analysis	This study
man2	Homologous protein of MamL. May be involved in the magnetosome membrane formation.	Bioinformatics analysis	This study
man3	Contains a hydrophilic C-terminal and a transmembrane domain. May be a magnetite-binding protein.	Bioinformatics analysis	This study
man4	Contains a hydrophilic C-terminal. May be a magnetite-binding protein.	Bioinformatics analysis	This study
man5	Contains two potential magnetite-binding regions in N-, and C-terminals and a SMC domain. May play roles in the assembly and arrangement of magnetosome bundle structures.	Bioinformatics analysis	This study
man6	Contains an ATPase domain affiliated with the SMC super family. May provide energy for assembling magnetosomes into chain structures.	Bioinformatics analysis	This study

Table S8. Discussed or predicted key genes for magnetosome vesicle formation, protein sorting, and iron transport.

**Magnetosome biosynthesis processes**		**Magnetosome proteins in**			**References**
		Proteobacteria	***Desulfobacterota***	***Nitrospirae***	
Magnetosome membrane formation	Induce membrane curvature	*mamB*	*mamB*	*mamB*	[71, 77]
	Magnetosome vesicle formation	*mamILQ*	*mamILQ*	*mamIQ*, *man2*	[71, 79]
Protein sorting	Protein sorting	*mamAE*	*mamA*, ***mad23***	*mamAE*, ***mad23***	[69, 74, This study]
Iron transport and magnetite nucleation	Iron transport	*mamBHM*(*Z*)	*mamBM*, *mad17*, *mad30*	*mamBM*, (*mad17*), (*mad30*)	[70, 89]
	Nucleation of iron oxide particles	*mamO*	--	*mamO*	[83]
	PH control	*mamN*	*mamN*	Unknown	[79, 82, 90]
	Redox control	*mamEPT*(*XZ*)	*mamP*, *mamE-Cter*, *mad6*, ***mad9***	*mamEP*	[75, 84, This study]

Note: Genes in bold represent functions predicted in this study. Genes in brackets are only conserved in some strains.

Table S9. Discussed or predicted key genes for magnetite crystal growth.

Magnetite crystal growth processes	Magnetosome proteins involved in magnetite biosynthesis				References
	(Cubo)-octahedron	Prism	Bullet	Curved bullet	
Crystal number control	*mamP*(*R*)	*mamP*(*R*)	*mamP*, *mamE-Cter*	*mamP*	[84, 90]
Crystal size control	*mamFPST*, *mms-F*, *-6* (*mamCDGR*, *mms-5*, ***-6-L***, *-36*, *-48*)	*mamDFPST*, *mmsF* (*mamCR*, *mms-5*, *-6*, ***-6-L***)	***mad-4***, ***-8***, *-10*, *-11*, (***-3***, ***-5***, ***-19***)	***man-1***, ***-3***, ***-4***, (*mad10*)	[72, 73, 90, 91, This study]
Crystal morphology control	*mms6*, (*mamCD*, *mms-5*, ***-6-L***)	*mamD*, (*mamC*, *mms-5*, *-6*, ***-6-L***)	*mad-1*, *-2*	***man-1*, *-3*, *-4*,** *mad2*	[73, 90, This study]

Note: Genes in bold represent functions predicted in this study. Genes in brackets are only conserved in some strains.

Table S10. Discussed or predicted key gene in assembly of magnetosome chain configurations.

Processes in magnetosome chain configuration assembly	Proteins involved in magnetosome chain configuration assembly in												References
	**Alphaproteobacteria**		**Gammaproteobacteria**	**Ca. 'Etaproteobacteria'**				**Desulfobacterota**			**Nitrospirota**		
	**Sub-chains**	**Single chain**	**A longer linear chain**	**Single chain**	**Double chains**	**Two double chains**	**Non-linear chain**	**Single chain**	**Single bundle**	**Multiple bundles**	**Multiple bundles**	**Single bundle**	
Organize magnetosomes into chain configurations	*mcaA*, *mamK*	*mamK*	**Multiple copies of *mamK***	***mamK***	**Multiple copies of *mamK***	**Multiple copies of *mamK***	**Multiple copies of *mamK***	***mamK*, *mad28***	***mamK*, *mad28***	**Multiple copies of *mamK* and *mad28***	***mamK*, *mad28***	***mamK*, *mad28***	[81, 89, This study]
Assist interactions between magnetosomes and MamK proteins	(*mamJ*)	(*mamJ*)	--	--	--	--	--	--	--	--	--	--	[80]
Anchor magnetosomes onto cytomembranes	*mcaA* *mamY*	*mamY*	--	--	--	--	--	--	--	--	--	--	[86]
Provide energy for chain bundle assembly	--	--	--	--	--	--	--	--	***mad-22*, -*25*, -*26*, -*27*, -*29***	***mad-22*, -*25*, -*26*, -*27***	***mad-22*, -*24*, -*25*, -*26*, *man6***	***mad-22*, -*24*, -*25*, -*26*, *man6***	[This study]
Assemble magnetosomes into chain bundles	--	--	--	--	--	--	--	--	***mad24***	***mad24***	***man5***	***man5***	[This study]
Control number of magnetosome chain/bundles	***MamY* and copy number of *mamK***		**The adjacent organization of the two *mamK* copies**	**Copy number and encoding protein similarity of *mamK* multiple copies**				**Copy number of *mamK* and *mad28***			**Unknown**		[This study]

Note: Genes in bold represent functions predicted in this study. Genes in brackets are only conserved in some strains.

Table S11. FISH probes used in this study.

Probe name	Target group	Oligonucleotide sequence (5' to 3')	Positions	T_m (°C)	Formamide conc.	Mismatched sequence number	Reference or source
EUB338	Most bacteria	GCTGCCTCCCGTAGGAGT	338–355	64	35%	--	[92]
YQC9-115	YQC-9	TTGTCCCCCATCGCAGGGCA	115-134	66	40%	0	This study
XQGC1-539	XQGC-1	GAGGATTTCACTTCTGACTTAAA	539-560	56	20%	2	
MYC9-924	MYC-9	GAGGATTTCACTCCTGACTTGAA	924-946	58	25%	1	
YQC5-911	YQC-5	TCCTGACTTATATAACCGCC	911-930	58	25%	2	
WYHS4-1217	WYHS-4	TTGGCTTCGCAGCCTCGCAA	1217-1236	64	35%	0	
YQV1-195	YQV-1	CCCTTCCTCAAGCGACTTGC	195-214	64	35%	0	
MYC10-60	MYC-10	GTTACCCCTCCATAACTCCG	60-79	62	30%	2	
YQR1-1423	YQR-1	TGCACATGTATTGCTACATGTACA	1423-1446	58	25%	0	

Notes: Species-specific oligonucleotide probes were designed using the offline tool DNAMAN (Version 7.0, Lynnon Biosoft, USA) and were synthesized by the Huada Genome Center, Beijing, China. The corresponding melting temperature (T_m) was measured directly during probe synthesis, and the formamide concentration was calculated where concentration = (T_m - 46) × 2. Four probes (XQGC1-539, MYC9-924, YQC5-911, and MYC10-60) could also match other 16S rRNA sequences and were, therefore, not strictly species-specific. However, further analysis indicates that these mismatched sequences are either identical to our targets (16S rRNA gene sequence identity >97%) or that they belong to non-MTB species. Although some mismatched sequences come from other MTB species, they were not detected from our samples. Therefore, these four probes were used here. For more detailed information, see Table S12.

Table S12. Mismatched information of FISH probes used in this study.

Probe name	No. of mismatched sequences	Name of mismatched sequence	Accession	Identity with target group (%)	Taxon
XQGC1-539	2	OTU51	GQ468517	99.7	'Ca. Etaproteobacteria'
		OTU15	GQ468514	96.5	
MYC9-924	1	OTU17	EU780677	99.8	'Ca. Etaproteobacteria'
YQC5-911	2	D896293	FJ959680	75.8	Bacteroidetes
		MP 17	X61607	92.3	'Ca. Etaproteobacteria'
MYC10-60	2	OTU50	GQ468511	94.1	'Ca. Etaproteobacteria'
		MY3-11A	HM454282	99.9	Nitrospirae

Notes: The specificity of all probes was evaluated using the online probe evaluation tools ProbeMatch [6]. Mismatched sequences were downloaded from the RDP database; those shorter than 1,200 bp and repetitive sequences (identity ⩾ 99.0%) were removed. Two 16S rRNA sequences match XQGC1-539. However, one magnetotactic cocci sequence OTU51 has high identity (⩾ 97.0%) with strain XQGC-1 and may be affiliated with the same species. The other '*Ca.* Etaproteobacteria' strain OTU15 16S rRNA sequence matching XQGC1-539 was not found in this sample. The '*Ca.* Etaproteobacteria' sequence OTU17 has high identity (⩾ 97.0%) with strain MYC-9 and may be affiliated with the same species. Two 16S rRNA sequences match YQC5-532. One 16S rDNA sequence D896293 belonging to *Bacteroidetes* was not MTB. The other '*Ca.* Etaproteobacteria' strain MP 17 was not found in this sample. Two 16S rRNA sequences match with DMHC9-60. One 16S rDNA sequence OTU50 was not found in this sample. The other MTB MY3-11A has high identity (⩾ 97.0%) with strain MYC-10 and may be affiliated with the same species.

Supplementary references

1. Liu P, Liu Y, and Zhao X *et al.* Diverse phylogeny and morphology of magnetite biomineralized by magnetotactic cocci. *Environ Microbiol* 2021; **23**: 1115-29.
2. Schüler D. The biomineralization of magnetosomes in *Magnetospirillum gryphiswaldense*. *Int Microbiol* 2002; **5**: 209-14.
3. Li J, Pan Y, and Liu Q *et al.* Biomineralization, crystallography and magnetic properties of bullet-shaped magnetite magnetosomes in giant rod magnetotactic bacteria. *Earth Planet Sci Lett* 2010; **293**: 368-76.
4. Li J, Zhang H, and Menguy N *et al.* Single-cell resolution of uncultured magnetotactic bacteria via fluorescence-coupled electron microscopy. *Appl Environ Microbiol* 2017; **83**: e00409-17.
5. Yarza P, Yilmaz P, and Pruesse E *et al.* Uniting the classification of cultured and uncultured bacteria and archaea using 16S rRNA gene sequences. *Nat Rev Microbiol* 2014; **12**: 635-45.
6. Kim Y, Teletia N, and Ruotti V *et al.* ProbeMatch: rapid alignment of oligonucleotides to genome allowing both gaps and mismatches. *Bioinformatics* 2009; **25**: 1424-5.
7. Bolger AM, Lohse M and Usadel B. Trimmomatic: a flexible trimmer for Illumina sequence data. *Bioinformatics* 2014; **30**: 2114-20.
8. Peng Y, Leung HCM, and Yiu SM *et al.* IDBA-UD: a de novo assembler for single-cell and metagenomic sequencing data with highly uneven depth. *Bioinformatics* 2012; **28**: 1420-8.
9. Uritskiy GV, DiRuggiero J and Taylor J. MetaWRAP-a flexible pipeline for genome-resolved metagenomic data analysis. *Microbiome* 2018; **6**: 158.
10. Parks DH, Imelfort M, and Skennerton CT *et al.* CheckM: assessing the quality of microbial genomes recovered from isolates, single cells, and metagenomes. *Genome Res* 2015; **25**: 1043-55.
11. Richter M and Rosselló-Móra R. Shifting the genomic gold standard for the prokaryotic species definition. *Proc Natl Acad Sci USA* 2009; **106**: 19126-31.
12. Besemer J, Lomsadze A and Borodovsky M. GeneMarkS: a self-training method for prediction of gene starts in microbial genomes. Implications for finding sequence motifs in regulatory regions. *Nucleic Acids Res* 2001; **29**: 2607-18.
13. Altschul SF, Madden TL, and Schäffer AA *et al.* Gapped BLAST and PSI-BLAST: a new generation of protein database search programs. *Nucleic Acids Res* 1997; **25**: 3389-402.

14. Chaumeil PA, Mussig AJ, and Hugenholtz P *et al.* GTDB-Tk: a toolkit to classify genomes with the Genome Taxonomy Database. *Bioinformatics* 2019; **36**: 1925-7.
15. Nguyen LT, Schmidt HA, and von Haeseler A *et al.* IQ-TREE: a fast and effective stochastic algorithm for estimating maximum-likelihood phylogenies. *Mol Biol Evol* 2015; **32**: 268-74.
16. Kalyaanamoorthy S, Minh BQ, and Wong TKF *et al.* ModelFinder: fast model selection for accurate phylogenetic estimates. *Nat Methods* 2017; **14**: 587-9.
17. Edgar RC. MUSCLE: multiple sequence alignment with high accuracy and high throughput. *Nucleic Acids Res* 2004; **32**: 1792-7.
18. Lu S, Wang J, and Chitsaz F *et al.* CDD/SPARCLE: the conserved domain database in 2020. *Nucleic Acids Res* 2020; **48**: D265-D8.
19. Jumper J, Evans R, and Pritzel A *et al.* Highly accurate protein structure prediction with AlphaFold. *Nature* 2021; **596**: 583-9.
20. Baek M, DiMaio F, and Anishchenko I *et al.* Accurate prediction of protein structures and interactions using a three-track neural network. *Science* 2021; **373**: 871-6.
21. Kaneko T, Minamisawa K, and Isawa T *et al.* Complete genomic structure of the cultivated rice endophyte *Azospirillum* sp. B510. *DNA Res* 2010; **17**: 37-50.
22. Liu P, Liu Y, and Ren X *et al.* A novel magnetotactic alphaproteobacterium producing intracellular magnetite and calcium-bearing minerals. *Appl Environ Microbiol* 2021; **87**: e01556-21.
23. Koziaeva VV, Rusakova SA, and Slobodova NV *et al.* *Magnetospirillum kuznetsovii* sp. nov., a novel magnetotactic bacterium isolated from a lake in the Moscow region. *Int J Syst Evol Microbiol* 2019; **69**: 1953-9.
24. Lin W and Pan Y. Temporal variation of magnetotactic bacterial communities in two freshwater sediment microcosms. *FEMS Microbiol Lett* 2010; **302**: 85-92.
25. Lin W, Li J, and Schüler D *et al.* Diversity analysis of magnetotactic bacteria in Lake Miyun, northern China, by restriction fragment length polymorphism. *Syst Appl Microbiol* 2009; **32**: 342-50.
26. Lin W, Jogler C, and Schüler D *et al.* Metagenomic analysis reveals unexpected subgenomic diversity of magnetotactic bacteria within the phylum Nitrospirae. *Appl Environ Microbiol* 2011; **77**: 323-6.
27. Spring S, Amann R, and Ludwig W *et al.* Phylogenetic diversity and identification of nonculturable magnetotactic bacteria. *Syst Appl Microbiol* 1992; **15**: 116-22.
28. Koziaeva V, Dziuba M, and Leão P *et al.* Genome-based metabolic reconstruction of a novel uncultivated freshwater magnetotactic coccus

"*Ca*. Magnetaquicoccus inordinatus" UR-1, and proposal of a candidate family "Ca. Magnetaquicoccaceae". *Front Microbiol* 2019; **10**: 2290.
29. Liu P, Tamaxia A, and Liu Y *et al.* Identification and characterization of magnetotactic Gammaproteobacteria from a salt evaporation pool, Bohai Bay, China. *Environ Microbiol* 2022; **24**: 938-50.
30. Lima LJ, Kamphuis HJ, and Nout MR *et al.* Microbiota of cocoa powder with particular reference to aerobic thermoresistant spore-formers. *Food Microbiol* 2011; **28**: 573-82.
31. Morillo V, Abreu F, and Araujo AC *et al.* Isolation, cultivation and genomic analysis of magnetosome biomineralization genes of a new genus of south-seeking magnetotactic cocci within the Alphaproteobacteria. *Front Microbiol* 2014; **5**: 72.
32. Meldrum FC, Mann S, and Heywood BR *et al.* Electron microscopy study of magnetosomes in a cultured coccoid magnetotactic bacterium. *Proc R Soc Lond B* 1993; **251**: 231-6.
33. Schübbe S, Williams TJ, and Xie G *et al.* Complete genome sequence of the chemolithoautotrophic marine magnetotactic coccus strain MC-1. *Appl Environ Microbiol* 2009; **75**: 4835-52.
34. Ji B, Zhang S, and Zhang W *et al.* The chimeric nature of the genomes of marine magnetotactic coccoid-ovoid bacteria defines a novel group of Proteobacteria. *Environ Microbiol* 2017; **19**: 1103-19.
35. Lefèvre CT, Bernadac A, and Yu-Zhang K *et al.* Isolation and characterization of a magnetotactic bacterial culture from the Mediterranean Sea. *Environ Microbiol* 2009; **11**: 1646-57.
36. Matsunaga T, Okamura Y, and Fukuda Y *et al.* Complete genome sequence of the facultative anaerobic magnetotactic bacterium *Magnetospirillum* sp. strain AMB-1. *DNA Res* 2005; **12**: 157-66.
37. Li J and Pan Y. Environmental factors affect magnetite magnetosome synthesis in *Magnetospirillum magneticum* AMB-1: implications for biologically controlled mineralization. *Geomicrobiol J* 2012; **29**: 362-73.
38. Wang Y, Zhang T, and Lin W *et al.* Complete genome sequence of *Magnetospirillum* sp. strain XM-1, isolated from the Xi'an city moat, China. *Genome Announc* 2016; **4**: e01171-16
39. Wang Y, Lin W, and Li J *et al.* Characterizing and optimizing magnetosome production of *Magnetospirillum* sp. XM-1 isolated from Xi'an City Moat, China. *FEMS Microbiol Lett* 2015; **362**: fnv167.
40. Smalley MD, Marinov GK, and Bertani LE *et al.* Genome sequence of *Magnetospirillum magnetotacticum* strain MS-1. *Genome Announc* 2015; **3**: e00233-15.
41. Maratea D and Blakemore R. *Aquaspirillum magnetotacticum* sp. nov., a magnetic spirillum. *Int J Syst Evol Microbiol* 1981; **31**: 452-5.

42. Ke LF, Liu PM, and Liu S *et al.* Complete genome sequence of *Magnetospirillum* sp. ME-1, a novel magnetotactic bacterium isolated from East Lake, Wuhan, China. *Genome Announc* 2017; **5**: e00485-17.
43. Ke L, Chen Y, and Liu P *et al.* Characteristics and optimised fermentation of a novel magnetotactic bacterium, *Magnetospirillum* sp. ME-1. *FEMS Microbiol Lett* 2018; **365**: fny052.
44. Dziuba M, Koziaeva V, and Grouzdev D *et al. Magnetospirillum caucaseum* sp. nov., *Magnetospirillum marisnigri* sp. nov. and *Magnetospirillum moscoviense* sp. nov., freshwater magnetotactic bacteria isolated from three distinct geographical locations in European Russia. *Int J Syst Evol Microbiol* 2016; **66**: 2069-77.
45. Wang X, Wang Q, and Zhang W *et al.* Complete genome sequence of *Magnetospirillum gryphiswaldense* MSR-1. *Genome Announc* 2014; **2**: 4899-910.
46. Fdez-Gubieda ML, Muela A, and Alonso J *et al.* Magnetite biomineralization in *Magnetospirillum gryphiswaldense*: time-resolved magnetic and structural studies. *ACS Nano* 2013; **7**: 3297-305.
47. Trubitsyn D, Abreu F, and Ward FB *et al.* Draft genome sequence of *Magnetovibrio blakemorei* Strain MV-1, a marine vibrioid magnetotactic bacterium. *Genome Announc* 2016; **4**: e01330-16.
48. Bazylinski DA, Williams TJ, and Lefèvre CT *et al. Magnetovibrio blakemorei* gen. nov., sp. nov., a magnetotactic bacterium (Alphaproteobacteria: Rhodospirillaceae) isolated from a salt marsh. *Int J Syst Evol Microbiol* 2013; **63**: 1824-33.
49. Monteil CL, Perrière G, and Menguy N *et al.* Genomic study of a novel magnetotactic Alphaproteobacteria uncovers the multiple ancestry of magnetotaxis. *Environ Microbiol* 2018; **20**: 4415-30.
50. Du H, Zhang W, and Zhang W *et al.* Magnetosome gene duplication as an important driver in the evolution of magnetotaxis in the Alphaproteobacteria. *mSystems* 2019; **4**: e00315-19.
51. Lefèvre CT and Bazylinski DA. Ecology, diversity, and evolution of magnetotactic bacteria. *Mol Microbiol* 2013; **77**: 497-526.
52. Zhu K, Pan H, and Li J *et al.* Isolation and characterization of a marine magnetotactic spirillum axenic culture QH-2 from an intertidal zone of the China Sea. *Res Microbiol* 2010; **161**: 276-83.
53. Lefèvre CT, Viloria N, and Schmidt ML *et al.* Novel magnetite-producing magnetotactic bacteria belonging to the Gammaproteobacteria. *ISME J* 2012; **6**: 440-50.
54. Geurink C, Lefevre CT, and Monteil CL *et al.* Complete genome sequence of strain BW-2, a magnetotactic Gammaproteobacterium in the family Ectothiorhodospiraceae, isolated from a brackish spring in Death Valley, California. *Microbiol Resour Announc* 2020; **9**: e01144-19.

55. Trubitsyn D, Monteil CL, and Geurink C *et al.* Complete genome sequence of strain SS-5, a magnetotactic Gammaproteobacterium isolated from the Salton Sea, a shallow, saline, endorheic rift lake located on the San Andreas Fault in California. *Microbiol Resour Announc* 2021; **10**: e00928-20.
56. Nakazawa H, Arakaki A, and Narita-Yamada S *et al.* Whole genome sequence of *Desulfovibrio magneticus* strain RS-1 revealed common gene clusters in magnetotactic bacteria. *Genome Res* 2009; **19**: 1801-8.
57. Shimoshige H, Kobayashi H, and Shimamura S *et al.* Isolation and cultivation of a novel sulfate-reducing magnetotactic bacterium belonging to the genus *Desulfovibrio*. *PLoS ONE* 2021; **16**: e0248313.
58. Kolinko S, Richter M, and Gloeckner F-O *et al.* Single-cell genomics reveals potential for magnetite and greigite biomineralization in an uncultivated multicellular magnetotactic prokaryote. *Environ Microbiol Rep* 2014; **6**: 524-31.
59. Zhang R, Chen Y, and Du H *et al.* Characterization and phylogenetic identification of a species of spherical multicellular magnetotactic prokaryotes that produces both magnetite and greigite crystals. *Res Microbiol* 2014; **165**: 481-9.
60. Lefèvre CT, Menguy N, and Abreu F *et al.* A cultured greigite-producing magnetotactic bacterium in a novel group of sulfate-reducing bacteria. *Science* 2011; **334**: 1720-3.
61. Kolinko S, Richter M, and Glöckner F-O *et al.* Single-cell genomics of uncultivated deep-branching magnetotactic bacteria reveals a conserved set of magnetosome genes. *Environ Microbiol* 2016; **18**: 21-37.
62. Li J, Menguy N, and Gatel C *et al.* Crystal growth of bullet-shaped magnetite in magnetotactic bacteria of the *Nitrospirae* phylum. *J R Soc Interface* 2015; **12**: 20141288.
63. Lin W, Deng A, and Wang Z *et al.* Genomic insights into the uncultured genus '*Candidatus* Magnetobacterium' in the phylum Nitrospirae. *ISME J* 2014; **8**: 2463-77.
64. Zhang W, Wang Y, and Liu L *et al.* Identification and genomic characterization of two previously unknown magnetotactic Nitrospirae. *Front Microbiol* 2021; **12**: 690052.
65. Uzun M, Koziaeva V, and Dziuba M *et al.* Detection of interphylum transfers of the magnetosome gene cluster in magnetotactic bacteria. *Front Microbiol* 2022; **13**: 945734.
66. Li J, Liu P, and Menguy N *et al.* Intracellular silicification by early-branching magnetotactic bacteria. *Sci Adv* 2022; **8**: eabn6045.
67. Kolinko S, Jogler C, and Katzmann E *et al.* Single-cell analysis reveals a novel uncultivated magnetotactic bacterium within the candidate division OP3. *Environ Microbiol* 2012; **14**: 1709-21.

68. Konstantinidis KT, Rosselló-Móra R and Amann R. Uncultivated microbes in need of their own taxonomy. *ISME J* 2017; **11**: 2399-406.
69. Komeili A, Vali H, and Beveridge TJ *et al.* Magnetosome vesicles are present before magnetite formation, and MamA is required for their activation. *Proc Natl Acad Sci USA* 2004; **101**: 3839-44.
70. Uebe R, Junge K, and Henn V *et al.* The cation diffusion facilitator proteins MamB and MamM of *Magnetospirillum gryphiswaldense* have distinct and complex functions, and are involved in magnetite biomineralization and magnetosome membrane assembly. *Mol Microbiol* 2011; **82**: 818-35.
71. Murat D, Quinlan A, and Vali H *et al.* Comprehensive genetic dissection of the magnetosome gene island reveals the step-wise assembly of a prokaryotic organelle. *Proc Natl Acad Sci USA* 2010; **107**: 5593-8.
72. Scheffel A, Gardes A, and Grunberg K *et al.* The major magnetosome proteins MamGFDC are not essential for magnetite biomineralization in *Magnetospirillum gryphiswaldense* but regulate the size of magnetosome crystals. *J Bacteriol* 2008; **190**: 377-86.
73. Arakaki A, Yamagishi A, and Fukuyo A *et al.* Co-ordinated functions of Mms proteins define the surface structure of cubo-octahedral magnetite crystals in magnetotactic bacteria. *Mol Microbiol* 2014; **93**: 554-67.
74. Wan J, Browne PJ, and Hershey DM *et al.* A protease-mediated switch regulates the growth of magnetosome organelles in *Magnetospirillum magneticum*. *Proc Natl Acad Sci USA* 2022; **119**: e2111745119.
75. Quinlan A, Murat D, and Vali H *et al.* The HtrA/DegP family protease MamE is a bifunctional protein with roles in magnetosome protein localization and magnetite biomineralization. *Mol Microbiol* 2011; **80**: 1075-87.
76. Rawlings AE, Bramble JP, and Walker R *et al.* Self-assembled MmsF proteinosomes control magnetite nanoparticle formation *in vitro*. *Proc Natl Acad Sci USA* 2014; **111**: 16094-9.
77. Uebe R and Schüler D. Magnetosome biogenesis in magnetotactic bacteria. *Nat Rev Microbiol* 2016; **14**: 621-37.
78. Raschdorf O, Müller FD, and Pósfai M *et al.* The magnetosome proteins MamX, MamZ and MamH are involved in redox control of magnetite biomineralization in *Magnetospirillum gryphiswaldense*. *Mol Microbiol* 2013; **89**: 872-86.
79. Lohße A, Borg S, and Raschdorf O *et al.* Genetic dissection of the *mamAB* and *mms6* operons reveals a gene set essential for magnetosome biogenesis in *Magnetospirillum gryphiswaldense*. *J Bacteriol* 2014; **196**: 2658-69.
80. Scheffel A, Gruska M, and Faivre D *et al.* An acidic protein aligns magnetosomes along a filamentous structure in magnetotactic bacteria. *Nature* 2006; **440**: 110-4.
81. Komeili A, Li Z, and Newman DK *et al.* Magnetosomes are cell membrane invaginations organized by the actin-like protein MamK. *Science*

2006; **311**: 242-5.
82. Schüler D. Genetics and cell biology of magnetosome formation in magnetotactic bacteria. *FEMS Microbiol Rev* 2008; **32**: 654-72.
83. Hershey DM, Ren X, Melnyk RA et al. MamO is a repurposed serine protease that promotes magnetite biomineralization through direct transition metal binding in magnetotactic bacteria. *PLoS Biol* 2016; **14**: e1002402.
84. Siponen MI, Legrand P, and Widdrat M *et al.* Structural insight into magnetochrome-mediated magnetite biomineralization. *Nature* 2013; **502**: 681-4.
85. Yang J, Li S, and Huang X *et al.* MamX encoded by the *mamXY* operon is involved in control of magnetosome maturation in *Magnetospirillum gryphiswaldense* MSR-1. *BMC Microbiol* 2013; **13**: 1-10.
86. Toro-Nahuelpan M, Giacomelli G, and Raschdorf O *et al.* MamY is a membrane-bound protein that aligns magnetosomes and the motility axis of helical magnetotactic bacteria. *Nat Microbiol* 2019; **4**: 1978–89.
87. Arakaki A, Webb J and Matsunaga T. A novel protein tightly bound to bacterial magnetic particles in *Magnetospirillum magneticum* strain AMB-1. *J Biol Chem* 2003; **278**: 8745-50.
88. Wan J, Monteil CL, and Taoka A *et al.* McaA and McaB control the dynamic positioning of a bacterial magnetic organelle. *BioRxiv* 2022.
89. Lefèvre CT, Trubitsyn D, and Abreu F *et al.* Comparative genomic analysis of magnetotactic bacteria from the Deltaproteobacteria provides new insights into magnetite and greigite magnetosome genes required for magnetotaxis. *Environ Microbiol* 2013; **15**: 2712-35.
90. Rahn-Lee L, Byrne ME, and Zhang M *et al.* A genetic strategy for probing the functional diversity of magnetosome formation. *PLoS Genet* 2015; **11**: e1004811.
91. Pohl A, Young SAE, and Schmitz TC *et al.* Magnetite-binding proteins from the magnetotactic bacterium *Desulfamplus magnetovallimortis* BW-1. *Nanoscale* 2021; **13**: 20396-400.
92. Amann RI, Krumholz L and Stahl DA. Fluorescent-oligonucleotide probing of whole cells for determinative, phylogenetic, and environmental studies in microbiology. *J Bacteriol* 1990; **172**: 762-70.
